# Epidemiological Study of Childhood Idiopathic Epilepsy from 1990 to 2021 at Global, Regional, and National Scales

**DOI:** 10.1016/j.mayocpiqo.2025.100641

**Published:** 2025-06-25

**Authors:** Li Wang, Lei Tang, Ji Zhang, Ye Li, Feng Zhang, Qiaoling Tang, Siyuan Ma, Ran Liu, Xiangbin Zhang, Sai Wang, Yupeng Zhang, Lei Chen, Junyi Ma, Xuelun Zou, Tianxing Yao, Rongmei Tang, Yexiang Yi, Yi Zeng, Duolao Wang, Le Zhang

**Affiliations:** a Department of Neurology, Xiangya Hospital, Central South University, Jiangxi, Nanchang, China; b Department of Neurology, Xiangya Hospital, Central South University, Changsha, China; c Multi-Modal Monitoring Technology for Severe Cerebrovascular Disease of Human Engineering Research Center, Changsha, China; d Brain Health Center of Hunan Province, Changsha, China; e Human Brain Disease Biological Resources Platform of Hunan Province, Changsha, China; f National Clinical Research Center for Geriatric Disorders, Xiangya Hospital, Central South University, Changsha, China; g FuRong Laboratory, Changsha, China; h Department of Cardiovascular Medicine, Xiangya Hospital, Central South University, Changsha, China; i Department of Geriatrics, Second Xiangya Hospital, Central South University, Changsha, China; j Department of Clinical Sciences, Liverpool School of Tropical Medicine, United Kingdom

## Abstract

**Objective:**

To address the long-term impact of childhood idiopathic epilepsy on health and families, and to provide epidemiological evidence for developing effective prevention and treatment strategies, this study aimed to explore the trends in incidence, deaths, and disability-adjusted life years (DALYs) of childhood idiopathic epilepsy globally and across regions from 1990 to 2021.

**Patients and Methods:**

This cross-sectional analysis utilized data from the 2021 Global Burden of Disease database, covering idiopathic epilepsy cases among children aged 0-14 years across 204 countries and regions. The study period was from September 15, 2024, to October 31, 2024. Key indicators included incidence, deaths (all-cause and specific), and DALYs, with trend analysis conducted using the exponential annual percentage change (EAPC). All analyses were stratified by region, country, gender, and sociodemographic index (SDI).

**Results:**

In 2021, there were 1,227,191 new cases of childhood idiopathic epilepsy globally (95% uncertainty interval [UI], 786,363-1,734,488). From 1990 to 2021, the total number of cases increased by 26.3% (95% UI, 6.8%-51.2%), with the incidence rising from 55.85 per 100,000 population to 60.998, and an EAPC of 0.2% (95% CI, 0.17-0.23). Deaths decreased by 29.5%, from 25,768 to 18,171, with the death rate dropping from 1.482 per 100,000 to 0.903 and an EAPC of −1.39% (95% CI, −1.48 to −1.3). DALYs decreased by 14.90%, reaching 3,564,497 in 2021 (95% UI, 2,700,944-4,753,410), with an EAPC of −0.94% (95% CI, −1.0 to −0.89). Low SDI regions bore the highest burden, with the highest death rate (1.459 per 100,000 in 2021). Regionally, tropical Latin America saw the fastest growth in incidence (EAPC 0.29), whereas Tajikistan had the highest death rate (2.766 per 100,000), and Taiwan Province of China had the highest DALY rate (99.718 per 100,000).

**Conclusion:**

Childhood idiopathic epilepsy remains a significant global health challenge, with an increasing incidence. Despite a decline in global deaths and DALYs, the disease burden in low SDI regions remains substantial. Understanding the epidemiological characteristics of childhood idiopathic epilepsy is critical for developing effective prevention and management strategies. The findings highlight the importance of targeted interventions in resource-limited settings to bridge the gap in treatment outcomes for childhood epilepsy globally.

Idiopathic epilepsy is defined as recurrent seizures without an identifiable cause, often associated with genetic predisposition and specific gene mutations. Typically onset in childhood or adolescence, it may resolve spontaneously in adulthood. Epidemiological data indicate a global prevalence of 4-10 cases per 1000 children, with idiopathic epilepsy accounting for ∼40% of all epilepsy cases.^1^ Although the course of childhood idiopathic epilepsy is usually benign, early-onset cases may face higher risks of developmental and cognitive impairments, such as attention deficits and learning difficulties.^2^

Recent research into epileptogenesis has emphasized the role of genetic factors. Mutations in genes such as SCN1A and PCDH19 are closely linked to the pathogenesis of idiopathic epilepsy.^3^ Early diagnosis and intervention are critical for mitigating the negative impact of epilepsy on cognition, behavior, and psychology. Timely antiepileptic treatment can significantly improve quality of life and reduce complications.^4^

The Global Burden of Disease (GBD) study provides reliable estimates for the epidemiology of childhood epilepsy by analyzing incidence, deaths, and Disability-Adjusted Life Years (DALYs).^5^ From 1990 to 2021, the number of children with idiopathic epilepsy increased by 255,823, with incidence rates higher in low-income and middle-income countries, likely due to limited health care resources and prenatal care.^6^ Childhood idiopathic epilepsy is a key target for reducing the burden of noncommunicable diseases in children. Regular reassessment of the GBD of childhood idiopathic epilepsy is crucial for prevention and control efforts. To our knowledge, no long-term global trends in childhood idiopathic epilepsy epidemiology have been reported. Here, we analyzed trends in incidence, deaths, and DALYs of childhood idiopathic epilepsy from 1990 to 2021 using the GBD database.

## Patients and Methods

### Overview and Data Collection

This cross-sectional study was approved by Xiangya Hospital of Central South University. The hospital’s ethics committee waived informed consent as the study involved only data analysis without identifiable personal information. Data on idiopathic epilepsy in children aged 0-14 years were collected from the GBD database.^7^

The study conducted in 2021, part of the GBD initiative, evaluated the incidence, death rates, and DALYs for 371 diseases and injuries across 204 countries and regions from 1990 to 2021. The analysis also included uncertainty intervals related to the incidence data.^7^ To report the age distribution of pediatric idiopathic epilepsy, patients were divided into 4 categories: infants under 1 year, 1-4 years, 5-9 years, and 10-14 years. In this research, we gathered data on the prevalence and incidence of childhood idiopathic epilepsy, and the number of deaths attributed to it and the associated DALYs, along with their respective incidences on global, regional, and national scale. We employed linear regression analysis to determine the average exponential annual percentage change (EAPC).^8^ The study adhered to the protocols set forth by the Strengthening the Reporting of Observational Studies in Epidemiology (STROBE). The study period was from September 15, 2024, to October 31, 2024.

### Sociodemographic Index

The sociodemographic index (SDI) measures a country or region’s development level based on fertility rates, education levels, and per capita income. The SDI ranges from 0 to 1, with higher levels indicating better socioeconomic development. In this study, countries and regions were divided into 5 SDI categories (low, low-middle, middle, middle-high, and high) to explore the association between childhood idiopathic epilepsy burden and socioeconomic development.^9^

### Statistical Analyses

The primary metrics employed to evaluate the burden of childhood idiopathic epilepsy consist of incidence, deaths, and DALYs, along with their corresponding rates. These rates are expressed per 100,000 individuals in accordance with GBD methodology and include 95% uncertainty intervals (UIs).^10^ The trends in childhood idiopathic epilepsy are analyzed by calculating the EAPC to detect variations in the disease burden over time^11^^,^^12^; The 95% confidence intervals (CIs) for the EAPCs are calculated using linear modeling methods.^13^ If the upper bound of the EAPC and its 95% CI is below zero, this suggests a decreasing trend in the associated rate. Conversely, if both the EAPC and the lower limit, which have a 95% CI are greater than zero, an increase in the rate is anticipated.^14^ Gaussian curves are utilized to investigate the association between the EAPC, the incidence of childhood idiopathic epilepsy, and the Human Development Index (HDI).^12^^,^^15^ All analyses were performed using RStudio, version 4.4.1. *P*-values were calculated as 2-sided, with values below 0.05 deemed statistically significant.^16^

## Results

### Idiopathic Epilepsy in Children: Global Trends

#### Incidence

**Figure 1:**
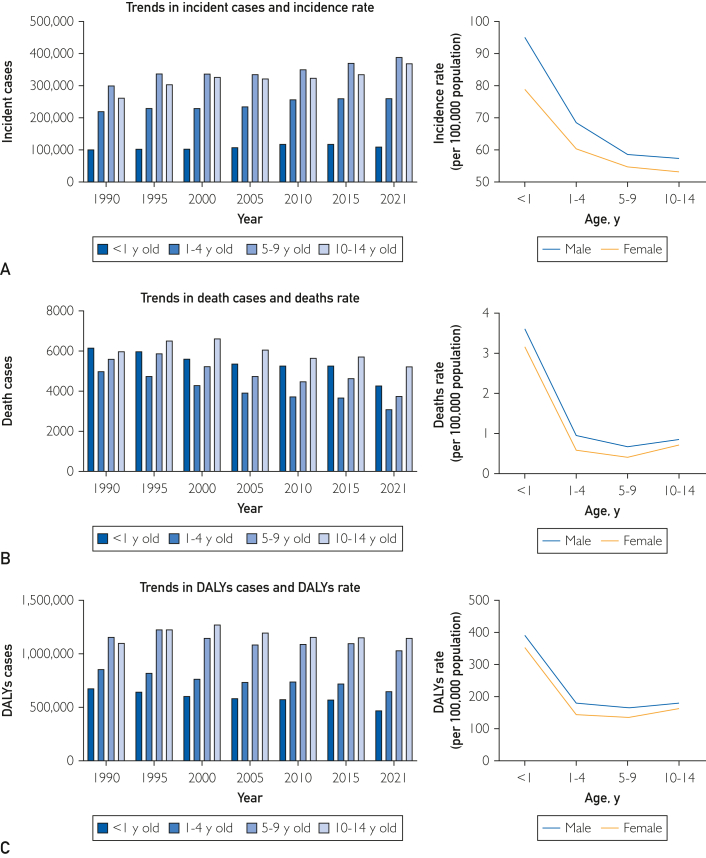
Trends in idiopathic epilepsy incidence, deaths, and DALYs among children from 1990 to 2021. A, trends in incident cases and incidence rate; B, trends in death cases and deaths rate; and C, trends in DALYs cases and DALYs rate. DALYs, disability-adjusted life years.

In 2021, there were 1,227,191 new cases of pediatric idiopathic epilepsy worldwide (95% UI, 786,363-1,734,488) (distribution can be seen in Supplemental Figure 1A, [available online at http://www.mcpiqojournal.org]). From 1990 to 2021, the number of cases increased by 26.3% (95% UI, 6.8%-51.2%), with the incidence rising from 55.853 (95% UI, 35.893-78.696) to 60.998 (95% UI, 39.086-86.213); EAPC was 0.2 (95% CI, 0.17-0.23). Incidence increased across almost all age groups, with the largest increase (28.97%) in children aged 10-14 years. In 1990 and 2021, children under 1 year had the highest incidence (95.04 and 78.88, respectively). In 2021, boys had a higher incidence than girls. The incidence of childhood idiopathic epilepsy decreased with increasing age (Figure 1A; Table).

**Table:** Incidence of Idiopathic Epilepsy in Children Between 1990 and 2021 at the Global and Regional Level

Location	Rate per 100,000 (95% UI)					
	1990 Incident cases	Incidence rate	2021 Incident cases	Incidence rate	1990-2021 Cases Change	EAPC^a^
Global	971,368 (624,226-1,368,636)	55.853 (35.893-78.696)	1,227,191 (786,363-1,734,488)	60.998 (39.086-86.213)	0.263 (0.068-0.512)	0.2 (0.17-0.23)
SDI						
High	119,260 (76,301-178,953)	64.184 (41.064-96.311)	121,918 (67,742-188,733)	70.662 (39.263-109.387)	0.022 (−0.182 to 0.214)	0.28 (0.23-0.33)
High middle	131,788 (82,694-188,453)	48.164 (30.222-68.873)	122,322 (72,912-188,316)	52.978 (31.579-81.561)	−0.072 (−0.275 to 0.162)	0.24 (0.17-0.3)
Middle SDI	312,668 (193,389-442,722)	54.169 (33.504-76.7)	334,092 (209,540-485,226)	58.937 (36.965-85.599)	0.069 (−0.137 to 0.344)	0.14 (0.1-0.19)
Low middle	255,940 (151,098-382,225)	54.212 (32.005-80.961)	341,687 (215,945-481,879)	58.928 (37.242-83.105)	0.335 (−0.009 to 0.856)	0.24 (0.2-0.28)
Low SDI	150,784 (80,733-230,379)	65.869 (35.268-100.64)	306,198 (185,486-438,447)	66.532 (40.303-95.268)	1.031 (0.588-1.825)	−0.11 (−0.14 to −0.08)
Regions						
Andean Latin America	13,265 (6002-21,312)	89.312 (40.414-143.492)	16,943 (8153-27,211)	93.636 (45.059-150.378)	0.277 (−0.383 to 1.801)	−0.53 (−0.66 to −0.39)
Australasia	3006 (1106-5062)	65.541 (24.113-110.379)	3632 (1343-6248)	63.371 (23.43-109.013)	0.208 (−0.516 to 1.899)	−0.27 (−0.31 to −0.22)
Caribbean	8300 (4565-12,611)	72.732 (40.002-110.498)	8270 (4333-12,886)	71.881 (37.657-112.002)	−0.004 (−0.402-0.586)	−0.05 (−0.08 to −0.02)
Central Asia	18,185 (10,470-27,657)	72.767 (41.895-110.668)	20,308 (11,099-30,457)	73.376 (40.103-110.048)	0.117 (−0.317 to 0.813)	−0.06 (−0.12 to −0.01)
Central Europe	18,777 (11,119-28,329)	63.687 (37.713-96.083)	11,674 (6917-17,585)	65.952 (39.077-99.346)	−0.378 (−0.54 to 0.16)	0.21 (0.17-0.24)
Central Latin America	61,379 (36,582-90,377)	95.337 (56.82-140.378)	57,735 (36,542-86,874)	90.943 (57.56-136.843)	−0.059 (−0.316 to 0.281)	−0.34 (−0.37 to −0.3)
Central Sub to Saharan Africa	21,551 (7516-38,447)	85.187 (29.708-151.973)	46,824 (17,837-81,338)	79.793 (30.397-138.609)	1.173 (−0.106 to 5.238)	0 (−0.09 to 0.08)
East Asia	102,872 (60,050-153,795)	31.189 (18.206-46.628)	102,941 (61,945-155,015)	38.504 (23.17-57.981)	0.001 (−0.284 to 0.386)	0.15 (0-0.31)
Eastern Europe	27,201 (16,827-40,172)	52.857 (32.698-78.061)	16,377 (9617-24,686)	46.205 (27.134-69.647)	−0.398 (−0.529 to 0.233)	−0.43 (−0.48 to −0.38)
Eastern Sub to Saharan Africa	68,996 (36,520-107,964)	76.179 (40.322-119.204)	137,606 (80,029-201,246)	77.12 (44.851-112.786)	0.994 (0.453-2.081)	−0.25 (−0.29 to −0.2)
High to income Asia Pacific	20,379 (11,274-31,574)	57.894 (32.029-89.7)	14,441 (8283-22,210)	64.395 (36.933-99.038)	−0.291 (−0.512-0.009)	0.2 (0.17-0.23)
High to income North America	34,225 (20,575-51,553)	55.491 (33.359-83.585)	38,338 (20,508-59,593)	58.426 (31.253-90.817)	0.12 (−0.134 to 0.354)	0.28 (0.23-0.33)
North Africa and Middle East	96,450 (56,519-144,634)	68.655 (40.231-102.953)	132,129 (77,661-204,906)	72.075 (42.363-111.774)	0.37 (−0.05 to 0.945)	0.02 (−0.1 to 0.15)
Oceania	1109 (474-1997)	41.381 (17.699-74.517)	2089 (757-3754)	41.119 (14.89-73.875)	0.884 (−0.239 to 3.482)	0.12 (0.02-0.22)
South Asia	193,523 (111,840-294,496)	44.656 (25.808-67.956)	229,609 (141,041-332,358)	45.286 (27.817-65.551)	0.186 (−0.149 to 0.808)	0.24 (0.17-0.3)
Southeast Asia	86,339 (52,647-130,261)	50.565 (30.833-76.289)	96,587 (59,802-147,585)	55.943 (34.637-85.48)	0.119 (−0.181 to 0.537)	−0.11 (−0.14 to −0.08)
Southern Latin America	8808 (4163-14,118)	59.01 (27.89-94.587)	9970 (4211-17,278)	68.783 (29.052-119.198)	0.132 (−0.483 to 1.338)	0.24 (0.2-0.28)
Southern Sub to Saharan Africa	14,793 (8831-22,184)	71.503 (42.683-107.225)	16,581 (10,047-25,437)	68.897 (41.747-105.697)	0.121 (−0.248 to 0.65)	0.14 (0.1-0.19)
Tropical Latin America	44,769 (26,147-69,219)	83.502 (48.769-129.106)	35,252 (21,287-52,134)	70.233 (42.41-103.868)	−0.213 (−0.435 to 0.139)	0.29 (0.23-0.36)
Western Europe	55,148 (33,152-81,906)	77.653 (46.68-115.331)	57,086 (32,083-87,094)	83.805 (47.1-127.857)	0.035 (−0.257 to 0.375)	−0.02 (−0.04 to 0)
Western Sub to Saharan Africa	72,292 (42,172-109,108)	82.263 (47.989-124.156)	172,799 (107,800-248,211)	80.46 (50.195-115.574)	1.39 (0.867-2.256)	−0.03 (−0.11 to 0.05)

Abbreviations: EAPC, estimated annual percentage change; SDI, Sociodemographic Index; UI, uncertainty interval.

a EAPC is expressed as 95% CIs.

#### Deaths

Over the past 30 years, the global number of deaths related to idiopathic childhood epilepsy decreased by 29.5% in 2021 compared with 1990 (18,171; 95% UI, 13,891-21,418) (Distributions are shown in Supplemental Figure 1B). Similarly, death rates dropped from 1.482 per 100,000 (95% UI, 1.01-1.778) in 1990 to 0.903 per 100,000 (95% UI, 0.69-1.065) in 2021; EAPC was −1.39 (95% CI, −1.48 to −1.3) (Supplemental Table 1, [available online at http://www.mcpiqojournal.org]). Death rates decreased across almost all age groups, with the largest decline (37.89%) in children aged 1-4 years. In 1990 and 2021, the highest number of deaths occurred in children under 1 year (6178) and those aged 10-14 years (5249), respectively. In 2021, boys had higher death rates than girls (Figure 1B).

#### DALYs

From 1990 to 2021, the number of DALYs related to childhood idiopathic epilepsy decreased by 14.90% globally, to 3,564,497 (95% UI, 2,700,944-4,753,410) in 2021. The EAPC was −0.94 (95% CI, −1 to −0.89) (the distribution is shown in Supplemental Figure 1C; Supplemental Table 2, [available online at http://www.mcpiqojournal.org]). From 1990 to 2021, the number of DALYs related to idiopathic epilepsy increased in children of almost all age groups but decreased slightly (4.05%) in children aged 10 to 14 years. The largest increase in the number of DALYs related to idiopathic epilepsy (30.31%) was observed in children under 1 year of age. The group with the largest number of DALYs related to idiopathic epilepsy in 1990 and 2021 were children aged 5 to 9 years (1,157,772) and children aged 10 to 14 years (1,145,807), respectively. DALYs rate associated with idiopathic epilepsy were generally higher in boys than in girls in 2021 (Figure 1C).

### Idiopathic Epilepsy in Children: SDI Regional Trends

#### Incidence

**Figure 2:**
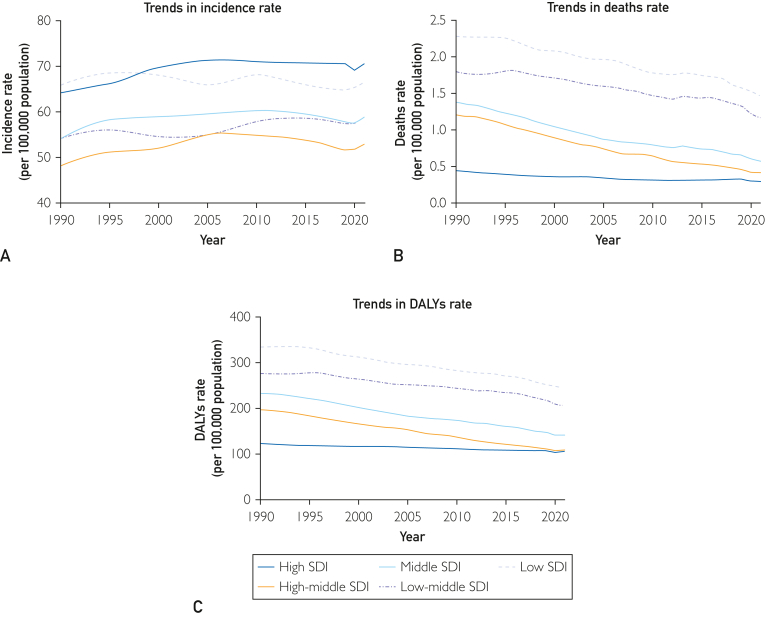
Epidemiologic trends of incidence, deaths and DALYs rates in 5 SDI regions of idiopathic epilepsy in children from 1990 to 2021. A, trends in incidence rate. B, trends in deaths rate. C, trends in DALYs rate. DALYs, disability-adjusted life years; SDI, sociodemographic inde.

In 2021, the number of children with idiopathic epilepsy in low and medium SDI areas was the highest (341,687; 95% UI, 209,540-485,226). The number of cases in low SDI areas increased by 103.1% (95% UI, 58.8%-182.5%). The largest increase in the incidence of childhood idiopathic epilepsy was seen in areas with high SDI (EAPC, 0.28; 95% CI, 0.23-0.33) (Figure 2A; Table).

#### Deaths

In all 5 SDI regions, the deaths related to idiopathic epilepsy decreased. The number of deaths related to idiopathic epilepsy increased only in the low SDI region (0.285; 95% UI, −0.062 to 0.783) in 2021, the deaths related to childhood idiopathic epilepsy was the highest in areas with low SDI (1.459; 95% UI, 1.067-1.804). The lowest values were found in high SDI area (0.298; 95% UI, 0.274-0.318). The EAPC was lowest in the middle-high SDI region (−3.45; 95% UI, −3.52 to −3.37) (Figure 2B; Supplemental Table 1).

#### DALYs

In 2021, the number of epilepsy-related DALYs in low-to-moderate SDI areas was the highest (1,199,709; 95% UI, 892,917-1,592,918), which declined by only 8.1% from 1990 to 2021. The largest increase of DALY was seen in low SDI areas, with an increase of 46.8%. The greatest decrease in the number of DALYs associated with idiopathic epilepsy (53.4%) was observed in the middle-high SDI region (Figure 2C; Supplemental Table 2).

### Idiopathic Epilepsy in Children: Geographic Regional Trends

#### Incidence

**Figure 3:**
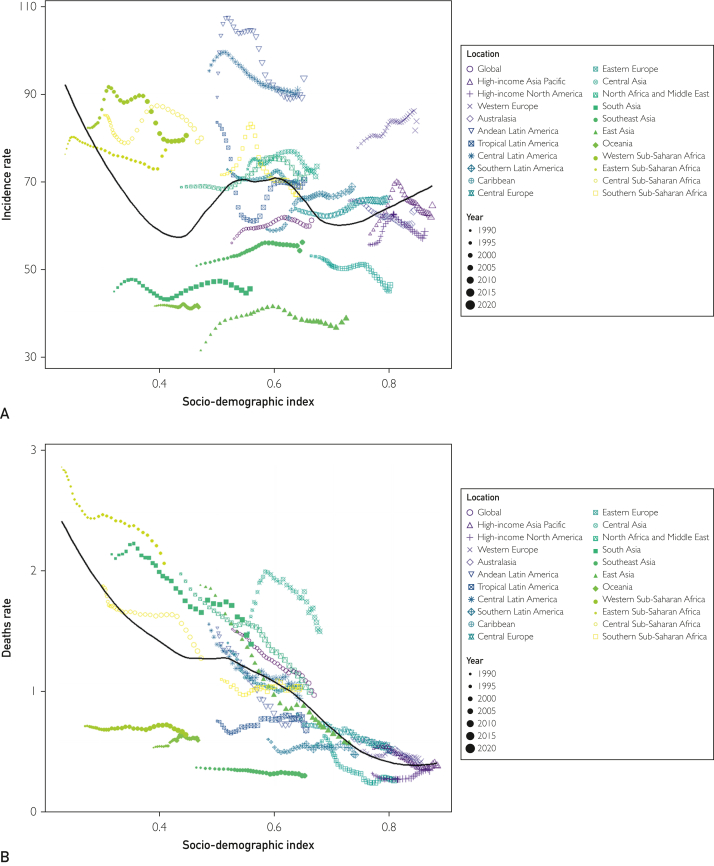

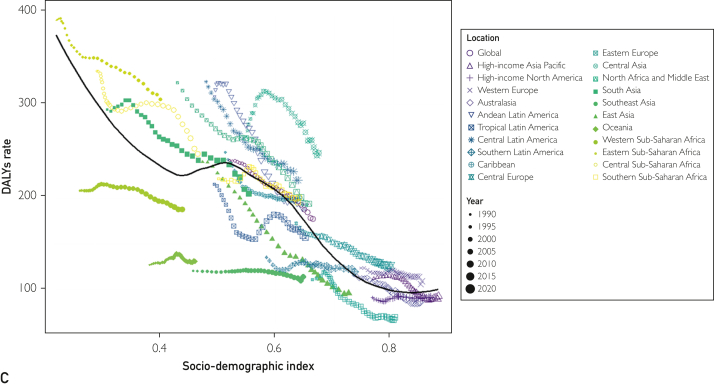
Incidence, deaths, and DALYs rates for idiopathic epilepsy in children from 1990 to 2021. A, incidence rate. B, deaths rate. C, DALYs rate. DALYs, disability-adjusted life years.

Among the 21 geographic regions, the incidence of childhood idiopathic epilepsy was highest in Andean Latin America (89.312; 95% UI, 0.414-143.492). In contrast, East Asian children had the lowest incidence of idiopathic epilepsy (31.189; 95% UI, 18.206-46.628). From 1990 to 2021, tropical Latin America had the greatest increase in the incidence of childhood idiopathic epilepsy (EAPC, 0.29; 95% CI, 0.23-0.36), with the largest reductions observed in Andean Latin America (EAPC, −0.53; 95% CI, −0.66 to −0.39) (Table). In 2021, Andean Latin America had the highest incidence of pediatric idiopathic epilepsy (SDI, 0.65), whereas Eastern Asia had the lowest incidence (SDI, 0.73) (Figure 3A).

#### Deaths

In 2021, eastern sub-Saharan Africa had the highest childhood idiopathic epileptic-related deaths (2.06; 95% UI, 1.565-2.589). The smallest reduction In childhood idiopathic epileptic-related deaths was observed in western sub-Saharan Africa (EAPC, −0.02; 95%CI, −0.15 to 0.11), with the largest decline observed in East Asia (EAPC, −4.65; 95%CI, − 5.3 to − 4). In 2021, eastern sub-Saharan Africa had the highest deaths associated with childhood idiopathic epilepsy (SDI, 0.41), whereas Eastern Europe had the lowest deaths (SDI, 0.80) (Figure 3B; Supplemental Table 1).

#### DALYs

In 2021, eastern sub-Saharan Africa had the highest DALYs rate (306.532; 95% UI, 228.148-407.969); Eastern Europe had the lowest DALYs rate (70.558; 95% UI, 42.518-116.767). The smallest decline in DALYs rates from 1990 to 2021 was observed in Southern Latin America (EAPC, −0.15; 95% CI, −0.24 to −0.05). The largest decline was observed in East Asia (EAPC, −3.18; 95% CI, −3.26 to −3.1) (Figure 3C; Supplemental Table 2).

### Idiopathic Epilepsy in Children: National Trends

#### Incidence

**Figure 4:**
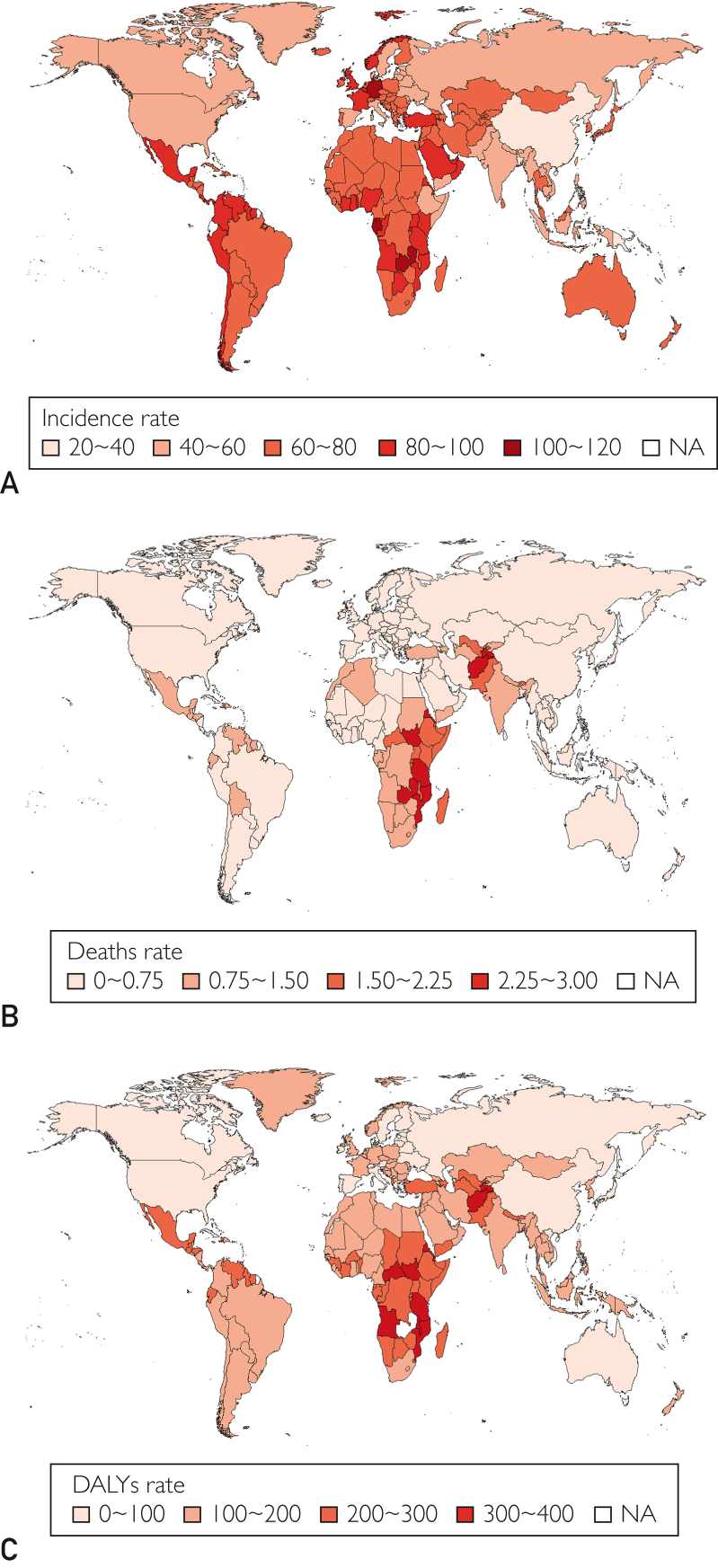
The incidence, deaths and DALYs rates of idiopathic epilepsy in children in 204 countries and territories. A, disease burden of incidence rate. B, disease burden of deaths rate. C, disease burden of DALYs rate. DALYs, disability-adjusted life years.

Ecuador had the highest incidence of childhood idiopathic epilepsy among 204 countries in 2021 (120.091; 95% UI, 37.039-214.83) (Figure 4A; Supplemental Figure 3A [available online at http://www.mcpiqojournal.org]; Supplemental Table 3, [available online at http://www.mcpiqojournal.org]). Equatorial Guinea (EAPC, 1.43; 95% CI, 1.19-1.67) had the largest increase in the incidence of idiopathic epilepsy in children. Burundi (EAPC, −1.39; 95% CI, −1.62 to −1.16) (Supplemental Figure 2A; Supplemental Table 3). In 2021, Ecuador (SDI, 0.66) had the highest incidence of childhood idiopathic epilepsy, while the Democratic People's Republic of Korea (SDI, 0.57) had the lowest.

#### Deaths

In 2021, Tajikistan (2.766; 95% UI, 1.839-4.078) had the highest deaths related to childhood idiopathic epilepsy. Vietnam (0.03; 95% UI, 0.01-0.113) had the lowest deaths (Figure 4B; Supplemental Figure 3B; Supplemental Table 4, [available online at http://www.mcpiqojournal.org]). Northern Mariana Islands (EAPC, 3.18; 95% CI, 2.37-3.99). Estonia (EAPC, −6.48; 95% CI, −7.32 to −5.63) (Supplemental Figure 2B; Supplemental Table 4). In 2021, Tajikistan (SDI, 0.54) had the highest deaths rate related to childhood idiopathic epilepsy, whereas Vietnam (SDI, 0.63) had the lowest.

#### DALYs

In 2021, Zambia had the highest DALY rate related to childhood idiopathic epilepsy (403.326; 95% UI, 235.243-637.115) (Figure 4C; Supplemental Figure 3C; Supplemental Table 5, [available online at http://www.mcpiqojournal.org]). Lesotho (EAPC, 0.98; 95% CI, 0.84-1.11). China (EAPC, −3.24; 95% CI, −3.32 to −3.16) had the greatest reduction in the DALY rate (Supplemental Figure 2C; Supplemental Table 5). Zambia (SDI, 0.51) had the highest DALY rate related to childhood idiopathic epilepsy. Sweden had the lowest rate (SDI, 0.89).

### Factors Influencing EAPCs

The incidence rate, deaths rate, and DALYs rate in EAPC showed significant differences compared with those in 1990. These figures also varied significantly in relation to the 2021 HDI. The 1990 incidence serves as the baseline for the disease pool, while the HDI can be seen as a measure of health care quality. A positive relationship was identified between the EAPC of incidence and HDI (Pearson r=0.359; *P*<.001). In contrast, the EAPC of the DALYs rate showed an inverse correlation with HDI (Pearson r=−0.053; *P*<.001). In addition, the EAPC of deaths was negatively correlated with SDI (Pearson r=−0.215; *P*<.001) (Supplemental Figure 4).

## Discussion

This research offers important perspectives on the global, regional, and national impact of childhood idiopathic epilepsy, forming a foundation for targeted health care interventions and policy reforms. The analysis of over 3 decades of data highlights progress in reducing epilepsy-related deaths, while also exposing ongoing disparities, particularly in low SDI regions. Understanding these trends is essential, as childhood epilepsy can have long-term consequences on cognitive development, educational outcomes, and overall quality of life. The findings emphasize the need to improve health care access, diagnostic tools, and early intervention strategies to mitigate the condition’s impact on affected children and their families.^5^

From 1990 to 2021, the global incidence of childhood idiopathic epilepsy rose notably, with a 26.3% increase in new cases, particularly affecting children in low SDI regions. Despite this rise in incidence, epilepsy-related deaths have decreased by 29.5%, largely due to advancements in health care in high SDI regions. However, deaths remain disproportionately high in low-resource areas, where access to health care and treatment is limited. The burden of DALYs attributed to epilepsy has also reduced, although the smallest reductions were observed in low SDI regions, underscoring persistent health care inequities. High SDI regions experienced the most substantial decreases in both deaths and DALYs, whereas low SDI areas continued to shoulder a significant burden of epilepsy. This difference underscores the critical necessity for targeted health initiatives and allocation of resources in these neglected areas. Although some regions have made notable progress, the overall global trend underscores the need for more equitable health care advancements to close the gap in childhood epilepsy outcomes worldwide.

This study aligns with recent findings emphasizing the need for systematic interventions to manage epilepsy, particularly in resource-limited settings. Standardized classification systems for seizures and epilepsy, such as those developed by the International League Against Epilepsy, provide a foundation for understanding epilepsy types and aiding diagnosis, treatment, and disease burden tracking.^17^^,^^18^ Emerging genetic research has identified mutations in genes such as SCN1A and PCDH19, which are associated with specific forms of epilepsy and may offer early diagnostic markers for high-risk children.^19^^,^^20^ Such genetic insight could guide the development of targeted screening programs, especially in regions with high prevalence but limited access to advanced diagnostic methods.^1^ Gaps remain in understanding how specific social determinants influence disease progression and outcomes in children. For example, the association between maternal health, early childhood nutrition, and seizures has not been fully explored and could be a key area of intervention in areas with high prevalence.^6^^,^^21^ These findings are consistent with GBD studies, which consistently point to a higher burden of epilepsy in areas with lower SDI due to healthcare disparities.^22^ Although improvements in deaths globally reflect advances in medical technology, deaths in low SDI regions are still significantly higher than in other regions, indicating that the uneven distribution of medical resources remains one of the global health challenges. For future research, the application of artificial intelligence and machine learning in epilepsy prediction models has the potential to transform early diagnosis and personalized treatment planning. By improving diagnostic capabilities, optimizing treatment options, predicting exacerbations, optimizing resource allocation, and supporting community health interventions, machine learning can help bridge the health care resource gap and improve patient outcomes. Machine learning algorithms can analyze a combination of genetic, demographic characteristics, and clinical data to predict disease progression, providing cost-effective solutions for resource-constrained health care systems.^23^^,^^24^ In addition, clinical trials of alternative treatment options and community health interventions for a variety of socioeconomic conditions could provide valuable data for improving outcomes in resource-poor settings.^25^

In conclusion, this study emphasizes the importance of comprehensive interventions addressing genetic, socioeconomic, and health care access factors to alleviate the global burden of childhood idiopathic epilepsy. Enhancing diagnostic capabilities, particularly through genetic screening in resource-limited settings, could substantially improve early intervention strategies. In addition, interdisciplinary research on neurodevelopmental outcomes is crucial for the development of effective prevention approaches.

### Limitations

This study has several limitations. First, the GBD database typically aggregates data on broad epilepsy categories, lacking detailed distinctions between subtypes of childhood idiopathic epilepsy, making it difficult to analyze the epidemiological characteristics of specific types such as absence epilepsy or myoclonic epilepsy. Second, the GBD database primarily focuses on disease burden and lacks detailed data on clinical progression, treatment responses, and long-term outcomes in patients with idiopathic epilepsy, and data on risk factors for childhood idiopathic epilepsy.^26^ Some forms of pediatric idiopathic epilepsy exhibit favorable prognoses and a tendency toward spontaneous remission; these details are crucial for accurately interpreting the actual burden.

## Conclusion

This study found a 26.3% increase in childhood idiopathic epilepsy cases globally from 1990 to 2021, particularly affecting low SDI regions where access to health care is still limited. Although epileptic-related deaths and DALYs declined overall, the declines were smaller in low SDI regions, highlighting persistent health disparities. The global decline in deaths is mainly due to the improvement of medical resources and the popularization of anti-epilepsy treatment in regions with high SDI, whereas the high burden in regions with low SDI still needs to be concerned. These results highlight the need for targeted interventions to bridge health care gaps in low-resource settings and ensure a reduction in the disease burden for children affected by epilepsy worldwide.

## Potential Competing Interests

The authors report no competing interests.

## Ethics Statement

This study was reviewed by Mayo Clinic IRB000-000000 and deemed exempt. The study data areretrospective and de-identified by CMS; therefore, patients areunable to be identified and patient consent was not obtained.
