# Supplementary material for: Epidemiological Study of Childhood Idiopathic Epilepsy from 1990 to 2021 at Global, Regional, and National Scales
Source: Mayo Clin Proc Innov Qual Outcomes. 2025 Jun 25;9(4):100641. doi: 10.1016/j.mayocpiqo.2025.100641 (PMC12348364; doi:10.1016/j.mayocpiqo.2025.100641)
Supplement: Supplemental Material [file mmc1.pdf]

Supplementary Figure 1. Incident, Death, and DALYs Cases of Idiopathic Epilepsy in Children in 204 Countries and Territories

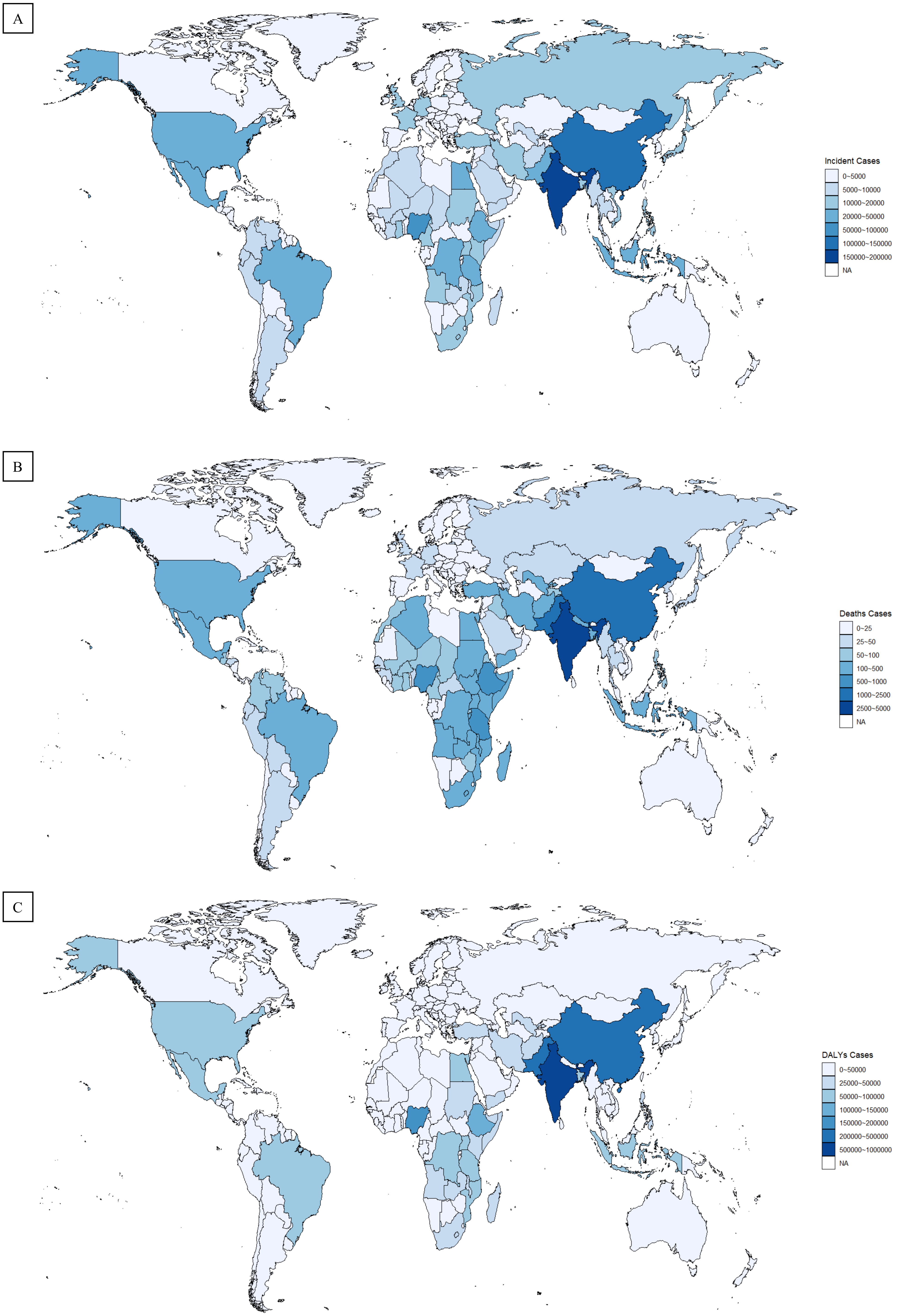

A, Incident cases. B, Death cases. C, DALYs cases. DALYs=disability-adjusted life-years.

Supplementary Figure 2. The National Burden of Idiopathic Epilepsy in Children in 204 Countries and Territories

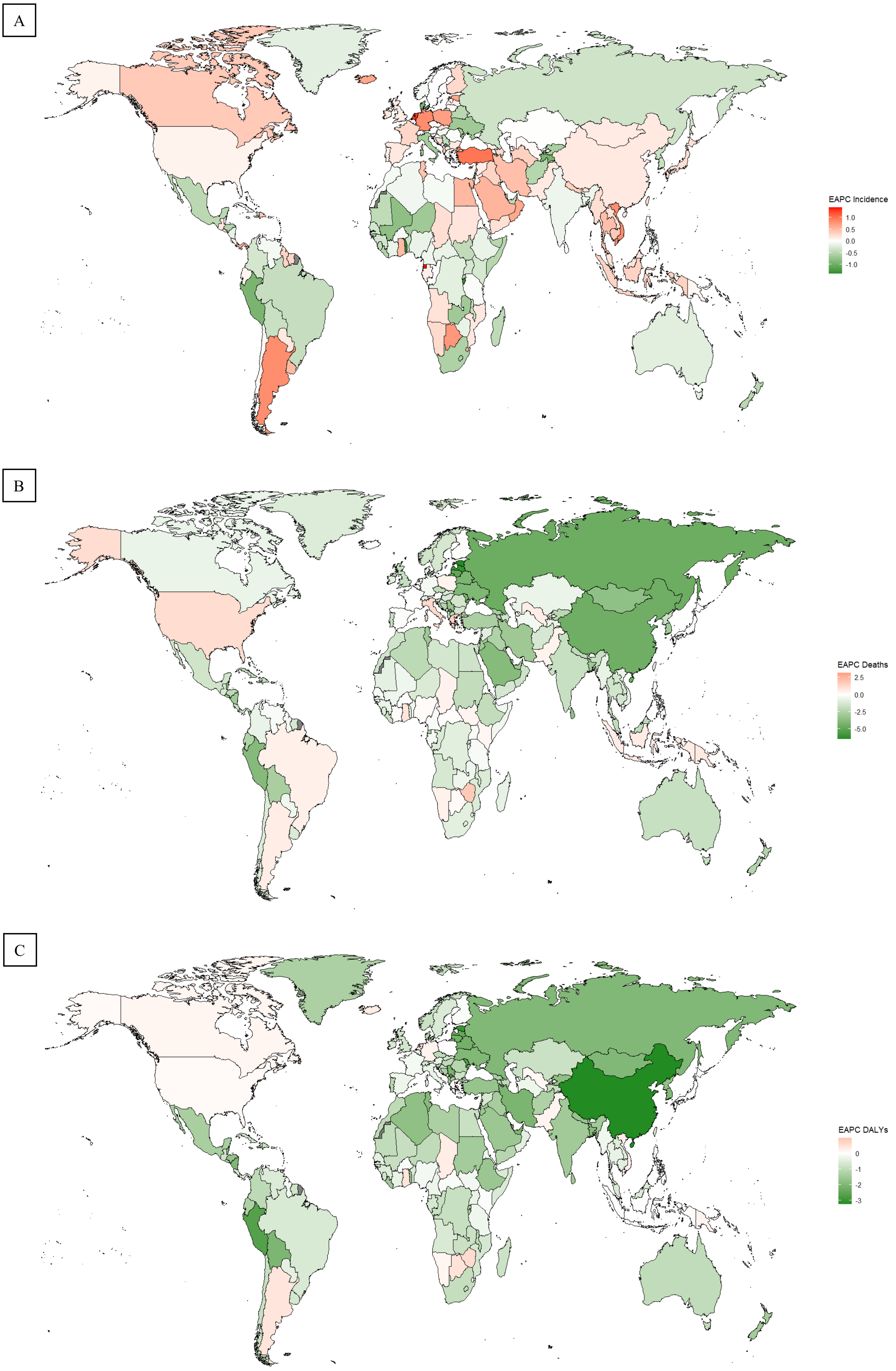

A, EAPC for incidence rate. B, EAPC for deaths rate. C, EAPC for DALYs rate. DALYs=disability-adjusted life-years. EAPC=estimated annual percentage change.

Supplementary Figure 3. Incidence, Deaths, and DALYs Rates of Idiopathic Epilepsy in Children in 204 Countries by SDI in 2021

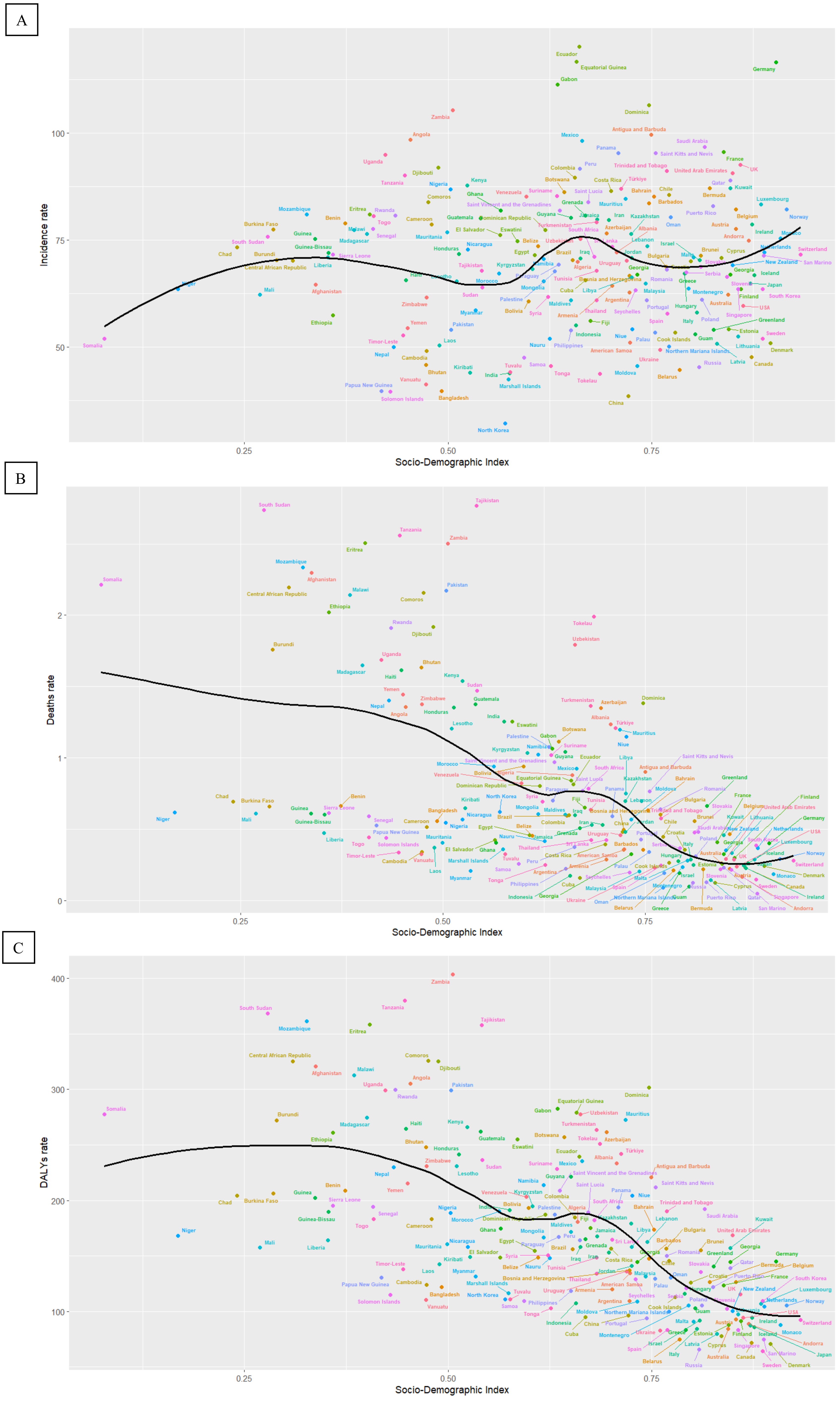

A, Incidence rate. B, Deaths rate. C, DALYs rate. DALYs=disability-adjusted life-years; SDI= sociodemographic index.

Supplementary Figure 4. The Correlation between EAPC and Incidence Rate, Deaths Rate, and DALYs Rate of Idiopathic Epilepsy in Children in 1990 and HDI in 2021

A

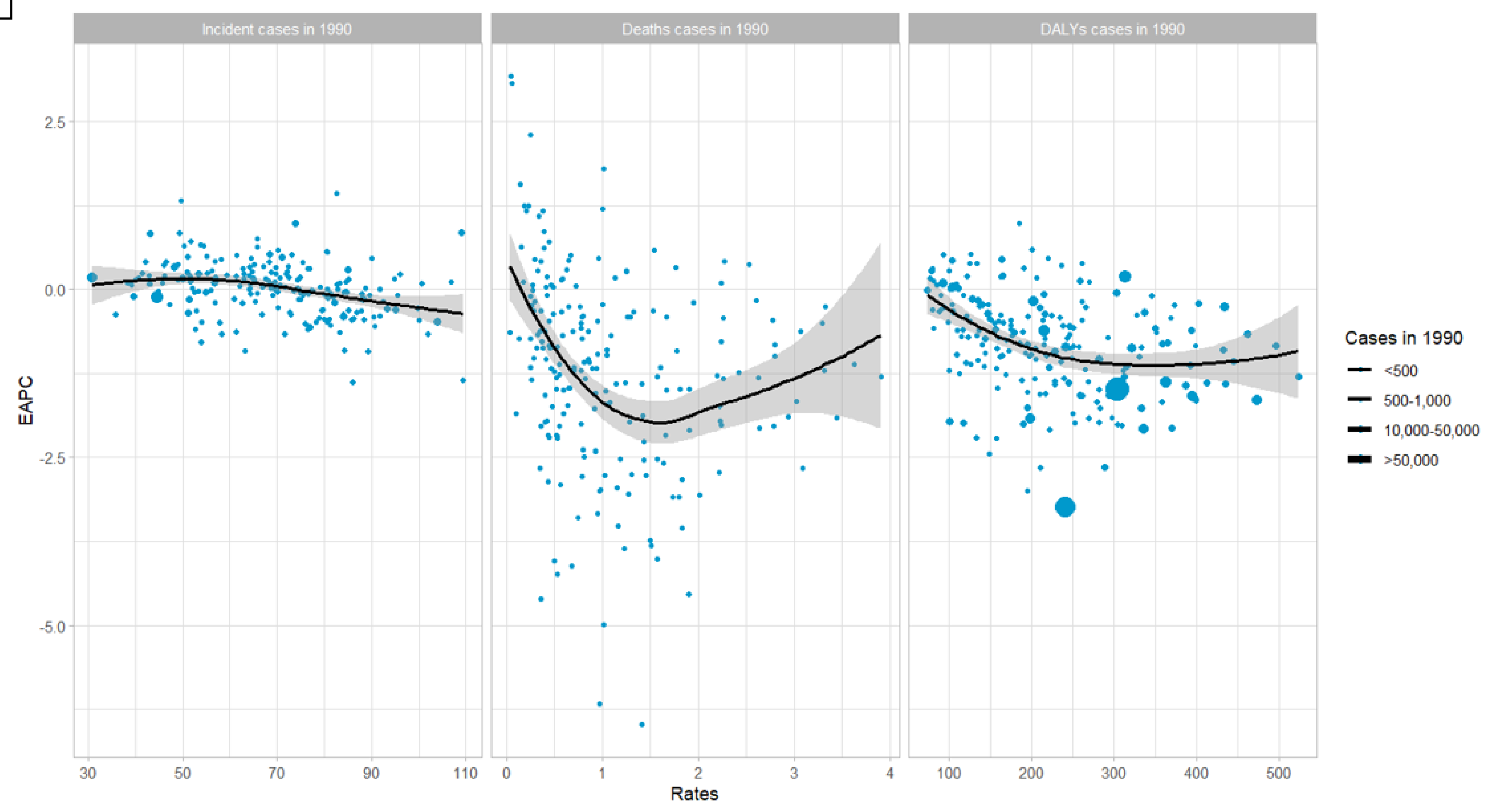

B

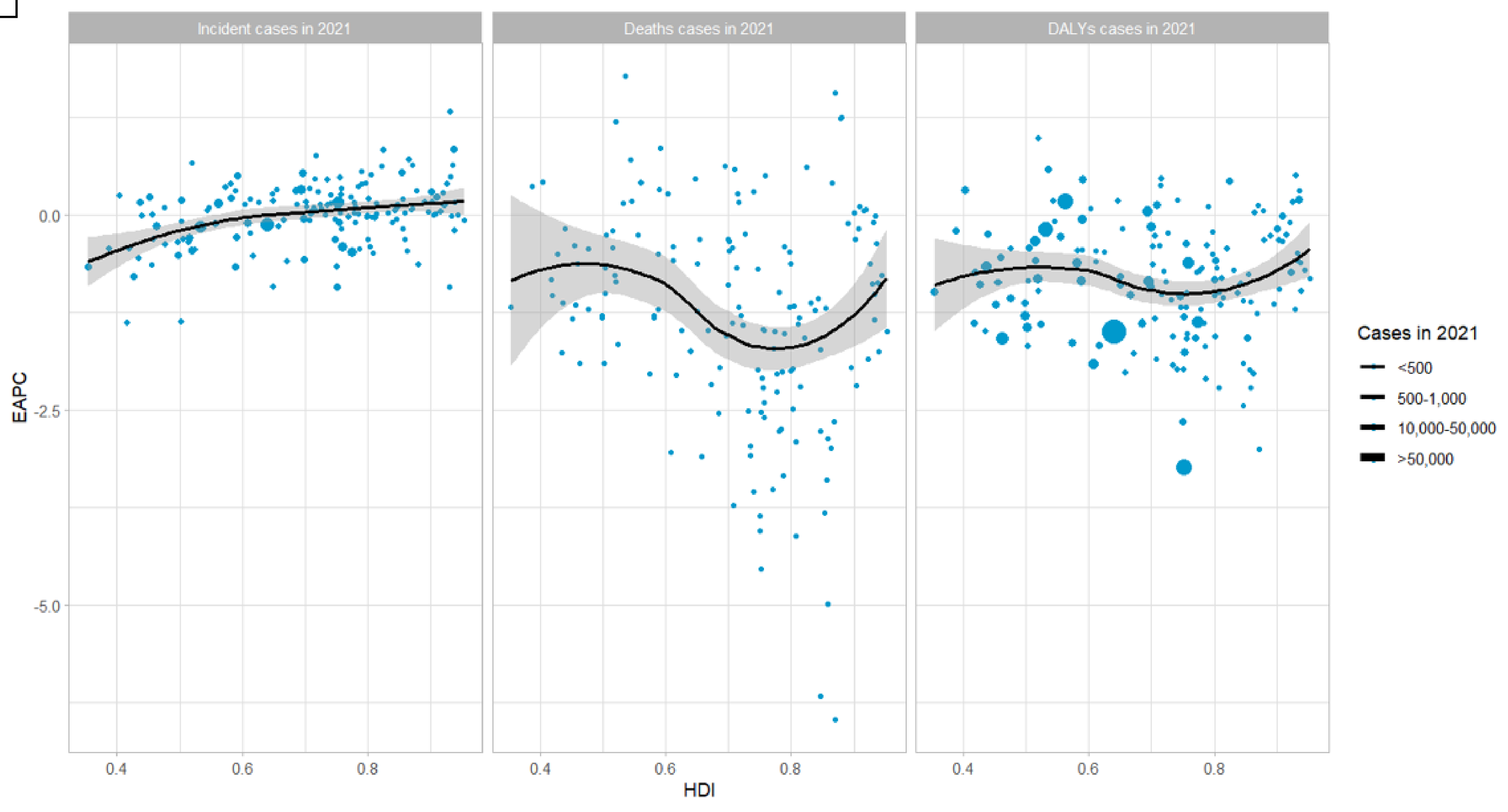

A, The correlation between EAPC and incidence rate, deaths rate, and DALYs rate of idiopathic epilepsy in Children in 1990. B, The correlation between EAPC and HDI in 2021. DALYs=disability-adjusted life-years; EAPC=estimated annual percentage change; HDI= human development index.

Supplementary Table 1. Deaths of Idiopathic Epilepsy in Children Between 1990 and 2021 at the Global and Regional Level

Location	Rate per 100 000 (95%UI)					
	1990		2021		1990-2021	
	**Deaths cases**	**Deaths rate**	**Deaths cases**	**Deaths rate**	**Cases change**	**EAPC[a]**
Global	25768 (17567 to 30914)	1.482 (1.01 to 1.778)	18171 (13891 to 21418)	0.903 (0.69 to 1.065)	-0.295 (-0.433 to -0.1)	-1.39 (-1.48 to -1.3)
SDI						
High	819 (763 to 902)	0.441 (0.411 to 0.485)	513 (473 to 548)	0.298 (0.274 to 0.318)	-0.373 (-0.444 to -0.317)	-1.12 (-1.26 to -0.98)
High middle	3299 (2564 to 3895)	1.206 (0.937 to 1.424)	957 (827 to 1136)	0.414 (0.358 to 0.492)	-0.71 (-0.766 to -0.622)	-3.45 (-3.52 to -3.37)
Middle SDI	7945 (5566 to 9203)	1.376 (0.964 to 1.594)	3237 (2571 to 3726)	0.571 (0.454 to 0.657)	-0.593 (-0.665 to -0.476)	-2.63 (-2.75 to -2.51)
Low middle	8467 (5016 to 10600)	1.793 (1.062 to 2.245)	6740 (4812 to 8160)	1.162 (0.83 to 1.407)	-0.204 (-0.374 to 0.08)	-1.2 (-1.33 to -1.07)
Low SDI	5225 (3421 to 6759)	2.283 (1.494 to 2.952)	6712 (4909 to 8302)	1.459 (1.067 to 1.804)	0.285 (-0.062 to 0.783)	-1.32 (-1.4 to -1.24)
Regions						
Andean Latin America	222 (164 to 264)	1.496 (1.103 to 1.781)	110 (88 to 137)	0.606 (0.485 to 0.758)	-0.507 (-0.628 to -0.274)	-3.01 (-3.25 to -2.78)
Australasia	24 (22 to 26)	0.529 (0.49 to 0.575)	16 (14 to 19)	0.277 (0.24 to 0.326)	-0.345 (-0.436 to -0.23)	-1.9 (-2.13 to -1.68)
Caribbean	155 (102 to 223)	1.362 (0.895 to 1.953)	111 (78 to 163)	0.968 (0.676 to 1.417)	-0.283 (-0.441 to -0.09)	-0.93 (-1.15 to -0.7)
Central Asia	363 (321 to 403)	1.454 (1.286 to 1.612)	405 (322 to 495)	1.464 (1.164 to 1.79)	0.115 (-0.095 to 0.399)	-0.22 (-0.57 to 0.13)
Central Europe	217 (197 to 234)	0.736 (0.668 to 0.793)	75 (62 to 87)	0.425 (0.348 to 0.49)	-0.653 (-0.719 to -0.596)	-1.59 (-1.7 to -1.48)
Central Latin America	983 (918 to 1052)	1.527 (1.426 to 1.635)	556 (443 to 701)	0.876 (0.698 to 1.103)	-0.434 (-0.555 to -0.285)	-1.27 (-1.46 to -1.09)
Central Sub to Saharan Africa	471 (335 to 692)	1.862 (1.323 to 2.736)	722 (511 to 968)	1.23 (0.871 to 1.649)	0.533 (0.011 to 1.113)	-0.93 (-1.09 to -0.76)
East Asia	6161 (4185 to 7427)	1.868 (1.269 to 2.252)	1285 (1056 to 1720)	0.48 (0.395 to 0.643)	-0.791 (-0.842 to -0.667)	-4.46 (-4.64 to -4.29)
Eastern Europe	236 (224 to 249)	0.459 (0.436 to 0.485)	56 (49 to 62)	0.157 (0.138 to 0.174)	-0.764 (-0.791 to -0.74)	-4.65 (-5.3 to -4)
Eastern Sub to Saharan Africa	2628 (1902 to 3318)	2.902 (2.1 to 3.664)	3675 (2792 to 4619)	2.06 (1.565 to 2.589)	0.398 (0.015 to 0.965)	-0.88 (-0.97 to -0.79)
High to income Asia Pacific	154 (132 to 194)	0.438 (0.375 to 0.552)	64 (57 to 69)	0.284 (0.254 to 0.309)	-0.587 (-0.687 to -0.507)	-1.89 (-2.2 to -1.57)
High to income North America	136 (132 to 140)	0.22 (0.214 to 0.227)	159 (147 to 172)	0.242 (0.223 to 0.262)	0.171 (0.075 to 0.275)	0.96 (0.48 to 1.44)
North Africa and Middle East	2762 (1703 to 3530)	1.966 (1.212 to 2.513)	1712 (1226 to 2044)	0.934 (0.669 to 1.115)	-0.38 (-0.52 to -0.182)	-1.98 (-2.16 to -1.8)
Oceania	12 (8 to 16)	0.446 (0.292 to 0.602)	26 (17 to 36)	0.512 (0.343 to 0.706)	1.177 (0.613 to 2.004)	0.6 (0.43 to 0.77)
South Asia	9269 (5109 to 11969)	2.139 (1.179 to 2.762)	6846 (4809 to 8554)	1.35 (0.948 to 1.687)	-0.261 (-0.445 to 0.067)	-1.35 (-1.53 to -1.18)
Southeast Asia	465 (341 to 556)	0.272 (0.2 to 0.326)	342 (221 to 436)	0.198 (0.128 to 0.253)	-0.263 (-0.417 to -0.091)	-0.87 (-0.96 to -0.79)
Southern Latin America	77 (72 to 82)	0.518 (0.485 to 0.549)	56 (47 to 65)	0.383 (0.326 to 0.447)	-0.281 (-0.392 to -0.153)	-0.12 (-0.37 to 0.12)
Southern Sub to Saharan Africa	216 (165 to 256)	1.043 (0.796 to 1.237)	235 (186 to 297)	0.976 (0.774 to 1.236)	0.088 (-0.149 to 0.439)	-0.02 (-0.15 to 0.11)
Tropical Latin America	363 (310 to 433)	0.677 (0.578 to 0.807)	301 (234 to 364)	0.6 (0.467 to 0.726)	-0.17 (-0.406 to 0.054)	0.47 (0.24 to 0.71)
Western Europe	295 (286 to 304)	0.416 (0.403 to 0.428)	221 (198 to 245)	0.325 (0.29 to 0.36)	-0.252 (-0.336 to -0.172)	-0.47 (-0.67 to -0.26)
Western Sub to Saharan Africa	557 (390 to 745)	0.634 (0.444 to 0.847)	1199 (737 to 1531)	0.558 (0.343 to 0.713)	1.153 (0.469 to 1.863)	-0.08 (-0.19 to 0.03)

Abbreviations: EAPC, estimated annual percentage change; SDI, Sociodemographic Index; UI, uncertainty interval.

[a] EAPC is expressed as 95% CIs.

Supplementary Table 2. Dalys of Idiopathic Epilepsy in Children Between 1990 and 2021 at the Global and Regional Level

Location	Rate per 100 000 (95%UI)					
	1990		2021		1990-2021	
	DALYs cases	DALYs rate	DALYs cases	DALYs rate	Cases change	EAPC[a]
Global	4188140 (3112385 to 5405105)	240.815 (178.96 to 310.79)	3564497 (2700944 to 4753410)	177.174 (134.251 to 236.269)	-0.149 (-0.279 to 0.014)	-0.94 (-1 to -0.89)
SDI						
High SDI	228475 (155099 to 344204)	122.963 (83.473 to 185.248)	182867 (110650 to 305006)	105.987 (64.131 to 176.777)	-0.2 (-0.351 to -0.042)	-0.48 (-0.51 to -0.45)
High middle	539691 (416585 to 689060)	197.239 (152.248 to 251.828)	251502 (171033 to 376966)	108.927 (74.076 to 163.266)	-0.534 (-0.651 to -0.373)	-2.02 (-2.07 to -1.97)
Middle SDI	1345361 (994554 to 1727491)	233.078 (172.303 to 299.281)	802489 (577972 to 1159409)	141.567 (101.96 to 204.531)	-0.404 (-0.517 to -0.254)	-1.61 (-1.66 to -1.56)
Low middle	1305333 (921270 to 1726607)	276.488 (195.138 to 365.72)	1199709 (892917 to 1592918)	206.903 (153.993 to 274.716)	-0.081 (-0.264 to 0.19)	-0.87 (-0.95 to -0.79)
Low SDI	766403 (540704 to 1065347)	334.801 (236.204 to 465.393)	1125372 (827951 to 1507061)	244.526 (179.901 to 327.461)	0.468 (0.145 to 0.85)	-1.01 (-1.06 to -0.96)
Regions						
Andean Latin America	46021 (27628 to 68380)	309.863 (186.021 to 460.407)	34544 (19626 to 54820)	190.905 (108.463 to 302.959)	-0.249 (-0.594 to 0.357)	-2.08 (-2.26 to -1.91)
Australasia	5470 (3131 to 9556)	119.287 (68.268 to 208.373)	5002 (2309 to 10005)	87.278 (40.285 to 174.571)	-0.086 (-0.557 to 0.855)	-1.09 (-1.16 to -1.03)
Caribbean	28439 (18693 to 41023)	249.19 (163.799 to 359.456)	22973 (15139 to 32972)	199.678 (131.581 to 286.585)	-0.192 (-0.445 to 0.125)	-0.62 (-0.75 to -0.5)
Central Asia	66486 (48706 to 88766)	266.036 (194.893 to 355.188)	68749 (50380 to 93874)	248.41 (182.038 to 339.191)	0.034 (-0.216 to 0.392)	-0.48 (-0.72 to -0.24)
Central Europe	50796 (35422 to 71786)	172.285 (120.143 to 243.478)	22417 (14552 to 34102)	126.639 (82.207 to 192.652)	-0.559 (-0.674 to -0.423)	-1.01 (-1.06 to -0.97)
Central Latin America	209810 (156507 to 283623)	325.887 (243.095 to 440.537)	139103 (94119 to 202419)	219.112 (148.255 to 318.846)	-0.337 (-0.486 to -0.165)	-1.18 (-1.26 to -1.1)
Central Sub to Saharan Africa	85364 (49155 to 133552)	337.427 (194.298 to 527.9)	145072 (86378 to 224226)	247.219 (147.198 to 382.106)	0.699 (0.044 to 1.886)	-0.69 (-0.83 to -0.56)
East Asia	788642 (593632 to 964866)	239.103 (179.979 to 292.531)	258439 (182603 to 376350)	96.666 (68.3 to 140.769)	-0.672 (-0.764 to -0.536)	-3.18 (-3.26 to -3.1)
Eastern Europe	57275 (40999 to 84336)	111.296 (79.668 to 163.881)	25009 (15070 to 41387)	70.558 (42.518 to 116.767)	-0.563 (-0.677 to -0.435)	-2.11 (-2.3 to -1.92)
Eastern Sub to Saharan Africa	355500 (262055 to 462575)	392.511 (289.338 to 510.733)	546949 (407088 to 727945)	306.532 (228.148 to 407.969)	0.539 (0.169 to 0.96)	-0.78 (-0.82 to -0.73)
High to income Asia Pacific	39632 (25189 to 60254)	112.591 (71.56 to 171.178)	20980 (12035 to 36387)	93.554 (53.666 to 162.256)	-0.471 (-0.65 to -0.209)	-1 (-1.12 to -0.87)
High to income North America	56803 (33078 to 89450)	92.096 (53.631 to 145.028)	60556 (35747 to 101167)	92.284 (54.477 to 154.173)	0.066 (-0.173 to 0.364)	0.1 (0.04 to 0.16)
North Africa and Middle East	456167 (319339 to 612165)	324.708 (227.311 to 435.75)	355021 (251380 to 514904)	193.66 (137.125 to 280.874)	-0.222 (-0.403 to 0.042)	-1.39 (-1.53 to -1.26)
Oceania	3415 (1888 to 5720)	127.418 (70.443 to 213.45)	6659 (3541 to 11193)	131.062 (69.702 to 220.3)	0.95 (0.01 to 2.675)	0.08 (-0.02 to 0.18)
South Asia	1280287 (863310 to 1691068)	295.431 (199.212 to 390.221)	1034317 (766837 to 1348291)	203.999 (151.243 to 265.924)	-0.192 (-0.379 to 0.099)	-1.17 (-1.28 to -1.06)
Southeast Asia	206577 (133833 to 305998)	120.984 (78.381 to 179.211)	197503 (126719 to 312587)	114.393 (73.395 to 181.049)	-0.044 (-0.281 to 0.244)	-0.18 (-0.25 to -0.12)
Southern Latin America	20226 (11630 to 31372)	135.502 (77.918 to 210.178)	17371 (9486 to 30024)	119.836 (65.442 to 207.125)	-0.141 (-0.534 to 0.593)	-0.15 (-0.24 to -0.05)
Southern Sub to Saharan Africa	45646 (33224 to 63376)	220.624 (160.587 to 306.321)	48276 (33514 to 67955)	200.601 (139.259 to 282.373)	0.058 (-0.188 to 0.418)	-0.41 (-0.52 to -0.29)
Tropical Latin America	115119 (77392 to 173763)	214.719 (144.35 to 324.099)	78602 (53906 to 116742)	156.599 (107.397 to 232.587)	-0.317 (-0.498 to -0.063)	-0.61 (-0.9 to -0.32)
Western Europe	88021 (56635 to 139815)	123.942 (79.747 to 196.872)	74643 (43971 to 128996)	109.578 (64.551 to 189.37)	-0.152 (-0.391 to 0.115)	-0.28 (-0.35 to -0.21)
Western Sub to Saharan Africa	182445 (113676 to 277989)	207.608 (129.354 to 316.329)	402312 (261094 to 583550)	187.328 (121.572 to 271.717)	1.205 (0.742 to 1.818)	-0.32 (-0.43 to -0.22)

Abbreviations: EAPC, estimated annual percentage change; SDI, Sociodemographic Index; UI, uncertainty interval.

[a] EAPC is expressed as 95% Cls.

Supplementary Table 3. Incidence of Childhood Idiopathic Epilepsy at the National Level

Location	1990		2021		1990-2021	
	Incident cases	Incidence rate	Incident cases	Incidence rate	Cases change	EAPC[a]
Afghanistan	3278 (488 to 6780)	76.09 (11.33 to 157.37)	9163 (2090 to 18096)	64.527 (14.72 to 127.43)	1.795 (-0.311 to 16.051)	-0.51 (-0.61 to -0.41)
Albania	727 (194 to 1320)	65.05 (17.39 to 118.129)	320 (80 to 581)	72.15 (18.08 to 130.9)	-0.56 (-0.894 to 0.533)	0.36 (0.25 to 0.48)
Algeria	8120 (1937 to 15603)	75.71 (18.06 to 145.48)	9287 (2247 to 16933)	69.82 (16.89 to 127.3)	0.144 (-0.742 to 3.27)	-0.1 (-0.15 to -0.04)
American Samoa	10 (3 to 19)	51.49 (15.143 to 97.32)	7 (2 to 13)	50.941 (12.758 to 93.03)	-0.262 (-0.796 to 1.667)	0.12 (0.01 to 0.23)
Andorra	8 (3 to 13)	79.79 (26.67 to 132.9)	8 (2 to 13)	74.81 (20.41 to 130.65)	0.003 (-0.633 to 1.611)	-0.31 (-0.35 to -0.27)
Angola	4537 (848 to 9632)	96.23 (17.99 to 204.289)	15001 (3792 to 27094)	98.39 (24.88 to 177.719)	2.306 (-0.282 to 16.317)	0.22 (0.14 to 0.29)
Antigua and Barbuda	17 (4 to 33)	95.53 (24.303 to 180.12)	17 (5 to 30)	99.51 (27.2 to 176.32)	-0.032 (-0.719 to 3.027)	-0.1 (-0.2 to 0.01)
Argentina	5016 (1335 to 8563)	49.49 (13.169 to 84.49)	6383 (1501 to 11588)	62.68 (14.744 to 113.8)	0.273 (-0.69 to 3.716)	0.83 (0.61 to 1.04)
Armenia	671 (179 to 1259)	64.32 (17.14 to 120.663)	381 (120 to 698)	64.26 (20.31 to 117.824)	-0.433 (-0.833 to 1.186)	0.03 (-0.1 to 0.15)
Australia	2379 (684 to 4199)	62.842 (18.059 to 110.91)	2953 (840 to 5187)	62.18 (17.68 to 109.21)	0.241 (-0.648 to 3.21)	-0.21 (-0.29 to -0.12)
Austria	1001 (278 to 1781)	74.255 (20.61 to 132.092)	1006 (269 to 1727)	77.59 (20.77 to 133.18)	0.005 (-0.625 to 3.056)	0.01 (-0.03 to 0.05)
Azerbaijan	1750 (393 to 3118)	72.13 (16.19 to 128.463)	1807 (495 to 3272)	76.53 (20.98 to 138.6)	0.032 (-0.727 to 2.928)	0.27 (0.08 to 0.45)
Bahamas	68 (18 to 122)	84.55 (22.86 to 151.49)	69 (21 to 120)	84.91 (26.38 to 148.1)	0.011 (-0.69 to 2.346)	-0.02 (-0.1 to 0.05)
Bahrain	137 (38 to 247)	84.15 (23.02 to 151.08)	253 (67 to 464)	85.18 (22.74 to 156.305)	0.84 (-0.496 to 5.026)	-0.05 (-0.14 to 0.04)
Bangladesh	19347 (3898 to 37610)	39.55 (7.97 to 76.89)	18189 (4868 to 34101)	39.74 (10.64 to 74.51)	-0.06 (-0.753 to 4.054)	-0.1 (-0.19 to -0.01)
Barbados	55 (15 to 98)	87.781 (23.95 to 157.76)	39 (12 to 70)	83.55 (25.16 to 147.76)	-0.281 (-0.806 to 1.626)	-0.31 (-0.41 to -0.22)
Belarus	1296 (349 to 2302)	53.921 (14.52 to 95.77)	705 (186 to 1309)	44.66 (11.806 to 82.926)	-0.456 (-0.853 to 0.968)	-0.49 (-0.57 to -0.41)
Belgium	1429 (333 to 2437)	79.09 (18.46 to 134.94)	1570 (431 to 2742)	82.13 (22.53 to 143.38)	0.099 (-0.72 to 2.664)	0.04 (-0.01 to 0.09)
Belize	56 (12 to 108)	68.648 (14.52 to 132.39)	91 (25 to 168)	73.57 (20.06 to 136.37)	0.612 (-0.6 to 6.354)	0.02 (-0.09 to 0.13)
Benin	2105 (334 to 4466)	86.916 (13.809 to 184.4)	4792 (1134 to 9631)	78.82 (18.65 to 158.39)	1.277 (-0.515 to 12.421)	-0.35 (-0.42 to -0.28)
Bermuda	11 (3 to 19)	89.7 (22.315 to 162.42)	7 (2 to 13)	87.08 (26.28 to 151.91)	-0.313 (-0.791 to 1.531)	-0.09 (-0.15 to -0.02)
Bhutan	112 (17 to 245)	42.9 (6.493 to 93.29)	86 (22 to 167)	45.755 (11.82 to 89.33)	-0.239 (-0.811 to 3.057)	0.2 (0.14 to 0.26)
Bolivia (Plurinational State of)	1960 (433 to 3766)	72.985 (16.11 to 140.21)	2115 (527 to 3990)	60.655 (15.125 to 114.433)	0.079 (-0.744 to 3.726)	-0.38 (-0.65 to -0.11)
Bosnia and Herzegovina	689 (187 to 1235)	62.88 (17.11 to 112.75)	327 (89 to 596)	66.62 (18.12 to 121.421)	-0.526 (-0.883 to 0.772)	0.23 (0.16 to 0.3)
Botswana	388 (86 to 778)	65.69 (14.544 to 131.84)	602 (170 to 1133)	86.19 (24.3 to 162.27)	0.552 (-0.554 to 5.043)	0.76 (0.69 to 0.83)
Brazil	43685 (25436 to 67748)	84.1 (48.97 to 130.42)	33861 (20242 to 50037)	70.27 (42.009 to 103.84)	-0.225 (-0.44 to 0.129)	-0.4 (-0.74 to -0.06)
Brunei Darussalam	73 (21 to 128)	80.57 (22.72 to 141.72)	68 (20 to 115)	71.36 (20.62 to 121.87)	-0.075 (-0.664 to 2.35)	-0.49 (-0.59 to -0.39)
Bulgaria	1174 (349 to 2185)	67.64 (20.11 to 125.87)	671 (182 to 1185)	68.72 (18.657 to 121.443)	-0.429 (-0.838 to 0.957)	0.16 (0.08 to 0.24)
Burkina Faso	3625 (618 to 8357)	76.82 (13.1 to 177.09)	8031 (1358 to 16335)	77.424 (13.09 to 157.49)	1.215 (-0.557 to 10.358)	-0.07 (-0.14 to 0.01)
Burundi	2259 (358 to 4792)	86.173 (13.663 to 182.815)	4115 (768 to 8804)	70.29 (13.12 to 150.39)	0.822 (-0.632 to 11.178)	-1.39 (-1.62 to -1.16)
Cabo Verde	110 (21 to 224)	70.1 (13.582 to 142.29)	121 (29 to 221)	84.47 (20.323 to 154.469)	0.097 (-0.762 to 4.77)	0.59 (0.49 to 0.69)
Cambodia	2173 (433 to 4535)	46.63 (9.29 to 97.31)	2511 (796 to 4957)	49.074 (15.55 to 96.88)	0.155 (-0.67 to 5.002)	0.4 (0.28 to 0.53)
Cameroon	3998 (697 to 8259)	81.882 (14.278 to 169.17)	10584 (2776 to 19653)	78.594 (20.61 to 145.93)	1.648 (-0.334 to 11.23)	-0.11 (-0.17 to -0.05)
Canada	2423 (769 to 3936)	42.124 (13.367 to 68.433)	2938 (734 to 5308)	47.61 (11.9 to 86.01)	0.213 (-0.613 to 2.848)	0.4 (0.12 to 0.67)
Central African Republic	917 (183 to 1958)	74.98 (14.982 to 160.106)	1602 (319 to 3157)	70.15 (13.96 to 138.21)	0.748 (-0.612 to 9.392)	-0.15 (-0.25 to -0.04)
Chad	2018 (276 to 4550)	68.95 (9.445 to 155.5)	6606 (1139 to 13431)	73.28 (12.636 to 148.988)	2.274 (-0.233 to 15.697)	0.24 (0.19 to 0.3)
Chile	3290 (1018 to 5785)	82.83 (25.627 to 145.64)	3124 (916 to 5519)	85.56 (25.07 to 151.14)	-0.05 (-0.749 to 2.411)	0.06 (-0.03 to 0.15)
China	98444 (56842 to 147536)	30.921 (17.85 to 46.34)	100053 (59912 to 150960)	38.536 (23.076 to 58.144)	0.016 (-0.278 to 0.444)	0.17 (-0.06 to 0.4)

Colombia	11078 (2537 to 20461)	94.99 (21.75 to 175.44)	9509 (2319 to 17945)	89.59 (21.854 to 169.08)	-0.142 (-0.792 to 2.81)	-0.3 (-0.41 to -0.2)
Comoros	170 (29 to 353)	79.97 (13.77 to 165.776)	201 (58 to 381)	83.84 (24 to 158.67)	0.184 (-0.685 to 5.946)	-0.08 (-0.16 to 0.01)
Congo	966 (187 to 2034)	91.72 (17.8 to 193.13)	1787 (504 to 3303)	92.604 (26.1 to 171.18)	0.85 (-0.538 to 8.935)	0 (-0.08 to 0.08)
Cook Islands	3 (1 to 6)	51.57 (15.48 to 96.901)	2 (1 to 4)	53.35 (15.94 to 96.1)	-0.406 (-0.836 to 1.436)	0.18 (0.12 to 0.25)
Costa Rica	937 (251 to 1792)	83.33 (22.33 to 159.43)	879 (251 to 1621)	86.43 (24.695 to 159.37)	-0.061 (-0.742 to 2.434)	0.09 (0.04 to 0.15)
Côte d'Ivoire	4607 (825 to 9243)	80.78 (14.47 to 162.06)	9553 (2318 to 18681)	82.556 (20.03 to 161.432)	1.073 (-0.526 to 9.034)	-0.14 (-0.23 to -0.05)
Croatia	708 (175 to 1247)	71.748 (17.68 to 126.37)	418 (110 to 737)	70.06 (18.403 to 123.43)	-0.409 (-0.855 to 1.562)	0.03 (-0.03 to 0.1)
Cuba	1676 (453 to 3114)	66.92 (18.101 to 124.36)	1166 (311 to 2102)	65.597 (17.519 to 118.32)	-0.304 (-0.816 to 1.791)	0.03 (-0.04 to 0.1)
Cyprus	136 (36 to 243)	68.721 (18.051 to 122.63)	155 (45 to 265)	70.802 (20.65 to 120.999)	0.138 (-0.693 to 3.559)	0.07 (0.03 to 0.12)
Czechia	1558 (436 to 2780)	70.68 (19.764 to 126.141)	1229 (334 to 2193)	71.63 (19.43 to 127.798)	-0.211 (-0.783 to 1.519)	0.07 (-0.01 to 0.15)
Democratic People's Republic of Korea	2134 (479 to 4029)	35.86 (8.05 to 67.72)	1535 (404 to 2951)	32.15 (8.466 to 61.82)	-0.281 (-0.814 to 2.123)	-0.38 (-0.43 to -0.33)
Democratic Republic of the Congo	14533 (2493 to 29688)	82.092 (14.08 to 167.695)	27041 (5558 to 54174)	71.16 (14.63 to 142.57)	0.861 (-0.583 to 10.085)	-0.2 (-0.38 to -0.02)
Denmark	557 (175 to 1029)	63.077 (19.76 to 116.46)	486 (128 to 846)	50.935 (13.389 to 88.65)	-0.128 (-0.718 to 1.666)	-0.92 (-1.02 to -0.82)
Djibouti	147 (27 to 311)	84.69 (15.514 to 178.66)	379 (95 to 707)	91.83 (22.89 to 171.12)	1.573 (-0.356 to 12.288)	0.1 (0.05 to 0.15)
Dominica	22 (5 to 41)	89.95 (21.85 to 164.81)	15 (5 to 26)	106.53 (33.25 to 193.34)	-0.347 (-0.81 to 1.723)	0.46 (0.32 to 0.6)
Dominican Republic	1846 (387 to 3564)	68.47 (14.37 to 132.21)	2272 (531 to 3984)	77.32 (18.09 to 135.59)	0.231 (-0.639 to 3.751)	0.4 (0.32 to 0.48)
Ecuador	3891 (831 to 7145)	100.677 (21.49 to 184.84)	6090 (1878 to 10894)	120.091 (37.039 to 214.83)	0.565 (-0.534 to 5.746)	0.08 (-0.07 to 0.24)
Egypt	15151 (3222 to 30023)	68.305 (14.52 to 135.35)	26351 (7012 to 47987)	71.5 (19.03 to 130.2)	0.739 (-0.536 to 7.613)	0.53 (0.27 to 0.79)
El Salvador	1503 (349 to 3053)	69.63 (16.17 to 141.46)	1385 (354 to 2506)	76.156 (19.449 to 137.78)	-0.078 (-0.762 to 2.62)	0.23 (0.16 to 0.3)
Equatorial Guinea	163 (28 to 341)	82.58 (14.126 to 173.2)	682 (181 to 1226)	116.66 (30.93 to 209.57)	3.197 (-0.036 to 28.768)	1.43 (1.19 to 1.67)
Eritrea	1231 (218 to 2570)	77.35 (13.72 to 161.432)	2042 (474 to 4058)	80.888 (18.76 to 160.75)	0.659 (-0.612 to 9.767)	-0.01 (-0.17 to 0.15)
Estonia	175 (45 to 311)	50.227 (12.805 to 89.223)	117 (33 to 196)	54.15 (15.141 to 90.566)	-0.332 (-0.798 to 1.413)	0.65 (0.49 to 0.81)
Eswatini	249 (46 to 479)	64.574 (12.03 to 124.21)	308 (77 to 550)	74.641 (18.564 to 133.17)	0.236 (-0.742 to 5.807)	0.47 (0.36 to 0.58)
Ethiopia	12535 (4488 to 22123)	51.45 (18.42 to 90.8)	25426 (12952 to 41084)	57.33 (29.2 to 92.64)	1.028 (-0.015 to 4.486)	-0.14 (-0.27 to -0.02)
Fiji	143 (40 to 260)	50.92 (14.2 to 92.49)	153 (43 to 261)	56.048 (15.671 to 95.65)	0.066 (-0.665 to 3.707)	0.26 (0.21 to 0.3)
Finland	542 (170 to 947)	56.14 (17.59 to 98.13)	538 (158 to 956)	63.499 (18.69 to 112.85)	-0.007 (-0.713 to 1.833)	0.29 (0.23 to 0.35)
France	9960 (2881 to 17397)	85.02 (24.59 to 148.51)	11097 (3684 to 19659)	95.6 (31.74 to 169.364)	0.114 (-0.588 to 2.76)	0.3 (0.18 to 0.42)
Gabon	436 (86 to 802)	106.937 (21.02 to 196.757)	711 (168 to 1293)	111.268 (26.24 to 202.3)	0.632 (-0.595 to 6.597)	0.12 (0.05 to 0.19)
Gambia	324 (61 to 661)	70.14 (13.16 to 143.375)	693 (152 to 1329)	69.72 (15.281 to 133.79)	1.141 (-0.543 to 10.382)	-0.28 (-0.41 to -0.15)
Georgia	951 (235 to 1813)	69.47 (17.157 to 132.464)	492 (143 to 875)	66.83 (19.47 to 118.96)	-0.483 (-0.862 to 0.965)	0.11 (-0.12 to 0.33)
Germany	14113 (4351 to 24254)	109.014 (33.612 to 187.35)	13946 (4026 to 23762)	116.56 (33.65 to 198.61)	-0.012 (-0.676 to 1.812)	0.84 (0.58 to 1.1)
Ghana	4914 (1007 to 9992)	73.16 (14.989 to 148.77)	10549 (2783 to 18870)	81.88 (21.6 to 146.47)	1.147 (-0.451 to 9.468)	0.51 (0.35 to 0.66)
Greece	1299 (369 to 2336)	64.192 (18.24 to 115.429)	939 (221 to 1662)	67.34 (15.81 to 119.15)	-0.277 (-0.812 to 1.235)	0.09 (0.01 to 0.16)
Greenland	8 (2 to 15)	56.84 (15.204 to 103.02)	6 (2 to 11)	53.936 (15.52 to 96.75)	-0.216 (-0.761 to 1.715)	-0.19 (-0.23 to -0.16)
Grenada	24 (6 to 45)	71.92 (18.89 to 133.54)	18 (4 to 33)	80.669 (20.19 to 149.1)	-0.267 (-0.781 to 1.717)	0.24 (0.16 to 0.32)
Guam	22 (5 to 37)	51.788 (12.925 to 89.82)	19 (6 to 34)	52.97 (16.56 to 93.2)	-0.103 (-0.706 to 2.435)	0.13 (0.09 to 0.17)
Guatemala	3048 (655 to 6097)	75.05 (16.13 to 150.13)	3947 (1074 to 7011)	80 (21.774 to 142.095)	0.295 (-0.662 to 5.078)	0.18 (0.12 to 0.23)
Guinea	2188 (401 to 4779)	79.52 (14.56 to 173.66)	4547 (886 to 9429)	75.2 (14.66 to 155.96)	1.078 (-0.547 to 10.448)	-0.31 (-0.38 to -0.24)
Guinea-Bissau	402 (58 to 832)	83.24 (11.95 to 172.57)	646 (124 to 1252)	71.945 (13.857 to 139.41)	0.609 (-0.694 to 9.519)	-0.63 (-0.76 to -0.51)
Guyana	205 (50 to 375)	69.7 (16.99 to 127.733)	171 (47 to 302)	80.23 (22.03 to 141.62)	-0.164 (-0.77 to 2.127)	0.32 (0.25 to 0.4)

Haiti	1974 (355 to 3908)	72.76 (13.09 to 144.05)	2855 (742 to 5397)	65.59 (17.05 to 123.995)	0.446 (-0.688 to 6.447)	-0.35 (-0.43 to -0.27)
Honduras	1798 (337 to 3674)	81.395 (15.27 to 166.28)	2350 (507 to 4486)	71.69 (15.48 to 136.89)	0.306 (-0.702 to 5.984)	-0.53 (-0.67 to -0.39)
Hungary	1473 (360 to 2662)	69.12 (16.91 to 124.91)	933 (242 to 1675)	67.17 (17.46 to 120.622)	-0.367 (-0.814 to 1.328)	-0.09 (-0.14 to -0.05)
Iceland	35 (10 to 56)	54.45 (16.372 to 88.32)	45 (13 to 78)	66.71 (18.794 to 114.85)	0.304 (-0.598 to 2.947)	0.65 (0.52 to 0.77)
India	145542 (80142 to 220926)	44.57 (24.544 to 67.66)	160607 (97445 to 232485)	43.834 (26.6 to 63.45)	0.104 (-0.232 to 0.623)	-0.12 (-0.29 to 0.05)
Indonesia	32684 (19653 to 48966)	48.25 (29.01 to 72.29)	37066 (22113 to 56932)	55.09 (32.86 to 84.61)	0.134 (-0.218 to 0.67)	0.33 (0.27 to 0.39)
Iran (Islamic Republic of)	18027 (10723 to 27587)	71.01 (42.24 to 108.68)	16082 (9815 to 23680)	79.69 (48.64 to 117.349)	-0.108 (-0.368 to 0.245)	0.47 (0.32 to 0.63)
Iraq	5285 (1300 to 10585)	64.17 (15.785 to 128.51)	9516 (2866 to 17297)	70.69 (21.29 to 128.49)	0.8 (-0.505 to 6.222)	0.32 (0.26 to 0.38)
Ireland	693 (191 to 1228)	70.53 (19.45 to 124.963)	783 (236 to 1318)	78.55 (23.64 to 132.15)	0.13 (-0.639 to 3.117)	0.17 (0.06 to 0.27)
Israel	991 (259 to 1753)	64.61 (16.91 to 114.36)	1848 (545 to 3142)	70.319 (20.74 to 119.58)	0.865 (-0.362 to 5.742)	0.17 (0.09 to 0.24)
Italy	5667 (3244 to 8470)	61.4 (35.15 to 91.77)	4412 (2581 to 6667)	58.06 (33.96 to 87.729)	-0.221 (-0.419 to 0.029)	-0.63 (-0.81 to -0.46)
Jamaica	673 (191 to 1233)	80.57 (22.864 to 147.63)	466 (143 to 824)	79.83 (24.4 to 141.17)	-0.307 (-0.794 to 1.523)	0 (-0.05 to 0.06)
Japan	12338 (7304 to 18766)	53.44 (31.63 to 81.28)	10001 (5777 to 15545)	64.76 (37.4 to 100.65)	-0.189 (-0.329 to -0.041)	0.24 (-0.02 to 0.5)
Jordan	1114 (284 to 2027)	68.23 (17.411 to 124.07)	2571 (735 to 4620)	70.76 (20.23 to 127.163)	1.307 (-0.299 to 7.933)	0.12 (0.04 to 0.21)
Kazakhstan	3882 (965 to 7166)	74.71 (18.57 to 137.91)	4142 (1088 to 7261)	76.33 (20.043 to 133.8)	0.067 (-0.721 to 3.053)	-0.01 (-0.1 to 0.08)
Kenya	10420 (6249 to 15884)	93.28 (55.948 to 142.2)	16379 (10404 to 23937)	87.75 (55.74 to 128.243)	0.572 (0.194 to 1.199)	-0.29 (-0.4 to -0.17)
Kiribati	14 (4 to 26)	47.24 (12.06 to 89.18)	18 (4 to 34)	43.992 (10.06 to 81.95)	0.325 (-0.706 to 4.81)	-0.23 (-0.29 to -0.17)
Kuwait	501 (122 to 918)	90.416 (22.01 to 165.53)	736 (196 to 1278)	87.07 (23.13 to 151.14)	0.469 (-0.613 to 4.977)	-0.41 (-0.57 to -0.24)
Kyrgyzstan	1283 (314 to 2405)	76.47 (18.7 to 143.35)	1552 (426 to 2742)	68.22 (18.73 to 120.551)	0.209 (-0.71 to 4.665)	-0.59 (-0.69 to -0.5)
Lao People's Democratic Republic	851 (137 to 1701)	46.186 (7.42 to 92.3)	1157 (282 to 2120)	50.37 (12.3 to 92.3)	0.359 (-0.7 to 8.198)	0.21 (0.14 to 0.27)
Latvia	278 (63 to 516)	48.93 (11.14 to 90.77)	151 (44 to 269)	50.76 (14.673 to 90.57)	-0.458 (-0.834 to 1.202)	0.14 (0.07 to 0.22)
Lebanon	702 (158 to 1338)	67.127 (15.13 to 127.96)	939 (275 to 1672)	73.5 (21.51 to 130.83)	0.338 (-0.621 to 4.915)	0.34 (0.26 to 0.42)
Lesotho	367 (81 to 734)	53.74 (11.85 to 107.49)	412 (92 to 790)	65.38 (14.67 to 125.333)	0.124 (-0.771 to 3.847)	0.67 (0.59 to 0.75)
Liberia	1026 (187 to 2194)	90.74 (16.59 to 194.1)	1551 (333 to 2998)	70.97 (15.242 to 137.17)	0.513 (-0.71 to 7.815)	-0.55 (-0.63 to -0.47)
Libya	1190 (265 to 2324)	65.73 (14.63 to 128.32)	989 (293 to 1691)	66.29 (19.65 to 113.39)	-0.169 (-0.773 to 3.368)	-0.06 (-0.23 to 0.11)
Lithuania	432 (123 to 739)	52.01 (14.83 to 88.98)	214 (65 to 360)	52.48 (15.88 to 88.321)	-0.505 (-0.858 to 0.797)	0.03 (-0.04 to 0.1)
Luxembourg	55 (15 to 98)	82.82 (23.27 to 147.9)	84 (27 to 143)	83.26 (26.62 to 141.72)	0.54 (-0.509 to 4.069)	0.01 (-0.01 to 0.03)
Madagascar	4688 (853 to 9624)	85.926 (15.632 to 176.386)	8971 (2259 to 18610)	76.46 (19.25 to 158.61)	0.914 (-0.558 to 7.975)	-0.45 (-0.51 to -0.4)
Malawi	3832 (743 to 8106)	84.23 (16.34 to 178.17)	6284 (1459 to 12141)	77.35 (17.96 to 149.45)	0.64 (-0.615 to 7.073)	-0.38 (-0.44 to -0.33)
Malaysia	3911 (1005 to 7264)	59.505 (15.296 to 110.509)	4931 (1325 to 8893)	64.76 (17.41 to 116.8)	0.261 (-0.648 to 3.269)	0.2 (0.17 to 0.23)
Maldives	65 (12 to 134)	62.033 (11.79 to 127.71)	61 (17 to 115)	60.96 (17.04 to 114.54)	-0.063 (-0.712 to 3.894)	0.05 (-0.05 to 0.15)
Mali	2229 (315 to 4710)	53.97 (7.62 to 114.05)	7205 (1639 to 14154)	62.238 (14.156 to 122.277)	2.232 (-0.255 to 16.67)	-0.79 (-1.2 to -0.37)
Malta	55 (14 to 98)	62.79 (15.606 to 112.35)	45 (13 to 79)	70.971 (20.683 to 122.781)	-0.173 (-0.741 to 1.951)	0.31 (0.27 to 0.36)
Marshall Islands	9 (2 to 17)	40.674 (9.35 to 77.5)	7 (2 to 15)	42.465 (10.212 to 83.43)	-0.169 (-0.836 to 3.118)	0.17 (0.07 to 0.27)
Mauritania	739 (163 to 1435)	79.97 (17.68 to 155.23)	1423 (384 to 2573)	76.79 (20.7 to 138.851)	0.925 (-0.501 to 7.8)	-0.45 (-0.75 to -0.15)
Mauritius	249 (58 to 467)	75.49 (17.495 to 141.476)	176 (56 to 309)	84.65 (26.911 to 148.88)	-0.295 (-0.804 to 2.346)	0.4 (0.35 to 0.46)
Mexico	34737 (21446 to 50619)	103.95 (64.18 to 151.48)	31490 (19362 to 46585)	98.2 (60.38 to 145.275)	-0.093 (-0.322 to 0.168)	-0.48 (-0.57 to -0.39)
Micronesia (Federated States of)	21 (5 to 42)	44.91 (10.267 to 92.12)	14 (3 to 25)	44.23 (9.04 to 81.07)	-0.344 (-0.851 to 1.666)	-0.02 (-0.08 to 0.04)
Monaco	3 (1 to 5)	74.99 (19.58 to 129.463)	4 (1 to 7)	75.302 (22.293 to 131.48)	0.418 (-0.565 to 3.752)	-0.05 (-0.11 to 0)
Mongolia	566 (115 to 1065)	62.903 (12.822 to 118.348)	710 (160 to 1295)	65.35 (14.711 to 119.2)	0.254 (-0.718 to 4.974)	0.15 (0.05 to 0.25)
Montenegro	103 (28 to 184)	64.02 (17.43 to 113.89)	71 (19 to 127)	63.69 (17.18 to 114.02)	-0.314 (-0.817 to 1.513)	0.01 (-0.03 to 0.05)
Morocco	6305 (1435 to 12637)	64.43 (14.67 to 129.132)	6573 (1512 to 12154)	67.13 (15.438 to 124.12)	0.043 (-0.728 to 3.188)	-0.07 (-0.12 to -0.02)

Mozambique	4506 (753 to 9651)	72.62 (12.14 to 155.56)	11541 (2136 to 22054)	80.9 (14.97 to 154.591)	1.561 (-0.452 to 13.908)	0.17 (-0.14 to 0.48)
Myanmar	8170 (1705 to 16276)	55.29 (11.54 to 110.151)	9151 (2522 to 17485)	58.606 (16.15 to 111.98)	0.12 (-0.712 to 4.748)	0.24 (0.19 to 0.29)
Namibia	389 (100 to 748)	64.7 (16.58 to 124.478)	582 (165 to 1098)	70.51 (20.01 to 133.059)	0.497 (-0.574 to 5.444)	0.27 (0.21 to 0.33)
Nauru	2 (0 to 4)	52.49 (10.09 to 97.765)	2 (1 to 4)	51.906 (13.68 to 92.91)	-0.068 (-0.759 to 3.505)	-0.04 (-0.1 to 0.02)
Nepal	3880 (617 to 8480)	46.058 (7.32 to 100.65)	4606 (1063 to 8763)	49.912 (11.52 to 94.97)	0.187 (-0.752 to 5.937)	0.36 (0.23 to 0.48)
Netherlands	1352 (389 to 2254)	49.618 (14.288 to 82.72)	1933 (601 to 3415)	72.06 (22.416 to 127.32)	0.429 (-0.593 to 4.097)	1.32 (0.93 to 1.72)
New Zealand	627 (237 to 1067)	78.31 (29.64 to 133.41)	678 (265 to 1100)	69.111 (27.025 to 111.999)	0.083 (-0.518 to 1.547)	-0.48 (-0.53 to -0.44)
Nicaragua	1358 (292 to 2697)	74.59 (16.01 to 148.103)	1440 (331 to 2783)	72.704 (16.73 to 140.54)	0.06 (-0.759 to 4.164)	-0.14 (-0.16 to -0.13)
Niger	2913 (353 to 6475)	71.71 (8.68 to 159.38)	8103 (1329 to 18201)	63.48 (10.41 to 142.6)	1.781 (-0.484 to 15.809)	-0.66 (-0.74 to -0.59)
Nigeria	34536 (20124 to 52126)	88.27 (51.43 to 133.231)	88167 (56372 to 122849)	86.787 (55.49 to 120.93)	1.553 (0.963 to 2.588)	-0.15 (-0.44 to 0.15)
Niue	0 (0 to 1)	48.927 (8.79 to 91.47)	0 (0 to 0)	54.08 (14.967 to 93.91)	-0.47 (-0.823 to 1.058)	0.37 (0.32 to 0.42)
North Macedonia	344 (102 to 625)	65.377 (19.37 to 118.65)	202 (57 to 362)	61.746 (17.33 to 110.4)	-0.413 (-0.855 to 0.818)	-0.17 (-0.21 to -0.14)
Northern Mariana Islands	6 (1 to 12)	52.96 (11.55 to 97.24)	6 (1 to 10)	50.123 (11.49 to 90.16)	-0.123 (-0.795 to 2.771)	-0.32 (-0.43 to -0.21)
Norway	650 (345 to 979)	81.394 (43.21 to 122.67)	758 (426 to 1179)	82.09 (46.1 to 127.628)	0.167 (-0.198 to 0.584)	-0.06 (-0.11 to -0.02)
Oman	553 (102 to 1045)	65.78 (12.16 to 124.33)	982 (267 to 1719)	80.31 (21.85 to 140.519)	0.777 (-0.449 to 6.956)	0.63 (0.54 to 0.73)
Pakistan	24642 (9548 to 43930)	50.042 (19.39 to 89.21)	46121 (21337 to 74446)	53.98 (24.97 to 87.13)	0.872 (-0.046 to 3.375)	0.16 (-0.01 to 0.33)
Palau	2 (1 to 4)	53.36 (11.98 to 97.969)	2 (0 to 3)	53.329 (14.63 to 93.06)	-0.285 (-0.819 to 2.201)	-0.03 (-0.08 to 0.02)
Palestine	632 (120 to 1303)	65.24 (12.431 to 134.53)	1263 (357 to 2285)	67.62 (19.123 to 122.38)	0.999 (-0.488 to 11.43)	0.14 (0.11 to 0.18)
Panama	673 (155 to 1291)	80.651 (18.57 to 154.79)	1098 (304 to 1896)	95.21 (26.39 to 164.43)	0.633 (-0.52 to 7.338)	0.56 (0.52 to 0.6)
Papua New Guinea	666 (140 to 1394)	39.165 (8.22 to 81.99)	1557 (395 to 3023)	39.74 (10.081 to 77.182)	1.338 (-0.424 to 9.305)	0.06 (0.04 to 0.09)
Paraguay	1083 (267 to 2017)	64.88 (15.997 to 120.79)	1391 (405 to 2627)	69.28 (20.15 to 130.86)	0.284 (-0.614 to 3.756)	0.17 (0.14 to 0.19)
Peru	7413 (1633 to 14179)	89.3 (19.68 to 170.82)	8739 (2252 to 15909)	91.627 (23.61 to 166.81)	0.179 (-0.673 to 4.206)	-0.92 (-1.28 to -0.57)
Philippines	13809 (8507 to 19902)	54.765 (33.74 to 78.93)	18314 (11469 to 27147)	53.866 (33.73 to 79.85)	0.326 (0.068 to 0.667)	-0.05 (-0.09 to -0.01)
Poland	4960 (3057 to 7529)	51.791 (31.926 to 78.62)	3594 (2185 to 5371)	61.06 (37.13 to 91.258)	-0.275 (-0.472 to -0.043)	0.71 (0.6 to 0.81)
Portugal	1090 (245 to 2006)	51.52 (11.58 to 94.8)	830 (231 to 1465)	60.91 (16.962 to 107.578)	-0.239 (-0.794 to 2.008)	0.12 (0.01 to 0.23)
Puerto Rico	815 (252 to 1451)	81.87 (25.29 to 145.7)	368 (98 to 658)	82.91 (22.06 to 148.07)	-0.548 (-0.863 to 0.458)	-0.11 (-0.21 to -0.01)
Qatar	116 (29 to 211)	92.42 (23.36 to 168.7)	439 (137 to 751)	88.93 (27.75 to 151.981)	2.801 (0.03 to 10.956)	-0.17 (-0.22 to -0.13)
Republic of Korea	7609 (2036 to 13690)	66.92 (17.9 to 120.4)	3856 (934 to 6529)	63.482 (15.368 to 107.489)	-0.493 (-0.863 to 0.588)	-0.39 (-0.46 to -0.31)
Republic of Moldova	651 (172 to 1206)	52.713 (13.89 to 97.56)	238 (72 to 439)	45.6 (13.781 to 84.1)	-0.634 (-0.896 to 0.356)	-0.59 (-0.62 to -0.56)
Romania	3905 (1005 to 7103)	70.134 (18.05 to 127.561)	2049 (528 to 3656)	68.08 (17.53 to 121.473)	-0.475 (-0.842 to 0.899)	-0.04 (-0.11 to 0.04)
Russian Federation	17725 (11038 to 26546)	51.082 (31.81 to 76.5)	11822 (7206 to 18098)	45.33 (27.63 to 69.4)	-0.333 (-0.444 to -0.229)	-0.34 (-0.44 to -0.25)
Rwanda	3004 (577 to 6045)	88.54 (17 to 178.17)	4010 (839 to 7583)	80.68 (16.88 to 152.56)	0.335 (-0.729 to 5.687)	-0.45 (-0.56 to -0.34)
Saint Kitts and Nevis	13 (3 to 24)	94.76 (24.23 to 167.69)	9 (3 to 16)	95.247 (26.335 to 166.95)	-0.299 (-0.779 to 1.687)	0.16 (0.1 to 0.22)
Saint Lucia	44 (11 to 81)	84.76 (20.87 to 157.63)	25 (6 to 46)	83.758 (20.394 to 155.81)	-0.431 (-0.862 to 1.203)	-0.05 (-0.1 to 0)
Saint Vincent and the Grenadines	31 (7 to 60)	74.254 (16.98 to 145.49)	20 (6 to 37)	81.921 (22.81 to 147.08)	-0.33 (-0.82 to 2.203)	0.14 (0.04 to 0.25)
Samoa	33 (7 to 63)	45.62 (9.74 to 88.46)	38 (10 to 69)	47.45 (12.98 to 86.11)	0.167 (-0.678 to 4.764)	0.1 (0.01 to 0.18)
San Marino	3 (1 to 5)	69.56 (17.52 to 121.457)	3 (1 to 5)	71.33 (18.35 to 123.345)	0.1 (-0.67 to 2.75)	0 (-0.08 to 0.09)
Sao Tome and Principe	44 (8 to 87)	76.84 (13.97 to 154.22)	66 (19 to 122)	84.61 (23.89 to 156.62)	0.513 (-0.631 to 6.611)	0.31 (0.25 to 0.38)
Saudi Arabia	5290 (1107 to 9979)	80.72 (16.89 to 152.28)	7319 (1986 to 12853)	96.748 (26.25 to 169.89)	0.384 (-0.607 to 4.627)	0.55 (0.51 to 0.6)
Senegal	3111 (472 to 6121)	85.212 (12.934 to 167.651)	4932 (1011 to 9892)	77.55 (15.89 to 155.52)	0.585 (-0.696 to 9.393)	-0.31 (-0.4 to -0.23)
Serbia	1711 (445 to 2996)	78.871 (20.5 to 138.12)	894 (253 to 1506)	67.35 (19.06 to 113.41)	-0.477 (-0.846 to 0.912)	-0.43 (-0.53 to -0.33)
Seychelles	13 (3 to 26)	56.8 (14.251 to 108.28)	15 (4 to 28)	63.23 (17.07 to 119.665)	0.098 (-0.664 to 2.91)	0.41 (0.35 to 0.47)
Sierra Leone	1471 (221 to 2948)	81.14 (12.19 to 162.64)	2562 (488 to 4982)	71.63 (13.64 to 139.31)	0.742 (-0.639 to 8.547)	-0.44 (-0.5 to -0.38)
Singapore	358 (103 to 637)	55.092 (15.877 to 98.08)	516 (141 to 912)	63.529 (17.38 to 112.32)	0.442 (-0.63 to 4.301)	0.49 (0.41 to 0.56)

Slovakia	848 (210 to 1525)	63.935 (15.866 to 115.04)	589 (168 to 1018)	68.724 (19.64 to 118.89)	-0.305 (-0.799 to 1.117)	0.2 (0.11 to 0.29)
Slovenia	278 (82 to 482)	67.11 (19.76 to 116.55)	207 (62 to 359)	66.342 (19.86 to 114.966)	-0.254 (-0.795 to 1.429)	0.04 (-0.04 to 0.13)
Solomon Islands	60 (11 to 126)	38.36 (6.974 to 81.12)	103 (22 to 204)	39.53 (8.57 to 78.61)	0.721 (-0.617 to 7.263)	0.1 (0.04 to 0.16)
Somalia	2260 (286 to 5161)	58.013 (7.33 to 132.47)	5369 (825 to 12156)	51.98 (7.99 to 117.68)	1.376 (-0.571 to 16.147)	-0.51 (-0.6 to -0.42)
South Africa	10462 (6034 to 16151)	76.85 (44.32 to 118.636)	10801 (6221 to 16829)	71.04 (40.91 to 110.68)	0.032 (-0.343 to 0.63)	-0.57 (-0.8 to -0.34)
South Sudan	2266 (469 to 4706)	86.34 (17.86 to 179.35)	3256 (720 to 6521)	75.808 (16.77 to 151.84)	0.437 (-0.722 to 5.301)	-0.44 (-0.53 to -0.34)
Spain	4477 (1190 to 8263)	57.135 (15.192 to 105.45)	3740 (1083 to 6740)	57.72 (16.71 to 103.99)	-0.165 (-0.735 to 2.424)	0.17 (0 to 0.35)
Sri Lanka	4079 (838 to 7750)	73.72 (15.153 to 140.061)	3747 (851 to 6783)	73.413 (16.68 to 132.89)	-0.081 (-0.798 to 2.941)	-0.02 (-0.07 to 0.03)
Sudan	5462 (1008 to 11318)	61.42 (11.338 to 127.276)	10594 (2969 to 19406)	63.86 (17.9 to 116.98)	0.94 (-0.519 to 8.592)	0.19 (0.14 to 0.25)
Suriname	100 (23 to 193)	76.59 (17.29 to 147.8)	122 (37 to 216)	85.257 (25.817 to 150.79)	0.224 (-0.608 to 5.182)	0.3 (0.26 to 0.35)
Sweden	865 (323 to 1503)	56.01 (20.93 to 97.29)	944 (348 to 1605)	51.87 (19.13 to 88.13)	0.092 (-0.518 to 1.669)	-0.01 (-0.13 to 0.11)
Switzerland	807 (228 to 1411)	69.84 (19.77 to 122.11)	953 (259 to 1637)	71.55 (19.45 to 122.86)	0.181 (-0.634 to 3.193)	0 (-0.04 to 0.04)
Syrian Arab Republic	3387 (682 to 6642)	57.192 (11.521 to 112.16)	2262 (677 to 3951)	61.743 (18.49 to 107.854)	-0.332 (-0.826 to 2.207)	0.14 (-0.05 to 0.32)
Taiwan (Province of China)	2294 (689 to 4038)	41.64 (12.5 to 73.31)	1354 (310 to 2359)	45.95 (10.517 to 80.05)	-0.41 (-0.826 to 1.164)	0.24 (0.16 to 0.32)
Tajikistan	1959 (456 to 3638)	84.36 (19.65 to 156.68)	2428 (654 to 4230)	67.75 (18.239 to 118)	0.24 (-0.662 to 3.966)	-0.92 (-1 to -0.84)
Thailand	8606 (1955 to 17253)	51.049 (11.6 to 102.34)	5946 (1850 to 10774)	60.88 (18.94 to 110.32)	-0.309 (-0.788 to 2.108)	0.47 (0.43 to 0.51)
Timor-Leste	166 (32 to 341)	49.993 (9.61 to 102.601)	275 (78 to 515)	52.75 (14.985 to 98.875)	0.652 (-0.543 to 8.432)	0.17 (0.11 to 0.22)
Togo	1930 (387 to 3857)	109.53 (21.972 to 218.8)	2668 (592 to 5078)	80.62 (17.89 to 153.444)	0.382 (-0.679 to 5.771)	-1.37 (-1.61 to -1.12)
Tokelau	0 (0 to 1)	42.89 (10.451 to 87.678)	0 (0 to 0)	43.77 (9.98 to 82.8)	-0.337 (-0.857 to 2.074)	0.08 (0.02 to 0.15)
Tonga	18 (4 to 33)	43.04 (9.79 to 79.33)	18 (5 to 32)	45.51 (13.35 to 80.73)	-0.013 (-0.74 to 2.913)	0.08 (0.04 to 0.13)
Trinidad and Tobago	361 (98 to 662)	88.74 (24.02 to 162.86)	248 (70 to 449)	91.095 (25.65 to 164.81)	-0.312 (-0.798 to 1.642)	0.02 (-0.05 to 0.09)
Tunisia	1804 (464 to 3387)	58.096 (14.931 to 109.05)	1874 (490 to 3534)	67.764 (17.73 to 127.79)	0.039 (-0.742 to 3.13)	0.45 (0.35 to 0.55)
Türkiye	15119 (3312 to 28867)	73.79 (16.17 to 140.892)	16106 (4784 to 29737)	86.97 (25.83 to 160.56)	0.065 (-0.694 to 3.286)	0.99 (0.82 to 1.15)
Turkmenistan	1085 (291 to 2025)	72.3 (19.37 to 134.91)	1207 (302 to 2121)	79.17 (19.85 to 139.172)	0.112 (-0.74 to 2.593)	0.34 (0.31 to 0.38)
Tuvalu	2 (0 to 3)	44.391 (11.57 to 83.65)	2 (0 to 3)	44.08 (11.55 to 81.22)	0.065 (-0.724 to 2.998)	-0.1 (-0.15 to -0.05)
Uganda	8413 (1199 to 17340)	99.93 (14.24 to 205.947)	18825 (3863 to 36363)	94.91 (19.48 to 183.321)	1.238 (-0.527 to 12.704)	-0.29 (-0.49 to -0.1)
Ukraine	6643 (2156 to 11277)	58.402 (18.95 to 99.143)	3130 (846 to 5479)	49.329 (13.33 to 86.36)	-0.529 (-0.847 to 0.57)	-0.67 (-0.72 to -0.62)
United Arab Emirates	590 (153 to 1084)	100.032 (26.03 to 183.99)	1213 (375 to 2065)	90.58 (28.04 to 154.23)	1.057 (-0.361 to 5.747)	-0.46 (-0.58 to -0.34)
United Kingdom	9318 (5920 to 13231)	85.32 (54.21 to 121.16)	10908 (6441 to 16715)	92.57 (54.66 to 141.86)	0.171 (-0.011 to 0.338)	0.14 (-0.14 to 0.41)
United Republic of Tanzania	9380 (2172 to 19498)	77.68 (17.989 to 161.47)	21972 (5172 to 43123)	90.039 (21.19 to 176.71)	1.342 (-0.323 to 12.009)	-0.03 (-0.19 to 0.12)
United States of America	31794 (18861 to 48652)	56.87 (33.735 to 87.02)	35393 (18799 to 54585)	59.55 (31.63 to 91.84)	0.113 (-0.135 to 0.346)	0.09 (-0.04 to 0.23)
United States Virgin Islands	29 (8 to 52)	91.73 (24.769 to 161.55)	11 (3 to 21)	85.87 (25.56 to 154.23)	-0.608 (-0.884 to 0.571)	-0.39 (-0.55 to -0.23)
Uruguay	502 (147 to 901)	61.34 (17.98 to 110.05)	463 (99 to 863)	70.17 (15.04 to 130.78)	-0.078 (-0.784 to 2.429)	0.51 (0.49 to 0.54)
Uzbekistan	6038 (1638 to 11063)	70.57 (19.14 to 129.3)	7590 (2143 to 13137)	75.215 (21.23 to 130.19)	0.257 (-0.636 to 4.485)	0.05 (-0.1 to 0.2)
Vanuatu	27 (6 to 53)	40.04 (8.86 to 77.84)	48 (10 to 91)	41.23 (8.43 to 77.931)	0.762 (-0.582 to 7.983)	0.14 (0.09 to 0.2)
Venezuela (Bolivarian Republic of)	6247 (1409 to 12093)	88.06 (19.87 to 170.47)	5637 (1685 to 10424)	85.1 (25.434 to 157.36)	-0.098 (-0.747 to 3.05)	-0.07 (-0.16 to 0.01)
Viet Nam	11436 (2456 to 23084)	43.13 (9.27 to 87.07)	13104 (3256 to 23401)	52.918 (13.15 to 94.503)	0.146 (-0.745 to 4.67)	0.83 (0.7 to 0.96)
Yemen	3634 (588 to 7751)	51.227 (8.29 to 109.26)	7492 (1292 to 14630)	54.338 (9.37 to 106.11)	1.062 (-0.616 to 12.78)	0.24 (0.07 to 0.4)
Zambia	3835 (748 to 7769)	102.14 (19.924 to 206.94)	8714 (2093 to 16573)	105.35 (25.3 to 200.37)	1.272 (-0.37 to 9.776)	-0.66 (-0.96 to -0.37)
Zimbabwe	2939 (627 to 5697)	61.023 (13.03 to 118.29)	3875 (905 to 7547)	61.574 (14.376 to 119.91)	0.319 (-0.691 to 5.485)	-0.15 (-0.26 to -0.04)

Abbreviations: EAPC, estimated annual percentage change; SDI, Sociodemographic Index; UI, uncertainty interval.

[a] EAPC is expressed as 95% CIs.

Supplementary Table 4. Deaths of Childhood Idiopathic Epilepsy at the National Level

Location	1990		2021		1990-2021	
	Deaths cases	Deaths rate	Deaths cases	Deaths rate	Cases change	EAPC[a]
Afghanistan	168 (86 to 262)	3.906 (2.003 to 6.079)	326 (200 to 445)	2.298 (1.409 to 3.134)	0.939 (0.311 to 2.031)	-1.3 (-1.49 to -1.1)
Albania	25 (12 to 34)	2.24 (1.048 to 3.086)	5 (3 to 8)	1.236 (0.67 to 1.715)	-0.781 (-0.845 to -0.688)	-2.01 (-2.26 to -1.77)
Algeria	205 (119 to 280)	1.91 (1.108 to 2.61)	117 (81 to 152)	0.878 (0.613 to 1.141)	-0.431 (-0.605 to -0.184)	-2.09 (-2.26 to -1.92)
American Samoa	0 (0 to 0)	0.253 (0.178 to 0.373)	0 (0 to 0)	0.359 (0.244 to 0.501)	0.056 (-0.381 to 0.778)	2.3 (1.69 to 2.9)
Andorra	0 (0 to 0)	0.431 (0.293 to 0.598)	0 (0 to 0)	0.165 (0.117 to 0.224)	-0.589 (-0.713 to -0.375)	-2.87 (-3.01 to -2.72)
Angola	104 (71 to 169)	2.197 (1.509 to 3.585)	207 (139 to 288)	1.357 (0.914 to 1.891)	0.997 (0.172 to 2.078)	-1.29 (-1.49 to -1.1)
Antigua and Barbuda	0 (0 to 0)	1.05 (0.871 to 1.238)	0 (0 to 0)	0.903 (0.762 to 1.058)	-0.201 (-0.369 to -0.003)	-0.98 (-1.38 to -0.58)
Argentina	39 (37 to 43)	0.389 (0.36 to 0.421)	36 (31 to 42)	0.356 (0.305 to 0.415)	-0.08 (-0.228 to 0.096)	0.61 (0.3 to 0.93)
Armenia	6 (5 to 6)	0.528 (0.467 to 0.603)	2 (1 to 2)	0.287 (0.212 to 0.38)	-0.691 (-0.782 to -0.579)	-2.21 (-2.56 to -1.86)
Australia	18 (16 to 20)	0.473 (0.43 to 0.519)	12 (11 to 15)	0.261 (0.221 to 0.309)	-0.307 (-0.414 to -0.156)	-1.75 (-2.01 to -1.48)
Austria	4 (4 to 4)	0.292 (0.267 to 0.32)	3 (2 to 3)	0.222 (0.189 to 0.257)	-0.27 (-0.376 to -0.121)	-0.18 (-0.42 to 0.07)
Azerbaijan	44 (32 to 54)	1.813 (1.299 to 2.225)	32 (22 to 41)	1.351 (0.928 to 1.74)	-0.275 (-0.457 to -0.028)	-1.48 (-1.93 to -1.03)
Bahamas	0 (0 to 1)	0.562 (0.476 to 0.677)	0 (0 to 0)	0.248 (0.19 to 0.326)	-0.556 (-0.68 to -0.394)	-2.9 (-3.18 to -2.63)
Bahrain	2 (2 to 3)	1.451 (1.104 to 2.048)	2 (1 to 3)	0.632 (0.469 to 0.899)	-0.208 (-0.391 to 0.08)	-2.77 (-2.9 to -2.63)
Bangladesh	624 (402 to 952)	1.275 (0.82 to 1.946)	256 (153 to 468)	0.558 (0.335 to 1.022)	-0.59 (-0.752 to -0.217)	-3.05 (-3.25 to -2.85)
Barbados	0 (0 to 1)	0.783 (0.642 to 0.938)	0 (0 to 0)	0.358 (0.256 to 0.491)	-0.655 (-0.761 to -0.499)	-2 (-2.33 to -1.67)
Belarus	16 (14 to 20)	0.68 (0.562 to 0.84)	3 (3 to 4)	0.21 (0.164 to 0.262)	-0.798 (-0.853 to -0.725)	-4.11 (-4.39 to -3.84)
Belgium	8 (8 to 9)	0.467 (0.419 to 0.519)	9 (8 to 11)	0.466 (0.393 to 0.552)	0.057 (-0.14 to 0.312)	0.06 (-0.14 to 0.26)
Belize	1 (1 to 1)	1.495 (1.293 to 1.706)	1 (0 to 1)	0.454 (0.365 to 0.572)	-0.543 (-0.652 to -0.402)	-3.73 (-4.01 to -3.45)
Benin	19 (12 to 27)	0.789 (0.497 to 1.108)	40 (26 to 56)	0.663 (0.423 to 0.926)	1.109 (0.371 to 2.484)	-0.41 (-0.52 to -0.3)
Bermuda	0 (0 to 0)	0.434 (0.367 to 0.509)	0 (0 to 0)	0.22 (0.146 to 0.304)	-0.636 (-0.772 to -0.486)	-2.18 (-2.64 to -1.72)
Bhutan	4 (2 to 6)	1.673 (0.808 to 2.444)	3 (2 to 5)	1.634 (1.029 to 2.441)	-0.303 (-0.61 to 0.717)	-0.41 (-0.85 to 0.04)
Bolivia (Plurinational State of)	59 (38 to 81)	2.214 (1.421 to 3.008)	33 (23 to 44)	0.94 (0.659 to 1.267)	-0.451 (-0.648 to -0.1)	-2.72 (-2.76 to -2.68)
Bosnia and Herzegovina	10 (8 to 13)	0.93 (0.711 to 1.214)	2 (2 to 3)	0.498 (0.37 to 0.673)	-0.76 (-0.835 to -0.66)	-2.42 (-2.72 to -2.12)
Botswana	7 (5 to 9)	1.128 (0.838 to 1.494)	8 (5 to 11)	1.113 (0.777 to 1.524)	0.166 (-0.241 to 0.673)	0.17 (0.07 to 0.27)
Brazil	347 (295 to 415)	0.669 (0.567 to 0.8)	287 (223 to 348)	0.596 (0.462 to 0.722)	-0.173 (-0.411 to 0.059)	0.51 (0.27 to 0.76)
Brunei Darussalam	1 (1 to 1)	0.795 (0.627 to 1.064)	1 (0 to 1)	0.557 (0.427 to 0.716)	-0.268 (-0.466 to 0.03)	-0.74 (-0.96 to -0.51)
Bulgaria	14 (12 to 16)	0.822 (0.7 to 0.948)	6 (4 to 8)	0.604 (0.432 to 0.808)	-0.587 (-0.706 to -0.408)	-1.32 (-1.57 to -1.07)
Burkina Faso	34 (21 to 51)	0.727 (0.454 to 1.081)	69 (45 to 94)	0.661 (0.429 to 0.911)	0.999 (0.34 to 2.067)	0.05 (-0.11 to 0.22)
Burundi	73 (53 to 98)	2.795 (2.011 to 3.726)	103 (66 to 151)	1.758 (1.133 to 2.574)	0.404 (-0.121 to 1.185)	-0.82 (-1.15 to -0.49)
Cabo Verde	1 (0 to 1)	0.443 (0.27 to 0.732)	0 (0 to 1)	0.345 (0.231 to 0.58)	-0.29 (-0.543 to 0.101)	-0.84 (-1.07 to -0.62)
Cambodia	24 (17 to 36)	0.525 (0.357 to 0.777)	17 (12 to 28)	0.341 (0.237 to 0.552)	-0.287 (-0.565 to 0.129)	-1.32 (-1.43 to -1.2)
Cameroon	32 (21 to 44)	0.65 (0.433 to 0.906)	69 (43 to 98)	0.515 (0.323 to 0.73)	1.197 (0.45 to 2.485)	-0.26 (-0.42 to -0.1)
Canada	20 (18 to 22)	0.347 (0.313 to 0.383)	14 (12 to 17)	0.232 (0.195 to 0.271)	-0.282 (-0.403 to -0.128)	-0.63 (-1.03 to -0.23)
Central African Republic	27 (19 to 42)	2.238 (1.556 to 3.399)	50 (34 to 70)	2.195 (1.475 to 3.059)	0.833 (0.184 to 1.923)	0.1 (-0.06 to 0.26)
Chad	19 (12 to 29)	0.654 (0.411 to 0.983)	63 (42 to 88)	0.693 (0.46 to 0.977)	2.265 (1.23 to 3.725)	0.43 (0.35 to 0.51)
Chile	32 (30 to 35)	0.809 (0.74 to 0.878)	16 (13 to 19)	0.446 (0.367 to 0.53)	-0.493 (-0.584 to -0.38)	-1.08 (-1.33 to -0.82)
China	6074 (4104 to 7330)	1.908 (1.289 to 2.302)	1246 (1018 to 1670)	0.48 (0.392 to 0.64)	-0.795 (-0.844 to -0.671)	-4.54 (-4.71 to -4.36)
Colombia	127 (113 to 144)	1.091 (0.965 to 1.239)	63 (49 to 82)	0.6 (0.465 to 0.769)	-0.502 (-0.613 to -0.354)	-0.69 (-1.14 to -0.22)
Comoros	6 (4 to 8)	2.802 (1.92 to 3.835)	5 (4 to 8)	2.159 (1.527 to 3.197)	-0.13 (-0.429 to 0.441)	-1 (-1.27 to -0.72)
Congo	17 (12 to 24)	1.602 (1.138 to 2.243)	20 (14 to 27)	1.051 (0.751 to 1.408)	0.202 (-0.219 to 0.824)	-1.15 (-1.37 to -0.94)
Cook Islands	0 (0 to 0)	0.251 (0.182 to 0.363)	0 (0 to 0)	0.267 (0.174 to 0.406)	-0.391 (-0.593 to -0.059)	-1.15 (-1.65 to -0.66)
Costa Rica	7 (7 to 8)	0.657 (0.602 to 0.725)	4 (3 to 5)	0.396 (0.337 to 0.47)	-0.455 (-0.546 to -0.337)	-1.47 (-1.7 to -1.24)
Côte d'Ivoire	33 (23 to 45)	0.582 (0.407 to 0.792)	63 (39 to 88)	0.548 (0.337 to 0.757)	0.911 (0.305 to 1.825)	0.15 (-0.1 to 0.4)
Croatia	5 (5 to 6)	0.556 (0.499 to 0.611)	2 (2 to 3)	0.357 (0.26 to 0.447)	-0.611 (-0.726 to -0.504)	-1.12 (-1.45 to -0.79)
Cuba	9 (8 to 10)	0.357 (0.328 to 0.392)	3 (2 to 3)	0.158 (0.134 to 0.186)	-0.686 (-0.746 to -0.618)	-2.03 (-2.4 to -1.67)
Cyprus	1 (1 to 1)	0.346 (0.26 to 0.454)	0 (0 to 0)	0.127 (0.09 to 0.169)	-0.595 (-0.718 to -0.449)	-2.66 (-3.09 to -2.22)
Czechia	12 (11 to 14)	0.553 (0.49 to 0.634)	6 (4 to 7)	0.33 (0.243 to 0.43)	-0.535 (-0.66 to -0.347)	-2.03 (-2.3 to -1.76)
Democratic People's Republic of Korea	64 (43 to 91)	1.075 (0.722 to 1.527)	30 (19 to 49)	0.62 (0.402 to 1.017)	-0.534 (-0.717 to -0.268)	-1.68 (-1.77 to -1.59)
Democratic Republic of the Congo	314 (218 to 449)	1.774 (1.23 to 2.537)	433 (289 to 616)	1.139 (0.761 to 1.621)	0.378 (-0.07 to 1.022)	-0.92 (-1.1 to -0.73)
Denmark	3 (2 to 4)	0.313 (0.252 to 0.408)	2 (2 to 3)	0.241 (0.203 to 0.29)	-0.167 (-0.373 to 0.123)	-0.88 (-1.08 to -0.67)
Djibouti	4 (3 to 5)	2.262 (1.612 to 3.114)	8 (5 to 12)	1.917 (1.245 to 2.821)	1.011 (0.298 to 2.001)	-0.43 (-0.8 to -0.07)
Dominica	0 (0 to 0)	1.255 (0.956 to 1.564)	0 (0 to 0)	1.384 (0.986 to 1.863)	-0.392 (-0.583 to -0.151)	0.27 (0.14 to 0.4)
Dominican Republic	38 (16 to 51)	1.419 (0.604 to 1.891)	24 (12 to 35)	0.804 (0.408 to 1.201)	-0.382 (-0.58 to -0.105)	-1.88 (-2.21 to -1.55)
Ecuador	61 (55 to 67)	1.572 (1.436 to 1.732)	41 (32 to 52)	0.814 (0.63 to 1.025)	-0.321 (-0.476 to -0.14)	-2.53 (-3.04 to -2.01)
Egypt	207 (139 to 278)	0.933 (0.625 to 1.252)	169 (125 to 213)	0.458 (0.34 to 0.58)	-0.185 (-0.465 to 0.167)	-1.55 (-1.84 to -1.26)
El Salvador	22 (15 to 28)	1.028 (0.691 to 1.279)	8 (6 to 10)	0.423 (0.305 to 0.573)	-0.653 (-0.767 to -0.42)	-2.76 (-3.2 to -2.33)
Equatorial Guinea	4 (2 to 5)	1.83 (1.258 to 2.664)	5 (2 to 10)	0.841 (0.42 to 1.645)	0.365 (-0.309 to 1.722)	-2.82 (-2.98 to -2.67)
Eritrea	41 (29 to 58)	2.606 (1.807 to 3.66)	63 (41 to 91)	2.506 (1.633 to 3.609)	0.525 (-0.008 to 1.356)	-0.17 (-0.26 to -0.07)
Estonia	5 (4 to 6)	1.414 (1.179 to 1.669)	1 (0 to 1)	0.258 (0.198 to 0.34)	-0.887 (-0.916 to -0.843)	-6.48 (-7.32 to -5.63)
Eswatini	5 (4 to 7)	1.246 (0.909 to 1.686)	5 (4 to 7)	1.253 (0.92 to 1.687)	0.076 (-0.237 to 0.517)	0.28 (0.2 to 0.35)
Ethiopia	840 (523 to 1199)	3.448 (2.145 to 4.919)	897 (671 to 1168)	2.022 (1.514 to 2.633)	0.068 (-0.314 to 0.702)	-1.9 (-2.04 to -1.77)
Fiji	2 (1 to 2)	0.628 (0.43 to 0.88)	2 (1 to 3)	0.654 (0.44 to 0.92)	0.008 (-0.351 to 0.67)	0.3 (0.11 to 0.49)

Finland	3 (2 to 3)	0.273 (0.246 to 0.31)	3 (2 to 3)	0.3 (0.247 to 0.37)	-0.037 (-0.235 to 0.22)	0.07 (-0.3 to 0.44)
France	57 (52 to 61)	0.483 (0.448 to 0.521)	47 (41 to 53)	0.409 (0.351 to 0.46)	-0.162 (-0.293 to -0.032)	0.03 (-0.2 to 0.26)
Gabon	5 (4 to 7)	1.331 (0.965 to 1.754)	7 (4 to 10)	1.066 (0.69 to 1.542)	0.256 (-0.221 to 0.958)	-0.34 (-0.52 to -0.16)
Gambia	3 (2 to 4)	0.572 (0.373 to 0.826)	5 (4 to 7)	0.531 (0.366 to 0.734)	0.998 (0.366 to 1.879)	-0.33 (-0.61 to -0.05)
Georgia	11 (10 to 12)	0.794 (0.701 to 0.9)	2 (2 to 3)	0.326 (0.261 to 0.403)	-0.779 (-0.829 to -0.711)	-2.78 (-3.17 to -2.39)
Germany	63 (58 to 67)	0.486 (0.451 to 0.518)	47 (41 to 55)	0.4 (0.34 to 0.456)	-0.245 (-0.369 to -0.093)	-0.36 (-0.63 to -0.09)
Ghana	26 (16 to 36)	0.393 (0.242 to 0.541)	52 (23 to 80)	0.404 (0.176 to 0.618)	0.971 (0.096 to 2.125)	0.86 (0.54 to 1.17)
Greece	3 (3 to 3)	0.139 (0.124 to 0.157)	3 (2 to 3)	0.193 (0.149 to 0.244)	-0.047 (-0.288 to 0.254)	1.57 (1.33 to 1.81)
Greenland	0 (0 to 0)	0.86 (0.63 to 1.193)	0 (0 to 0)	0.663 (0.453 to 1.067)	-0.36 (-0.592 to -0.06)	-1.08 (-1.85 to -0.3)
Grenada	0 (0 to 0)	1.047 (0.861 to 1.262)	0 (0 to 0)	0.508 (0.411 to 0.61)	-0.683 (-0.75 to -0.599)	-1.72 (-1.94 to -1.49)
Guam	0 (0 to 0)	0.06 (0.04 to 0.118)	0 (0 to 0)	0.1 (0.06 to 0.137)	0.322 (-0.353 to 1.46)	3.07 (2.42 to 3.71)
Guatemala	98 (86 to 111)	2.422 (2.121 to 2.742)	68 (53 to 87)	1.376 (1.071 to 1.767)	-0.31 (-0.479 to -0.097)	-1.24 (-1.61 to -0.87)
Guinea	24 (14 to 35)	0.855 (0.52 to 1.274)	37 (23 to 51)	0.609 (0.382 to 0.837)	0.564 (0.003 to 1.562)	-0.62 (-0.79 to -0.44)
Guinea-Bissau	4 (2 to 6)	0.8 (0.512 to 1.15)	5 (3 to 8)	0.602 (0.389 to 0.87)	0.4 (-0.047 to 1.099)	-0.39 (-0.69 to -0.1)
Guyana	4 (4 to 5)	1.528 (1.261 to 1.836)	2 (2 to 3)	1.043 (0.771 to 1.389)	-0.504 (-0.651 to -0.318)	-0.31 (-0.65 to 0.03)
Haiti	71 (40 to 132)	2.626 (1.487 to 4.848)	70 (44 to 118)	1.615 (1.008 to 2.714)	-0.014 (-0.309 to 0.532)	-1.32 (-1.49 to -1.15)
Honduras	58 (36 to 74)	2.64 (1.643 to 3.36)	44 (27 to 67)	1.354 (0.833 to 2.042)	-0.239 (-0.554 to 0.239)	-2.06 (-2.17 to -1.95)
Hungary	11 (9 to 12)	0.499 (0.434 to 0.568)	4 (3 to 5)	0.275 (0.2 to 0.357)	-0.641 (-0.745 to -0.528)	-1.22 (-1.51 to -0.93)
Iceland	0 (0 to 0)	0.273 (0.241 to 0.302)	0 (0 to 0)	0.262 (0.216 to 0.308)	0.025 (-0.184 to 0.275)	-0.01 (-0.55 to 0.54)
India	7286 (3489 to 9620)	2.232 (1.069 to 2.946)	4601 (2699 to 6252)	1.256 (0.737 to 1.706)	-0.369 (-0.535 to -0.085)	-1.75 (-1.98 to -1.52)
Indonesia	104 (33 to 155)	0.153 (0.05 to 0.228)	117 (29 to 185)	0.174 (0.04 to 0.275)	0.129 (-0.322 to 0.654)	0.63 (0.47 to 0.79)
Iran (Islamic Republic of)	512 (262 to 670)	2.016 (1.03 to 2.64)	107 (76 to 127)	0.532 (0.377 to 0.632)	-0.79 (-0.847 to -0.672)	-3.05 (-3.48 to -2.63)
Iraq	118 (80 to 153)	1.437 (0.974 to 1.855)	81 (59 to 106)	0.6 (0.44 to 0.788)	-0.315 (-0.536 to -0.034)	-2.54 (-2.71 to -2.38)
Ireland	4 (3 to 4)	0.376 (0.343 to 0.419)	2 (2 to 3)	0.229 (0.195 to 0.274)	-0.382 (-0.481 to -0.244)	-0.86 (-1.36 to -0.36)
Israel	6 (5 to 6)	0.362 (0.327 to 0.4)	7 (6 to 8)	0.25 (0.211 to 0.303)	0.184 (-0.026 to 0.488)	-0.32 (-0.7 to 0.07)
Italy	21 (20 to 22)	0.228 (0.218 to 0.241)	22 (18 to 26)	0.288 (0.242 to 0.336)	0.04 (-0.12 to 0.197)	1.25 (0.91 to 1.59)
Jamaica	10 (9 to 11)	1.188 (1.033 to 1.37)	3 (2 to 4)	0.529 (0.417 to 0.673)	-0.689 (-0.761 to -0.594)	-2.52 (-2.94 to -2.09)
Japan	41 (40 to 43)	0.18 (0.174 to 0.185)	38 (34 to 41)	0.245 (0.222 to 0.267)	-0.089 (-0.18 to -0.003)	0.11 (-0.4 to 0.62)
Jordan	19 (14 to 24)	1.158 (0.849 to 1.459)	17 (14 to 22)	0.48 (0.375 to 0.61)	-0.077 (-0.325 to 0.243)	-2.96 (-3.2 to -2.72)
Kazakhstan	36 (32 to 41)	0.693 (0.608 to 0.78)	38 (30 to 47)	0.698 (0.548 to 0.871)	0.052 (-0.164 to 0.351)	-0.62 (-1.13 to -0.11)
Kenya	197 (134 to 250)	1.763 (1.203 to 2.236)	288 (206 to 383)	1.541 (1.102 to 2.05)	0.46 (0.067 to 0.936)	0.33 (0.03 to 0.62)
Kiribati	0 (0 to 0)	0.782 (0.433 to 1.276)	0 (0 to 0)	0.648 (0.41 to 0.944)	0.179 (-0.238 to 0.846)	-0.58 (-0.75 to -0.42)
Kuwait	4 (4 to 5)	0.806 (0.675 to 0.93)	4 (3 to 5)	0.459 (0.378 to 0.555)	-0.132 (-0.347 to 0.165)	-2.49 (-2.93 to -2.05)
Kyrgyzstan	28 (24 to 32)	1.662 (1.436 to 1.913)	24 (20 to 27)	1.036 (0.89 to 1.191)	-0.155 (-0.31 to 0.033)	-2.17 (-2.75 to -1.6)
Lao People's Democratic Republic	10 (6 to 17)	0.555 (0.335 to 0.94)	9 (6 to 14)	0.372 (0.248 to 0.603)	-0.165 (-0.486 to 0.321)	-1.27 (-1.34 to -1.21)
Latvia	6 (5 to 7)	0.974 (0.8 to 1.165)	0 (0 to 1)	0.143 (0.106 to 0.194)	-0.923 (-0.947 to -0.886)	-6.17 (-6.85 to -5.48)
Lebanon	17 (12 to 25)	1.636 (1.128 to 2.359)	9 (6 to 12)	0.698 (0.506 to 0.955)	-0.478 (-0.66 to -0.181)	-2.59 (-2.95 to -2.24)
Lesotho	7 (5 to 9)	0.999 (0.736 to 1.372)	8 (5 to 10)	1.204 (0.845 to 1.613)	0.114 (-0.273 to 0.71)	1.21 (0.99 to 1.42)
Liberia	10 (7 to 15)	0.914 (0.584 to 1.332)	10 (6 to 15)	0.474 (0.291 to 0.701)	0.004 (-0.396 to 0.799)	-1.77 (-2.2 to -1.33)
Libya	22 (14 to 31)	1.24 (0.746 to 1.726)	11 (7 to 17)	0.749 (0.451 to 1.136)	-0.502 (-0.66 to -0.267)	-1.39 (-1.48 to -1.3)
Lithuania	8 (7 to 10)	1.018 (0.89 to 1.173)	1 (1 to 2)	0.333 (0.226 to 0.458)	-0.839 (-0.89 to -0.771)	-4.99 (-5.71 to -4.27)
Luxembourg	0 (0 to 0)	0.533 (0.468 to 0.605)	0 (0 to 0)	0.287 (0.227 to 0.351)	-0.175 (-0.377 to 0.045)	-2.19 (-2.53 to -1.84)
Madagascar	122 (88 to 170)	2.238 (1.619 to 3.118)	194 (137 to 278)	1.651 (1.169 to 2.368)	0.586 (0.074 to 1.395)	-0.77 (-0.87 to -0.67)
Malawi	151 (105 to 204)	3.319 (2.305 to 4.489)	174 (121 to 241)	2.142 (1.491 to 2.97)	0.152 (-0.259 to 0.877)	-1.21 (-1.38 to -1.04)
Malaysia	26 (20 to 34)	0.402 (0.304 to 0.52)	15 (12 to 20)	0.204 (0.161 to 0.265)	-0.414 (-0.581 to -0.198)	-1.97 (-2.16 to -1.78)
Maldives	1 (1 to 1)	0.944 (0.569 to 1.293)	1 (0 to 1)	0.601 (0.392 to 0.832)	-0.393 (-0.598 to -0.069)	-1.18 (-1.51 to -0.84)
Mali	32 (20 to 48)	0.774 (0.476 to 1.163)	70 (44 to 101)	0.61 (0.379 to 0.871)	1.201 (0.433 to 2.322)	-0.5 (-0.65 to -0.36)
Malta	0 (0 to 0)	0.19 (0.149 to 0.221)	0 (0 to 0)	0.282 (0.215 to 0.372)	0.085 (-0.208 to 0.463)	1.24 (0.93 to 1.55)
Marshall Islands	0 (0 to 0)	0.298 (0.211 to 0.43)	0 (0 to 0)	0.361 (0.242 to 0.527)	-0.037 (-0.384 to 0.561)	0.44 (0.08 to 0.81)
Mauritania	5 (3 to 7)	0.534 (0.373 to 0.749)	8 (5 to 10)	0.407 (0.279 to 0.554)	0.528 (-0.011 to 1.343)	-0.86 (-1.1 to -0.62)
Mauritius	4 (4 to 5)	1.258 (1.134 to 1.413)	2 (2 to 3)	1.198 (0.93 to 1.486)	-0.402 (-0.535 to -0.238)	-0.4 (-1.12 to 0.32)
Mexico	558 (508 to 625)	1.671 (1.521 to 1.87)	296 (228 to 381)	0.924 (0.712 to 1.189)	-0.469 (-0.615 to -0.304)	-1.49 (-1.66 to -1.33)
Micronesia (Federated States of)	0 (0 to 0)	0.405 (0.284 to 0.556)	0 (0 to 0)	0.325 (0.222 to 0.465)	-0.465 (-0.642 to -0.143)	-0.63 (-0.76 to -0.5)
Monaco	0 (0 to 0)	0.13 (0.09 to 0.205)	0 (0 to 0)	0.187 (0.137 to 0.257)	0.987 (0.42 to 1.899)	-0.74 (-1.41 to -0.06)
Mongolia	16 (12 to 21)	1.828 (1.315 to 2.369)	7 (5 to 10)	0.607 (0.42 to 0.954)	-0.599 (-0.746 to -0.281)	-3.55 (-3.81 to -3.28)
Montenegro	1 (1 to 1)	0.447 (0.319 to 0.601)	0 (0 to 0)	0.236 (0.16 to 0.356)	-0.637 (-0.766 to -0.439)	-2.21 (-2.64 to -1.77)
Morocco	187 (125 to 256)	1.908 (1.279 to 2.621)	92 (57 to 130)	0.941 (0.58 to 1.328)	-0.507 (-0.69 to -0.276)	-1.48 (-1.82 to -1.14)
Mozambique	225 (162 to 320)	3.629 (2.613 to 5.152)	333 (231 to 474)	2.335 (1.617 to 3.32)	0.48 (-0.044 to 1.358)	-1.12 (-1.29 to -0.95)
Myanmar	38 (24 to 66)	0.257 (0.162 to 0.444)	32 (20 to 58)	0.208 (0.131 to 0.373)	-0.146 (-0.456 to 0.219)	-0.73 (-0.82 to -0.64)
Namibia	6 (4 to 8)	0.957 (0.727 to 1.272)	8 (6 to 12)	1.025 (0.731 to 1.428)	0.471 (0.009 to 1.164)	0.46 (0.31 to 0.61)
Nauru	0 (0 to 0)	0.41 (0.268 to 0.579)	0 (0 to 0)	0.416 (0.28 to 0.585)	-0.042 (-0.353 to 0.503)	0.04 (-0.33 to 0.41)
Nepal	235 (143 to 401)	2.791 (1.694 to 4.759)	129 (83 to 229)	1.402 (0.897 to 2.483)	-0.45 (-0.719 to 0.048)	-2.03 (-2.2 to -1.86)
Netherlands	12 (10 to 13)	0.425 (0.377 to 0.475)	10 (8 to 12)	0.369 (0.313 to 0.435)	-0.147 (-0.311 to 0.062)	-0.09 (-0.35 to 0.16)
New Zealand	6 (6 to 7)	0.795 (0.717 to 0.887)	3 (3 to 4)	0.354 (0.311 to 0.406)	-0.453 (-0.536 to -0.363)	-2.38 (-2.62 to -2.14)
Nicaragua	32 (18 to 41)	1.739 (0.982 to 2.248)	11 (8 to 17)	0.57 (0.41 to 0.86)	-0.644 (-0.774 to -0.204)	-3.1 (-3.29 to -2.9)
Niger	36 (21 to 55)	0.897 (0.524 to 1.344)	79 (44 to 124)	0.618 (0.35 to 0.973)	1.166 (0.259 to 2.811)	-1.18 (-1.37 to -0.99)
Nigeria	225 (156 to 311)	0.574 (0.398 to 0.79)	553 (314 to 740)	0.544 (0.309 to 0.728)	1.463 (0.547 to 2.428)	0.15 (0.04 to 0.26)
Niue	0 (0 to 0)	0.338 (0.242 to 0.477)	0 (0 to 0)	1.148 (0.88 to 1.437)	0.631 (0.162 to 1.416)	1.09 (0.04 to 2.15)
North Macedonia	5 (4 to 6)	0.929 (0.718 to 1.18)	1 (1 to 2)	0.372 (0.276 to 0.51)	-0.751 (-0.83 to -0.641)	-2.41 (-2.68 to -2.13)

Northern Mariana Islands	0 (0 to 0)	0.045 (0.024 to 0.105)	0 (0 to 0)	0.09 (0.07 to 0.129)	0.931 (-0.055 to 2.795)	3.18 (2.37 to 3.99)
Norway	5 (4 to 5)	0.592 (0.55 to 0.635)	3 (2 to 3)	0.293 (0.257 to 0.341)	-0.428 (-0.5 to -0.328)	-1.5 (-1.98 to -1.01)
Oman	3 (2 to 5)	0.405 (0.282 to 0.551)	2 (2 to 3)	0.193 (0.14 to 0.242)	-0.308 (-0.52 to 0.03)	-1.58 (-1.98 to -1.17)
Pakistan	1119 (848 to 1461)	2.27 (1.721 to 2.967)	1858 (1421 to 2508)	2.174 (1.663 to 2.936)	0.659 (0.278 to 1.241)	0.41 (0.23 to 0.59)
Palau	0 (0 to 0)	0.442 (0.315 to 0.601)	0 (0 to 0)	0.339 (0.241 to 0.46)	-0.451 (-0.611 to -0.207)	-0.47 (-0.7 to -0.25)
Palestine	22 (15 to 29)	2.234 (1.582 to 2.976)	20 (16 to 27)	1.077 (0.831 to 1.426)	-0.071 (-0.335 to 0.352)	-1.95 (-2.16 to -1.74)
Panama	9 (7 to 10)	1.041 (0.887 to 1.225)	7 (5 to 9)	0.593 (0.47 to 0.74)	-0.212 (-0.403 to 0.032)	-1.51 (-1.64 to -1.39)
Papua New Guinea	8 (4 to 11)	0.445 (0.255 to 0.637)	21 (14 to 30)	0.527 (0.346 to 0.758)	1.729 (0.937 to 3.061)	0.71 (0.52 to 0.91)
Paraguay	16 (12 to 20)	0.948 (0.723 to 1.201)	14 (10 to 19)	0.703 (0.505 to 0.964)	-0.109 (-0.378 to 0.294)	-0.46 (-0.65 to -0.28)
Peru	102 (66 to 127)	1.228 (0.794 to 1.531)	36 (25 to 53)	0.374 (0.26 to 0.552)	-0.65 (-0.78 to -0.29)	-3.86 (-4.12 to -3.59)
Philippines	71 (38 to 92)	0.281 (0.152 to 0.363)	76 (40 to 101)	0.223 (0.116 to 0.298)	0.068 (-0.234 to 0.381)	-0.32 (-0.45 to -0.18)
Poland	34 (33 to 36)	0.354 (0.34 to 0.373)	18 (15 to 21)	0.311 (0.249 to 0.357)	-0.461 (-0.566 to -0.378)	0.41 (0.15 to 0.67)
Portugal	14 (12 to 15)	0.64 (0.58 to 0.708)	6 (5 to 7)	0.43 (0.358 to 0.52)	-0.567 (-0.646 to -0.469)	-1.72 (-2.04 to -1.4)
Puerto Rico	5 (5 to 6)	0.526 (0.475 to 0.581)	1 (0 to 1)	0.13 (0.101 to 0.156)	-0.893 (-0.915 to -0.865)	-4.24 (-4.55 to -3.93)
Qatar	1 (1 to 1)	0.749 (0.541 to 1.012)	1 (1 to 2)	0.23 (0.16 to 0.349)	0.197 (-0.249 to 0.875)	-3.39 (-3.65 to -3.13)
Republic of Korea	110 (88 to 150)	0.967 (0.778 to 1.321)	24 (19 to 30)	0.388 (0.306 to 0.496)	-0.786 (-0.853 to -0.715)	-3 (-3.17 to -2.84)
Republic of Moldova	19 (17 to 24)	1.577 (1.337 to 1.904)	3 (2 to 4)	0.568 (0.408 to 0.781)	-0.848 (-0.893 to -0.782)	-4.01 (-4.93 to -3.08)
Romania	64 (58 to 69)	1.142 (1.033 to 1.248)	19 (15 to 25)	0.64 (0.51 to 0.815)	-0.697 (-0.764 to -0.605)	-1.41 (-1.63 to -1.18)
Russian Federation	125 (121 to 129)	0.36 (0.35 to 0.371)	32 (28 to 36)	0.124 (0.109 to 0.136)	-0.74 (-0.768 to -0.717)	-4.61 (-5.5 to -3.71)
Rwanda	102 (73 to 140)	3.02 (2.143 to 4.121)	95 (70 to 130)	1.912 (1.409 to 2.62)	-0.073 (-0.406 to 0.395)	-1.66 (-1.89 to -1.43)
Saint Kitts and Nevis	0 (0 to 0)	1.432 (1.257 to 1.62)	0 (0 to 0)	0.764 (0.629 to 0.931)	-0.628 (-0.704 to -0.531)	-2.27 (-2.56 to -1.97)
Saint Lucia	1 (1 to 1)	1.286 (1.07 to 1.507)	0 (0 to 0)	0.768 (0.597 to 0.98)	-0.656 (-0.749 to -0.54)	-1.98 (-2.16 to -1.79)
Saint Vincent and the Grenadines	1 (0 to 1)	1.408 (1.157 to 1.684)	0 (0 to 0)	0.969 (0.75 to 1.197)	-0.582 (-0.69 to -0.456)	-1.41 (-1.85 to -0.97)
Samoa	0 (0 to 0)	0.322 (0.224 to 0.463)	0 (0 to 0)	0.259 (0.168 to 0.374)	-0.1 (-0.398 to 0.368)	-0.67 (-0.75 to -0.59)
San Marino	0 (0 to 0)	0.1 (0.06 to 0.138)	0 (0 to 0)	0.05 (0.03 to 0.06)	-0.504 (-0.678 to -0.249)	-1.85 (-2.03 to -1.66)
Sao Tome and Principe	0 (0 to 1)	0.698 (0.475 to 0.948)	0 (0 to 0)	0.415 (0.258 to 0.603)	-0.183 (-0.503 to 0.324)	-1.21 (-1.7 to -0.72)
Saudi Arabia	99 (68 to 142)	1.508 (1.04 to 2.164)	36 (26 to 55)	0.481 (0.341 to 0.724)	-0.632 (-0.786 to -0.385)	-3.82 (-3.89 to -3.74)
Senegal	28 (19 to 38)	0.767 (0.532 to 1.036)	38 (27 to 51)	0.59 (0.42 to 0.8)	0.34 (-0.072 to 0.941)	-0.25 (-0.54 to 0.04)
Serbia	21 (15 to 28)	0.947 (0.669 to 1.298)	5 (3 to 7)	0.366 (0.25 to 0.537)	-0.764 (-0.858 to -0.628)	-3.34 (-3.71 to -2.97)
Seychelles	0 (0 to 0)	0.462 (0.342 to 0.605)	0 (0 to 0)	0.197 (0.123 to 0.292)	-0.579 (-0.733 to -0.311)	-1.18 (-1.84 to -0.52)
Sierra Leone	15 (10 to 23)	0.854 (0.529 to 1.252)	22 (14 to 33)	0.614 (0.385 to 0.91)	0.419 (-0.085 to 1.337)	-1.03 (-1.22 to -0.84)
Singapore	2 (2 to 2)	0.283 (0.253 to 0.32)	2 (1 to 2)	0.215 (0.174 to 0.267)	-0.05 (-0.248 to 0.205)	-1.02 (-1.37 to -0.67)
Slovakia	10 (8 to 14)	0.776 (0.593 to 1.03)	4 (3 to 5)	0.479 (0.349 to 0.629)	-0.601 (-0.713 to -0.43)	-1.2 (-1.35 to -1.05)
Slovenia	2 (2 to 2)	0.428 (0.376 to 0.486)	1 (1 to 1)	0.218 (0.167 to 0.287)	-0.615 (-0.716 to -0.484)	-1.96 (-2.14 to -1.77)
Solomon Islands	1 (0 to 1)	0.426 (0.265 to 0.654)	1 (1 to 2)	0.441 (0.292 to 0.655)	0.728 (0.274 to 1.541)	0.18 (-0.06 to 0.43)
Somalia	108 (72 to 159)	2.78 (1.837 to 4.08)	229 (128 to 383)	2.212 (1.238 to 3.708)	1.107 (0.272 to 2.241)	-0.46 (-0.75 to -0.17)
South Africa	143 (101 to 173)	1.049 (0.74 to 1.268)	119 (90 to 144)	0.785 (0.592 to 0.946)	-0.165 (-0.325 to 0.038)	-0.9 (-0.98 to -0.81)
South Sudan	66 (44 to 97)	2.531 (1.689 to 3.694)	118 (83 to 162)	2.737 (1.935 to 3.774)	0.77 (0.232 to 1.673)	0.37 (-0.03 to 0.78)
Spain	20 (18 to 22)	0.253 (0.228 to 0.282)	16 (14 to 19)	0.246 (0.21 to 0.288)	-0.198 (-0.348 to -0.039)	-0.11 (-0.35 to 0.13)
Sri Lanka	65 (49 to 80)	1.166 (0.879 to 1.448)	22 (15 to 32)	0.426 (0.29 to 0.633)	-0.663 (-0.776 to -0.477)	-3.52 (-4.01 to -3.04)
Sudan	261 (122 to 454)	2.941 (1.369 to 5.1)	244 (148 to 352)	1.472 (0.895 to 2.124)	-0.066 (-0.441 to 0.567)	-1.9 (-2.11 to -1.69)
Suriname	2 (1 to 3)	1.575 (0.825 to 2.078)	1 (1 to 2)	1.019 (0.734 to 1.403)	-0.288 (-0.553 to 0.433)	-1.3 (-1.55 to -1.04)
Sweden	4 (4 to 5)	0.266 (0.241 to 0.293)	3 (2 to 3)	0.149 (0.129 to 0.173)	-0.338 (-0.454 to -0.204)	-1.35 (-1.75 to -0.95)
Switzerland	4 (4 to 5)	0.372 (0.307 to 0.449)	4 (3 to 4)	0.282 (0.237 to 0.328)	-0.126 (-0.32 to 0.133)	-0.78 (-0.92 to -0.63)
Syrian Arab Republic	56 (38 to 77)	0.948 (0.63 to 1.307)	25 (18 to 35)	0.694 (0.492 to 0.95)	-0.547 (-0.725 to -0.249)	-0.96 (-1.39 to -0.53)
Taiwan (Province of China)	22 (21 to 25)	0.408 (0.372 to 0.448)	9 (8 to 11)	0.302 (0.26 to 0.36)	-0.604 (-0.667 to -0.525)	-1.04 (-1.28 to -0.79)
Tajikistan	71 (51 to 88)	3.066 (2.188 to 3.81)	99 (66 to 146)	2.766 (1.839 to 4.078)	0.392 (-0.068 to 1.052)	-0.63 (-0.89 to -0.37)
Thailand	109 (80 to 146)	0.648 (0.473 to 0.869)	42 (31 to 51)	0.426 (0.318 to 0.524)	-0.619 (-0.727 to -0.461)	-1.46 (-1.66 to -1.26)
Timor-Leste	2 (1 to 2)	0.517 (0.336 to 0.748)	2 (1 to 3)	0.338 (0.231 to 0.527)	0.023 (-0.331 to 0.537)	-1.49 (-1.71 to -1.27)
Togo	10 (7 to 14)	0.587 (0.408 to 0.8)	15 (9 to 21)	0.444 (0.282 to 0.634)	0.421 (-0.062 to 1.177)	-0.68 (-0.8 to -0.57)
Tokelau	0 (0 to 0)	0.376 (0.257 to 0.54)	0 (0 to 0)	1.991 (1.405 to 2.757)	2.446 (1.377 to 4.14)	1.17 (-0.45 to 2.83)
Tonga	0 (0 to 0)	0.26 (0.184 to 0.356)	0 (0 to 0)	0.248 (0.175 to 0.365)	-0.106 (-0.4 to 0.417)	-0.24 (-0.51 to 0.03)
Trinidad and Tobago	5 (5 to 6)	1.307 (1.136 to 1.491)	2 (1 to 2)	0.576 (0.445 to 0.733)	-0.704 (-0.781 to -0.605)	-2.75 (-3.01 to -2.49)
Tunisia	56 (32 to 77)	1.802 (1.039 to 2.477)	18 (12 to 25)	0.637 (0.425 to 0.891)	-0.685 (-0.794 to -0.516)	-3.09 (-3.18 to -3)
Türkiye	633 (402 to 894)	3.091 (1.962 to 4.361)	224 (168 to 285)	1.209 (0.906 to 1.538)	-0.647 (-0.771 to -0.468)	-2.66 (-2.95 to -2.37)
Turkmenistan	19 (16 to 23)	1.295 (1.087 to 1.523)	21 (11 to 30)	1.364 (0.718 to 1.998)	0.069 (-0.484 to 0.738)	-0.41 (-0.92 to 0.09)
Tuvalu	0 (0 to 0)	0.452 (0.314 to 0.642)	0 (0 to 0)	0.324 (0.227 to 0.461)	-0.231 (-0.509 to 0.219)	-0.87 (-0.98 to -0.77)
Uganda	164 (113 to 229)	1.947 (1.348 to 2.715)	335 (241 to 470)	1.687 (1.215 to 2.368)	1.042 (0.412 to 2.071)	-0.2 (-0.35 to -0.04)
Ukraine	56 (49 to 65)	0.496 (0.43 to 0.569)	15 (12 to 19)	0.23 (0.187 to 0.3)	-0.741 (-0.799 to -0.658)	-4.05 (-4.74 to -3.35)
United Arab Emirates	6 (4 to 8)	0.982 (0.696 to 1.433)	4 (3 to 6)	0.292 (0.22 to 0.416)	-0.324 (-0.522 to -0.07)	-2.98 (-3.32 to -2.65)
United Kingdom	65 (63 to 67)	0.598 (0.581 to 0.614)	34 (28 to 38)	0.29 (0.237 to 0.325)	-0.477 (-0.572 to -0.411)	-1.85 (-2.33 to -1.37)
United Republic of Tanzania	402 (289 to 510)	3.327 (2.389 to 4.225)	625 (444 to 849)	2.56 (1.818 to 3.478)	0.555 (0.124 to 1.231)	-0.26 (-0.47 to -0.04)
United States of America	116 (113 to 119)	0.207 (0.202 to 0.213)	145 (133 to 156)	0.24 (0.224 to 0.263)	0.25 (0.148 to 0.361)	1.17 (0.69 to 1.65)
United States Virgin Islands	0 (0 to 0)	0.525 (0.379 to 0.68)	0 (0 to 0)	0.211 (0.129 to 0.325)	-0.832 (-0.903 to -0.728)	-2.17 (-2.64 to -1.7)
Uruguay	6 (5 to 6)	0.708 (0.647 to 0.78)	3 (2 to 4)	0.46 (0.365 to 0.58)	-0.476 (-0.593 to -0.334)	-1.16 (-1.46 to -0.87)
Uzbekistan	132 (115 to 151)	1.543 (1.34 to 1.77)	181 (146 to 226)	1.794 (1.452 to 2.244)	0.372 (0.042 to 0.819)	0.59 (0.24 to 0.93)
Vanuatu	0 (0 to 0)	0.325 (0.197 to 0.491)	0 (0 to 1)	0.327 (0.223 to 0.485)	0.726 (0.162 to 1.754)	0.28 (-0.05 to 0.61)

Venezuela (Bolivarian Republic of)	71 (65 to 77)	1.002 (0.923 to 1.08)	54 (41 to 69)	0.822 (0.622 to 1.045)	-0.234 (-0.426 to -0.018)	-0.23 (-0.71 to 0.25)
Viet Nam	9 (2 to 38)	0.04 (0.01 to 0.143)	7 (1 to 28)	0.03 (0.01 to 0.113)	-0.265 (-0.487 to 0.08)	-0.63 (-1.18 to -0.08)
Yemen	161 (75 to 257)	2.265 (1.058 to 3.619)	199 (118 to 280)	1.444 (0.857 to 2.027)	0.239 (-0.256 to 1.038)	-1.33 (-1.5 to -1.16)
Zambia	124 (82 to 166)	3.294 (2.172 to 4.418)	207 (129 to 297)	2.503 (1.558 to 3.59)	0.674 (0.06 to 1.829)	-0.5 (-0.66 to -0.33)
Zimbabwe	49 (38 to 65)	1.018 (0.779 to 1.344)	87 (59 to 124)	1.376 (0.937 to 1.967)	0.766 (0.163 to 1.511)	1.79 (1.23 to 2.36)

Abbreviations: EAPC, estimated annual percentage change; SDI, Sociodemographic Index; UI, uncertainty interval.

[a] EAPC is expressed as 95% Cls.

Supplementary Table 5. DALYs of Childhood Idiopathic Epilepsy at the National Level

Location	1990		2021		1990-2021	
	DALYs cases	DALYs rate	DALYs cases	DALYs rate	Cases change	EAPC[a]
Afghanistan	22558 (11735 to 36278)	523.633 (272.401 to 842.099)	45540 (27354 to 67924)	320.686 (192.625 to 478.318)	1.019 (0.161 to 2.641)	-1.29 (-1.43 to -1.16)
Albania	4010 (2085 to 6282)	358.91 (186.597 to 562.249)	1036 (482 to 1798)	233.611 (108.527 to 405.257)	-0.742 (-0.872 to -0.478)	-1.68 (-1.87 to -1.49)
Algeria	35877 (18881 to 59406)	334.531 (176.049 to 553.918)	24056 (12495 to 41772)	180.849 (93.932 to 314.035)	-0.329 (-0.697 to 0.368)	-1.77 (-1.84 to -1.69)
American Samoa	24 (9 to 47)	126.325 (46.796 to 245.027)	19 (8 to 36)	134.392 (56.24 to 254.474)	-0.207 (-0.706 to 1.122)	0.53 (0.3 to 0.75)
Andorra	12 (5 to 23)	126.448 (52.68 to 241.104)	9 (3 to 21)	88.879 (29.488 to 204.488)	-0.248 (-0.724 to 0.946)	-1.11 (-1.2 to -1.02)
Angola	18565 (9736 to 31039)	393.766 (206.491 to 658.328)	46536 (21444 to 78198)	305.247 (140.66 to 512.924)	1.507 (0.06 to 4.322)	-0.61 (-0.75 to -0.47)
Antigua and Barbuda	45 (22 to 80)	246.888 (122.666 to 439.525)	37 (19 to 66)	220.878 (110.811 to 388.771)	-0.169 (-0.641 to 0.93)	-0.67 (-0.84 to -0.5)
Argentina	10625 (4782 to 18440)	104.831 (47.183 to 181.942)	11165 (4671 to 21866)	109.64 (45.873 to 214.725)	0.051 (-0.56 to 1.583)	0.44 (0.24 to 0.64)
Armenia	1644 (750 to 3046)	157.597 (71.871 to 291.983)	711 (261 to 1494)	120.001 (44.1 to 252.265)	-0.568 (-0.856 to 0.205)	-1.18 (-1.28 to -1.08)
Australia	4202 (2117 to 7878)	110.983 (55.928 to 208.089)	4014 (1635 to 8437)	84.517 (34.429 to 177.645)	-0.045 (-0.641 to 1.429)	-0.98 (-1.05 to -0.91)
Austria	1426 (559 to 3032)	105.79 (41.478 to 224.901)	1207 (439 to 2532)	93.095 (33.859 to 195.189)	-0.153 (-0.69 to 1.377)	-0.34 (-0.39 to -0.3)
Azerbaijan	8047 (4617 to 13147)	331.57 (190.225 to 541.711)	6174 (3273 to 10301)	261.541 (138.672 to 436.379)	-0.233 (-0.643 to 0.615)	-1 (-1.23 to -0.77)
Bahamas	160 (68 to 295)	198.32 (84.442 to 365.805)	126 (42 to 249)	154.897 (51.333 to 306.561)	-0.214 (-0.748 to 1.139)	-0.99 (-1.06 to -0.93)
Bahrain	485 (263 to 830)	297.392 (161.375 to 508.5)	515 (226 to 992)	173.425 (76.115 to 334.431)	0.06 (-0.546 to 1.37)	-1.9 (-1.97 to -1.84)
Bangladesh	97524 (49016 to 155987)	199.389 (100.214 to 318.916)	55803 (26522 to 102455)	121.932 (57.952 to 223.866)	-0.428 (-0.742 to 0.312)	-1.92 (-2.06 to -1.78)
Barbados	128 (63 to 225)	205.288 (100.345 to 361.526)	69 (29 to 135)	147.457 (62.469 to 286.478)	-0.458 (-0.776 to 0.399)	-1.03 (-1.1 to -0.96)
Belarus	3229 (1703 to 5826)	134.344 (70.825 to 242.379)	1179 (452 to 2481)	74.7 (28.627 to 157.228)	-0.635 (-0.852 to -0.159)	-2.22 (-2.34 to -2.1)
Belgium	2486 (1049 to 4854)	137.636 (58.079 to 268.769)	2420 (1077 to 4971)	126.542 (56.324 to 259.984)	-0.027 (-0.627 to 1.336)	-0.26 (-0.33 to -0.19)
Belize	205 (126 to 322)	250.232 (153.815 to 393.729)	183 (72 to 343)	148.483 (58.575 to 278.631)	-0.108 (-0.638 to 0.805)	-1.85 (-1.95 to -1.75)
Benin	6088 (2251 to 11889)	251.39 (92.954 to 490.918)	12687 (5073 to 24276)	208.651 (83.431 to 399.255)	1.084 (-0.307 to 4.548)	-0.58 (-0.69 to -0.47)
Bermuda	21 (8 to 41)	173.158 (63.668 to 345.959)	11 (4 to 23)	126.704 (41.719 to 272.548)	-0.482 (-0.817 to 0.582)	-0.92 (-1.04 to -0.8)
Bhutan	687 (349 to 1076)	261.883 (133.206 to 410.5)	464 (248 to 735)	247.856 (132.55 to 392.438)	-0.324 (-0.67 to 0.478)	-0.46 (-0.76 to -0.16)
Bolivia (Plurinational State of)	9955 (5529 to 15850)	370.661 (205.875 to 590.145)	6743 (3495 to 11560)	193.393 (100.243 to 331.558)	-0.323 (-0.663 to 0.397)	-2.06 (-2.11 to -2.02)
Bosnia and Herzegovina	2073 (1081 to 3508)	189.209 (98.63 to 320.153)	672 (291 to 1247)	137.055 (59.34 to 254.205)	-0.676 (-0.867 to -0.245)	-1.36 (-1.51 to -1.21)
Botswana	1301 (713 to 2192)	220.31 (120.698 to 371.203)	1796 (903 to 3049)	257.226 (129.33 to 436.647)	0.381 (-0.318 to 1.786)	0.47 (0.39 to 0.55)
Brazil	111845 (75117 to 169795)	215.316 (144.611 to 326.878)	75246 (51116 to 112031)	156.159 (106.082 to 232.501)	-0.327 (-0.511 to -0.072)	-0.61 (-0.91 to -0.31)
Brunei Darussalam	195 (92 to 343)	215.225 (101.274 to 378.734)	147 (68 to 264)	155.272 (72.229 to 279.199)	-0.247 (-0.662 to 0.709)	-0.83 (-0.96 to -0.7)
Bulgaria	3242 (1618 to 5735)	186.716 (93.168 to 330.321)	1529 (690 to 2821)	156.641 (70.692 to 289.052)	-0.528 (-0.804 to 0.083)	-0.81 (-0.92 to -0.7)
Burkina Faso	10136 (4060 to 19981)	214.782 (86.033 to 423.414)	21381 (8312 to 39886)	206.14 (80.133 to 384.544)	1.109 (-0.257 to 4.75)	-0.07 (-0.11 to -0.02)
Burundi	10821 (6427 to 16538)	412.829 (245.215 to 630.947)	15924 (8301 to 26427)	272.015 (141.792 to 451.424)	0.472 (-0.237 to 1.888)	-1.39 (-1.58 to -1.2)
Cabo Verde	275 (97 to 580)	175.063 (61.663 to 368.959)	229 (77 to 450)	159.75 (54.003 to 314.452)	-0.169 (-0.742 to 1.632)	-0.45 (-0.53 to -0.38)
Cambodia	7042 (3021 to 13534)	151.08 (64.81 to 290.374)	6345 (2704 to 11501)	124.014 (52.841 to 224.784)	-0.099 (-0.645 to 1.242)	-0.46 (-0.57 to -0.36)
Cameroon	10258 (4006 to 19078)	210.107 (82.05 to 390.77)	24700 (9361 to 44008)	183.41 (69.514 to 326.784)	1.408 (-0.172 to 5.869)	-0.28 (-0.37 to -0.19)

Canada	4469 (2327 to 8295)	77.694 (40.456 to 144.21)	4417 (1796 to 9131)	71.572 (29.094 to 147.952)	-0.012 (-0.618 to 1.352)	0.16 (0.02 to 0.3)
Central African Republic	4234 (2267 to 6736)	346.26 (185.375 to 550.931)	7427 (4453 to 12023)	325.188 (194.97 to 526.437)	0.754 (-0.059 to 2.531)	-0.1 (-0.17 to -0.03)
Chad	5606 (1911 to 11326)	191.58 (65.29 to 387.037)	18405 (7151 to 35847)	204.163 (79.329 to 397.647)	2.283 (0.236 to 7.937)	0.31 (0.25 to 0.38)
Chile	8149 (4034 to 14603)	205.169 (101.565 to 367.655)	5307 (2253 to 10525)	145.327 (61.7 to 288.224)	-0.349 (-0.778 to 0.664)	-0.88 (-1.02 to -0.73)
China	770516 (574244 to 939901)	242.014 (180.366 to 295.217)	250196 (175679 to 368855)	96.366 (67.665 to 142.069)	-0.675 (-0.767 to -0.534)	-3.24 (-3.32 to -3.16)
Colombia	32960 (15503 to 56080)	282.599 (132.922 to 480.839)	19627 (8110 to 37285)	184.932 (76.42 to 351.311)	-0.405 (-0.781 to 0.449)	-1.05 (-1.2 to -0.89)
Comoros	848 (491 to 1316)	398.739 (230.69 to 618.873)	782 (460 to 1220)	325.831 (191.667 to 508.041)	-0.077 (-0.506 to 0.775)	-0.84 (-0.98 to -0.69)
Congo	3521 (1727 to 6361)	334.376 (164.003 to 604.06)	4964 (2326 to 8649)	257.323 (120.583 to 448.282)	0.41 (-0.436 to 2.272)	-0.87 (-0.98 to -0.75)
Cook Islands	8 (3 to 16)	125 (47.13 to 237.358)	4 (2 to 8)	112.791 (44.42 to 216.327)	-0.482 (-0.821 to 0.562)	-0.47 (-0.57 to -0.37)
Costa Rica	2141 (941 to 3957)	190.414 (83.691 to 351.997)	1592 (626 to 3096)	156.471 (61.558 to 304.301)	-0.256 (-0.707 to 0.805)	-0.64 (-0.69 to -0.59)
Côte d'Ivoire	12363 (4227 to 24247)	216.762 (74.109 to 425.138)	23473 (9182 to 46400)	202.843 (79.35 to 400.978)	0.899 (-0.344 to 4.341)	-0.37 (-0.44 to -0.3)
Croatia	1588 (725 to 3042)	160.907 (73.444 to 308.187)	753 (269 to 1598)	126.058 (44.979 to 267.564)	-0.526 (-0.826 to 0.234)	-0.71 (-0.84 to -0.59)
Cuba	3108 (1199 to 5888)	124.115 (47.887 to 235.149)	1683 (543 to 3569)	94.72 (30.547 to 200.87)	-0.458 (-0.84 to 0.494)	-0.71 (-0.81 to -0.62)
Cyprus	224 (92 to 433)	113.05 (46.558 to 218.951)	170 (57 to 363)	77.77 (25.989 to 166.074)	-0.24 (-0.788 to 1.113)	-1.26 (-1.38 to -1.14)
Czechia	3588 (1617 to 6916)	162.812 (73.394 to 313.852)	2149 (826 to 4614)	125.197 (48.13 to 268.819)	-0.401 (-0.774 to 0.61)	-1.01 (-1.11 to -0.92)
Democratic People's Republic of Korea	11596 (6164 to 18542)	194.917 (103.606 to 311.673)	5304 (2836 to 9211)	111.107 (59.403 to 192.96)	-0.543 (-0.786 to -0.097)	-1.76 (-1.82 to -1.7)
Democratic Republic of the Congo	56971 (28522 to 96511)	321.8 (161.105 to 545.146)	82704 (41468 to 137953)	217.649 (109.13 to 363.04)	0.452 (-0.327 to 2.042)	-0.87 (-1.04 to -0.71)
Denmark	886 (367 to 1775)	100.315 (41.59 to 200.941)	676 (282 to 1439)	70.796 (29.55 to 150.797)	-0.238 (-0.693 to 0.874)	-1.21 (-1.31 to -1.11)
Djibouti	642 (371 to 1053)	368.688 (213.305 to 604.566)	1344 (736 to 2128)	325.325 (178.183 to 514.991)	1.094 (0.061 to 2.91)	-0.43 (-0.66 to -0.2)
Dominica	65 (34 to 116)	261.731 (135.284 to 466.956)	41 (22 to 68)	301.428 (159.499 to 498.372)	-0.365 (-0.696 to 0.385)	0.38 (0.31 to 0.45)
Dominican Republic	6766 (3435 to 11146)	251.013 (127.436 to 413.521)	5504 (2492 to 9556)	187.295 (84.79 to 325.213)	-0.187 (-0.586 to 0.741)	-0.85 (-1.04 to -0.66)
Ecuador	12093 (6581 to 19588)	312.869 (170.256 to 506.771)	12128 (5710 to 21904)	239.178 (112.608 to 431.956)	0.003 (-0.639 to 1.301)	-1.3 (-1.47 to -1.12)
Egypt	53579 (23747 to 96087)	241.545 (107.058 to 433.181)	56925 (23865 to 108143)	154.456 (64.753 to 293.427)	0.062 (-0.585 to 1.717)	-0.86 (-1.17 to -0.55)
El Salvador	4940 (2377 to 8329)	228.91 (110.152 to 385.929)	2700 (1105 to 5171)	148.452 (60.767 to 284.294)	-0.453 (-0.794 to 0.286)	-1.42 (-1.57 to -1.26)
Equatorial Guinea	644 (341 to 1118)	327.18 (173.23 to 567.552)	1634 (621 to 2982)	279.311 (106.129 to 509.766)	1.536 (-0.087 to 5.211)	-0.38 (-0.58 to -0.19)
Eritrea	5951 (3581 to 9497)	373.84 (224.962 to 596.565)	9053 (5407 to 13803)	358.598 (214.151 to 546.74)	0.521 (-0.196 to 1.87)	-0.24 (-0.34 to -0.14)
Estonia	683 (453 to 994)	195.585 (129.621 to 284.689)	190 (74 to 397)	88.05 (34.037 to 183.826)	-0.721 (-0.889 to -0.452)	-3.01 (-3.33 to -2.68)
Eswatini	911 (471 to 1497)	236.078 (122.168 to 388.063)	1053 (550 to 1774)	255.209 (133.305 to 429.987)	0.156 (-0.479 to 1.338)	0.35 (0.21 to 0.49)
Ethiopia	96166 (63013 to 133733)	394.708 (258.631 to 548.899)	115703 (87624 to 153980)	260.884 (197.573 to 347.188)	0.203 (-0.193 to 0.835)	-1.59 (-1.71 to -1.47)
Fiji	460 (218 to 795)	163.554 (77.404 to 282.419)	477 (216 to 805)	174.989 (79.153 to 295.471)	0.036 (-0.526 to 1.468)	0.19 (0.08 to 0.29)
Finland	872 (381 to 1760)	90.388 (39.478 to 182.445)	772 (309 to 1637)	91.167 (36.465 to 193.199)	-0.115 (-0.663 to 1.504)	-0.09 (-0.19 to 0.02)
France	15837 (7242 to 29801)	135.193 (61.822 to 254.404)	14355 (6553 to 30954)	123.672 (56.452 to 266.673)	-0.094 (-0.594 to 1.229)	-0.17 (-0.26 to -0.08)
Gabon	1430 (630 to 2498)	350.847 (154.547 to 613.079)	1806 (812 to 3104)	282.625 (127.061 to 485.616)	0.263 (-0.505 to 1.887)	-0.63 (-0.74 to -0.53)
Gambia	809 (299 to 1548)	175.393 (64.75 to 335.682)	1581 (661 to 2859)	159.191 (66.498 to 287.831)	0.955 (-0.259 to 4.916)	-0.53 (-0.67 to -0.39)
Georgia	3137 (1364 to 5661)	229.184 (99.664 to 413.602)	1064 (390 to 2068)	144.547 (53.022 to 280.946)	-0.661 (-0.879 to -0.204)	-1.38 (-1.58 to -1.18)
Germany	21342 (9264 to 42780)	164.856 (71.56 to 330.454)	17347 (6816 to 39344)	144.987 (56.967 to 328.837)	-0.187 (-0.649 to 1.008)	0.2 (-0.05 to 0.45)

Ghana	11040 (3712 to 20323)	164.372 (55.263 to 302.584)	22482 (8238 to 41888)	174.508 (63.946 to 325.139)	1.036 (-0.274 to 5.38)	0.45 (0.25 to 0.64)
Greece	1658 (548 to 3844)	81.955 (27.066 to 189.978)	1160 (413 to 2578)	83.18 (29.579 to 184.84)	-0.3 (-0.763 to 1.3)	0.12 (0.07 to 0.18)
Greenland	29 (14 to 50)	203.643 (96.961 to 350.734)	17 (8 to 30)	140.594 (72.088 to 259.162)	-0.43 (-0.731 to 0.229)	-1.33 (-1.61 to -1.04)
Grenada	72 (38 to 122)	216.255 (115.231 to 363.906)	36 (16 to 70)	165.28 (73.385 to 322.947)	-0.501 (-0.776 to 0.175)	-0.74 (-0.8 to -0.68)
Guam	42 (12 to 86)	100.859 (28.915 to 206.299)	38 (12 to 78)	102.732 (32.09 to 214.386)	-0.107 (-0.713 to 2.194)	0.29 (0.17 to 0.4)
Guatemala	14808 (9329 to 22886)	364.624 (229.719 to 563.522)	12929 (7397 to 20478)	262.035 (149.926 to 415.039)	-0.127 (-0.555 to 0.713)	-0.79 (-1 to -0.57)
Guinea	6579 (2526 to 12860)	239.068 (91.778 to 467.325)	12227 (4956 to 23771)	202.24 (81.97 to 393.184)	0.859 (-0.278 to 4.184)	-0.54 (-0.59 to -0.5)
Guinea-Bissau	1187 (444 to 2294)	246.095 (92.051 to 475.497)	1702 (623 to 3055)	189.538 (69.34 to 340.106)	0.434 (-0.486 to 3.39)	-0.87 (-1.01 to -0.72)
Guyana	767 (446 to 1177)	261.075 (151.8 to 400.445)	473 (242 to 747)	221.515 (113.421 to 349.901)	-0.384 (-0.68 to 0.17)	-0.18 (-0.34 to -0.02)
Haiti	10661 (5392 to 17563)	392.935 (198.725 to 647.343)	11519 (5975 to 18306)	264.644 (137.264 to 420.55)	0.081 (-0.445 to 1.07)	-1.13 (-1.27 to -1)
Honduras	8838 (4890 to 13752)	400.013 (221.306 to 622.409)	7910 (4163 to 12624)	241.367 (127.028 to 385.198)	-0.105 (-0.543 to 0.878)	-1.67 (-1.7 to -1.64)
Hungary	3518 (1539 to 6434)	165.07 (72.194 to 301.921)	1609 (570 to 3417)	115.895 (41.07 to 246.11)	-0.543 (-0.857 to 0.225)	-1 (-1.1 to -0.9)
Iceland	51 (22 to 93)	80.587 (34.2 to 147.074)	58 (23 to 131)	85.81 (33.362 to 194.374)	0.134 (-0.575 to 1.617)	0.31 (0.17 to 0.46)
India	993798 (621904 to 1324652)	304.36 (190.464 to 405.687)	701175 (474784 to 935405)	191.369 (129.581 to 255.3)	-0.294 (-0.467 to -0.047)	-1.49 (-1.64 to -1.34)
Indonesia	70533 (41874 to 110860)	104.128 (61.818 to 163.662)	72434 (42771 to 117164)	107.647 (63.564 to 174.123)	0.027 (-0.278 to 0.488)	0.04 (-0.03 to 0.1)
Iran (Islamic Republic of)	85367 (55066 to 116135)	336.294 (216.929 to 457.502)	30899 (20147 to 47985)	153.12 (99.838 to 237.791)	-0.638 (-0.75 to -0.45)	-2.07 (-2.34 to -1.81)
Iraq	20195 (11821 to 32109)	245.196 (143.52 to 389.837)	21253 (10214 to 37087)	157.877 (75.871 to 275.497)	0.052 (-0.562 to 1.102)	-1.4 (-1.48 to -1.31)
Ireland	1086 (470 to 2222)	110.565 (47.846 to 226.169)	939 (350 to 2052)	94.15 (35.133 to 205.727)	-0.136 (-0.681 to 1.399)	-0.6 (-0.76 to -0.45)
Israel	1626 (686 to 3017)	106.06 (44.738 to 196.779)	2411 (921 to 5087)	91.743 (35.062 to 193.601)	0.483 (-0.392 to 2.64)	-0.32 (-0.4 to -0.25)
Italy	8204 (4975 to 13114)	88.89 (53.902 to 142.081)	6413 (3886 to 11411)	84.38 (51.133 to 150.147)	-0.218 (-0.405 to 0.02)	-0.33 (-0.48 to -0.18)
Jamaica	1977 (1092 to 3324)	236.74 (130.707 to 398.001)	978 (407 to 1893)	167.532 (69.749 to 324.117)	-0.505 (-0.782 to 0.058)	-1.08 (-1.23 to -0.94)
Japan	17197 (10078 to 28671)	74.48 (43.649 to 124.175)	13519 (7933 to 23563)	87.535 (51.362 to 152.568)	-0.214 (-0.375 to -0.027)	-0.01 (-0.21 to 0.19)
Jordan	3564 (1942 to 5723)	218.178 (118.898 to 350.357)	5119 (2223 to 9256)	140.911 (61.186 to 254.777)	0.437 (-0.348 to 2.162)	-1.56 (-1.65 to -1.47)
Kazakhstan	10162 (4510 to 18080)	195.568 (86.792 to 347.941)	9734 (4529 to 17990)	179.388 (83.461 to 331.521)	-0.042 (-0.599 to 1.45)	-0.83 (-1.17 to -0.49)
Kenya	33948 (24247 to 45697)	303.915 (217.066 to 409.096)	49613 (36055 to 66226)	265.81 (193.168 to 354.81)	0.461 (0.173 to 0.807)	-0.06 (-0.22 to 0.11)
Kiribati	52 (25 to 90)	174.83 (85.545 to 304.972)	63 (30 to 105)	149.314 (71.9 to 249.748)	0.215 (-0.448 to 1.937)	-0.59 (-0.72 to -0.46)
Kuwait	1165 (534 to 2224)	210.233 (96.386 to 401.224)	1329 (523 to 2695)	157.184 (61.835 to 318.763)	0.14 (-0.599 to 2.012)	-1.56 (-1.79 to -1.33)
Kyrgyzstan	4775 (2850 to 7310)	284.643 (169.904 to 435.739)	4432 (2482 to 7417)	194.848 (109.112 to 326.127)	-0.072 (-0.542 to 0.76)	-1.78 (-2.15 to -1.41)
Lao People's Democratic Republic	3063 (1198 to 5733)	166.18 (65.023 to 311.039)	3270 (1281 to 6139)	142.4 (55.772 to 267.33)	0.068 (-0.622 to 2.092)	-0.51 (-0.58 to -0.43)
Latvia	849 (513 to 1332)	149.199 (90.189 to 234.097)	236 (80 to 514)	79.588 (26.968 to 173.078)	-0.721 (-0.902 to -0.387)	-2.45 (-2.69 to -2.2)
Lebanon	2743 (1596 to 4417)	262.252 (152.619 to 422.367)	2079 (990 to 3807)	162.64 (77.46 to 297.888)	-0.242 (-0.68 to 0.616)	-1.59 (-1.76 to -1.42)
Lesotho	1263 (692 to 2163)	185.037 (101.425 to 316.9)	1456 (753 to 2384)	230.879 (119.434 to 378.151)	0.153 (-0.474 to 1.329)	0.98 (0.84 to 1.11)
Liberia	3192 (1357 to 5989)	282.444 (120.092 to 529.935)	3587 (1358 to 6640)	164.117 (62.11 to 303.74)	0.124 (-0.604 to 2.117)	-1.5 (-1.71 to -1.29)
Libya	4343 (2211 to 7220)	239.845 (122.084 to 398.687)	2359 (1159 to 3979)	158.17 (77.714 to 266.767)	-0.457 (-0.753 to 0.233)	-1.32 (-1.37 to -1.28)
Lithuania	1312 (819 to 2071)	157.988 (98.58 to 249.282)	397 (164 to 765)	97.465 (40.281 to 187.514)	-0.697 (-0.872 to -0.356)	-2.22 (-2.47 to -1.96)
Luxembourg	95 (45 to 182)	144.108 (67.459 to 274.748)	108 (42 to 227)	107.166 (41.409 to 224.339)	0.139 (-0.57 to 1.603)	-0.95 (-1.06 to -0.84)

Madagascar	19584 (11065 to 31765)	358.94 (202.8 to 582.2)	32242 (18799 to 51883)	274.789 (160.212 to 442.174)	0.646 (-0.198 to 1.988)	-0.81 (-0.89 to -0.74)
Malawi	20255 (12009 to 30345)	445.192 (263.949 to 666.974)	25408 (15426 to 39336)	312.755 (189.881 to 484.201)	0.254 (-0.288 to 1.345)	-1.07 (-1.17 to -0.97)
Malaysia	10334 (4176 to 18250)	157.21 (63.532 to 277.65)	10024 (3343 to 20363)	131.66 (43.903 to 267.451)	-0.03 (-0.693 to 1.494)	-0.59 (-0.62 to -0.55)
Maldives	261 (111 to 478)	248.547 (105.876 to 455.202)	172 (79 to 311)	171.657 (78.659 to 310.32)	-0.341 (-0.724 to 0.858)	-1.02 (-1.18 to -0.87)
Mali	6562 (2943 to 12279)	158.903 (71.27 to 297.332)	18255 (7609 to 33343)	157.703 (65.732 to 288.04)	1.782 (0.072 to 6.036)	-0.89 (-1.19 to -0.59)
Malta	75 (27 to 160)	85.638 (30.89 to 182.744)	58 (23 to 120)	90.951 (36.108 to 187.522)	-0.223 (-0.696 to 1.184)	0.06 (-0.04 to 0.17)
Marshall Islands	25 (9 to 47)	113.395 (41.095 to 213.554)	20 (8 to 36)	116.377 (46.83 to 207.558)	-0.183 (-0.714 to 1.563)	0.02 (-0.21 to 0.25)
Mauritania	1848 (697 to 3632)	199.956 (75.35 to 392.95)	2973 (1162 to 5487)	160.426 (62.688 to 296.106)	0.608 (-0.413 to 3.651)	-0.98 (-1.29 to -0.67)
Mauritius	893 (478 to 1500)	270.65 (144.786 to 454.383)	565 (302 to 952)	272.538 (145.451 to 459.18)	-0.367 (-0.705 to 0.407)	0.12 (-0.14 to 0.37)
Mexico	121148 (91199 to 161049)	362.544 (272.918 to 481.948)	75519 (50768 to 106981)	235.505 (158.318 to 333.616)	-0.377 (-0.515 to -0.224)	-1.37 (-1.42 to -1.32)
Micronesia (Federated States of)	63 (25 to 116)	136.921 (55.459 to 253.573)	36 (13 to 68)	116.079 (43.841 to 221.311)	-0.435 (-0.782 to 0.416)	-0.6 (-0.72 to -0.49)
Monaco	3 (1 to 7)	90.627 (27.075 to 194.637)	4 (2 to 10)	88.084 (30.616 to 196.143)	0.373 (-0.559 to 2.895)	-0.3 (-0.42 to -0.19)
Mongolia	2548 (1533 to 3828)	283.102 (170.307 to 425.3)	1809 (779 to 3308)	166.478 (71.654 to 304.46)	-0.29 (-0.711 to 0.531)	-1.98 (-2.11 to -1.86)
Montenegro	220 (96 to 428)	135.922 (59.39 to 265.054)	118 (37 to 253)	105.504 (33.348 to 227.344)	-0.465 (-0.808 to 0.367)	-1.05 (-1.17 to -0.94)
Morocco	27669 (17213 to 43868)	282.744 (175.896 to 448.28)	18616 (9241 to 31826)	190.12 (94.372 to 325.026)	-0.327 (-0.675 to 0.217)	-1.03 (-1.22 to -0.84)
Mozambique	28676 (17486 to 42880)	462.201 (281.84 to 691.146)	51546 (28693 to 80371)	361.318 (201.13 to 563.37)	0.798 (-0.089 to 2.372)	-0.66 (-0.85 to -0.48)
Myanmar	20740 (6213 to 42167)	140.361 (42.047 to 285.368)	20510 (6723 to 40658)	131.349 (43.057 to 260.384)	-0.011 (-0.691 to 2.515)	-0.23 (-0.3 to -0.16)
Namibia	1242 (610 to 2066)	206.72 (101.566 to 343.841)	1765 (905 to 2956)	213.822 (109.672 to 358.158)	0.421 (-0.37 to 1.962)	0.17 (0.1 to 0.25)
Nauru	7 (2 to 13)	163.209 (59.122 to 303.852)	6 (2 to 10)	148.223 (60.906 to 262.428)	-0.144 (-0.68 to 1.509)	-0.38 (-0.49 to -0.26)
Nepal	33575 (16974 to 55594)	398.51 (201.467 to 659.851)	21221 (10791 to 36890)	229.979 (116.945 to 399.792)	-0.368 (-0.695 to 0.49)	-1.64 (-1.78 to -1.51)
Netherlands	2582 (1341 to 4483)	94.754 (49.219 to 164.488)	2806 (1326 to 5429)	104.61 (49.423 to 202.418)	0.086 (-0.526 to 1.748)	0.5 (0.21 to 0.79)
New Zealand	1269 (771 to 2046)	158.578 (96.326 to 255.69)	988 (491 to 1824)	100.632 (49.979 to 185.807)	-0.221 (-0.595 to 0.442)	-1.47 (-1.56 to -1.38)
Nicaragua	5569 (2975 to 8740)	305.784 (163.334 to 479.882)	3132 (1333 to 6132)	158.142 (67.309 to 309.62)	-0.438 (-0.785 to 0.313)	-2.02 (-2.11 to -1.93)
Niger	8886 (3579 to 17500)	218.72 (88.099 to 430.713)	21444 (8274 to 42337)	168.003 (64.824 to 331.69)	1.413 (-0.101 to 6.112)	-1 (-1.09 to -0.9)
Nigeria	79548 (49494 to 122244)	203.319 (126.503 to 312.45)	191648 (127455 to 287236)	188.65 (125.461 to 282.743)	1.409 (0.89 to 2.193)	-0.19 (-0.39 to 0.01)
Niue	1 (0 to 2)	134.01 (47.042 to 247.908)	1 (0 to 1)	204.443 (124.068 to 317.053)	-0.268 (-0.631 to 1.017)	0.39 (0.06 to 0.72)
North Macedonia	1029 (552 to 1778)	195.314 (104.7 to 337.574)	397 (162 to 760)	121.041 (49.431 to 232.072)	-0.615 (-0.853 to -0.142)	-1.52 (-1.65 to -1.4)
Northern Mariana Islands	12 (3 to 27)	102.12 (23.144 to 226.078)	11 (3 to 23)	100.005 (29.006 to 204.793)	-0.093 (-0.733 to 2.945)	0.01 (-0.1 to 0.12)
Norway	1121 (743 to 1710)	140.356 (93.014 to 214.164)	973 (532 to 1707)	105.34 (57.59 to 184.779)	-0.132 (-0.402 to 0.196)	-0.82 (-0.98 to -0.66)
Oman	1254 (421 to 2521)	149.237 (50.083 to 299.977)	1598 (514 to 3128)	130.702 (41.987 to 255.755)	0.275 (-0.631 to 2.954)	-0.44 (-0.54 to -0.33)
Pakistan	154702 (106197 to 220894)	314.167 (215.663 to 448.589)	255654 (186130 to 345716)	299.213 (217.844 to 404.62)	0.653 (0.177 to 1.418)	0.18 (0.03 to 0.33)
Palau	7 (3 to 12)	155.246 (62.85 to 272.023)	4 (2 to 8)	129.536 (51.481 to 231.723)	-0.403 (-0.771 to 0.579)	-0.5 (-0.6 to -0.39)
Palestine	2936 (1777 to 4481)	303.219 (183.485 to 462.82)	3496 (1970 to 5624)	187.254 (105.511 to 301.18)	0.191 (-0.389 to 1.222)	-1.37 (-1.5 to -1.24)
Panama	1881 (954 to 3209)	225.606 (114.408 to 384.81)	2237 (958 to 4177)	193.953 (83.032 to 362.195)	0.189 (-0.492 to 2)	-0.39 (-0.45 to -0.33)
Papua New Guinea	2101 (827 to 3995)	123.597 (48.637 to 234.988)	5121 (2321 to 9034)	130.729 (59.26 to 230.624)	1.437 (0.027 to 5.116)	0.18 (0.07 to 0.29)
Paraguay	3275 (1704 to 5453)	196.13 (102.033 to 326.611)	3356 (1664 to 5698)	167.15 (82.88 to 283.806)	0.025 (-0.501 to 1.245)	-0.41 (-0.48 to -0.34)

Peru	23972 (10734 to 40238)	288.791 (129.316 to 484.744)	15673 (5985 to 30793)	164.33 (62.755 to 322.87)	-0.346 (-0.759 to 0.733)	-2.65 (-2.95 to -2.35)
Philippines	32551 (20405 to 49181)	129.099 (80.926 to 195.053)	40319 (26733 to 60423)	118.589 (78.629 to 177.721)	0.239 (0.032 to 0.513)	-0.14 (-0.2 to -0.09)
Poland	10670 (6976 to 15946)	111.418 (72.844 to 166.508)	6215 (3879 to 9836)	105.59 (65.913 to 167.11)	-0.418 (-0.571 to -0.236)	0.03 (-0.1 to 0.15)
Portugal	2575 (1388 to 4528)	121.681 (65.577 to 214.02)	1277 (631 to 2420)	93.76 (46.318 to 177.613)	-0.504 (-0.769 to 0.134)	-1.1 (-1.25 to -0.95)
Puerto Rico	1680 (722 to 3003)	168.721 (72.556 to 301.534)	541 (150 to 1176)	121.847 (33.662 to 264.714)	-0.678 (-0.906 to -0.23)	-1.26 (-1.33 to -1.19)
Qatar	316 (138 to 581)	252.414 (110.315 to 464.609)	687 (231 to 1478)	139.103 (46.682 to 299.198)	1.177 (-0.362 to 4.437)	-1.99 (-2.03 to -1.94)
Republic of Korea	21595 (11654 to 34980)	189.922 (102.491 to 307.639)	6637 (2791 to 13332)	109.253 (45.94 to 219.479)	-0.693 (-0.871 to -0.295)	-2 (-2.12 to -1.89)
Republic of Moldova	2611 (1756 to 3882)	211.28 (142.111 to 314.074)	569 (308 to 1003)	108.866 (58.91 to 192.02)	-0.782 (-0.89 to -0.601)	-2.66 (-3.07 to -2.24)
Romania	12367 (6853 to 20513)	222.113 (123.072 to 368.399)	4525 (2164 to 7999)	150.317 (71.908 to 265.748)	-0.634 (-0.834 to -0.253)	-1.15 (-1.24 to -1.06)
Russian Federation	35235 (24424 to 51633)	101.546 (70.388 to 148.806)	17190 (10429 to 28958)	65.92 (39.991 to 111.045)	-0.512 (-0.625 to -0.403)	-1.98 (-2.16 to -1.8)
Rwanda	14767 (8476 to 23069)	435.234 (249.826 to 679.918)	14905 (8695 to 23726)	299.884 (174.932 to 477.354)	0.009 (-0.476 to 0.859)	-1.4 (-1.56 to -1.24)
Saint Kitts and Nevis	44 (23 to 71)	311.451 (165.086 to 506.19)	21 (10 to 37)	211.906 (100.842 to 380.49)	-0.526 (-0.796 to 0.075)	-1.21 (-1.31 to -1.11)
Saint Lucia	137 (73 to 225)	264.958 (142.065 to 436.951)	56 (26 to 106)	189.217 (88.661 to 355.663)	-0.589 (-0.824 to -0.021)	-1.18 (-1.25 to -1.12)
Saint Vincent and the Grenadines	106 (57 to 177)	258.027 (139.6 to 431.38)	52 (26 to 89)	209.045 (105.367 to 355.394)	-0.508 (-0.773 to 0.014)	-0.86 (-1.01 to -0.71)
Samoa	87 (32 to 162)	122.004 (44.364 to 227.678)	88 (33 to 171)	109.682 (41.077 to 214.127)	0.009 (-0.618 to 1.841)	-0.39 (-0.5 to -0.27)
San Marino	3 (1 to 7)	80.779 (24.275 to 178.566)	3 (1 to 8)	74.919 (19.715 to 173.715)	-0.005 (-0.707 to 2.264)	-0.3 (-0.37 to -0.22)
Sao Tome and Principe	121 (48 to 222)	214.255 (84.053 to 392.311)	141 (49 to 262)	180.622 (62.902 to 337.028)	0.158 (-0.613 to 2.506)	-0.45 (-0.65 to -0.25)
Saudi Arabia	19238 (10088 to 30640)	293.564 (153.942 to 467.54)	14547 (5472 to 28759)	192.292 (72.335 to 380.149)	-0.244 (-0.709 to 0.663)	-1.57 (-1.63 to -1.52)
Senegal	8574 (3280 to 16159)	234.829 (89.839 to 442.559)	12345 (4850 to 22567)	194.085 (76.254 to 354.8)	0.44 (-0.547 to 3.414)	-0.42 (-0.5 to -0.34)
Serbia	4828 (2222 to 8116)	222.607 (102.448 to 374.228)	1608 (689 to 3360)	121.096 (51.878 to 253.08)	-0.667 (-0.865 to -0.216)	-2.1 (-2.27 to -1.94)
Seychelles	35 (15 to 64)	148.441 (63.067 to 271.224)	30 (10 to 60)	129.086 (41.999 to 254.907)	-0.142 (-0.719 to 1.082)	-0.22 (-0.37 to -0.07)
Sierra Leone	4418 (1582 to 8306)	243.761 (87.301 to 458.204)	6990 (2639 to 12640)	195.455 (73.798 to 353.456)	0.582 (-0.403 to 3.335)	-0.73 (-0.83 to -0.63)
Singapore	645 (264 to 1254)	99.283 (40.669 to 193.179)	677 (258 to 1468)	83.41 (31.784 to 180.731)	0.051 (-0.661 to 2.066)	-0.49 (-0.56 to -0.43)
Slovakia	2259 (1113 to 3975)	170.418 (83.936 to 299.856)	1160 (498 to 2209)	135.483 (58.094 to 257.885)	-0.486 (-0.779 to 0.23)	-0.76 (-0.86 to -0.66)
Slovenia	592 (255 to 1129)	143.074 (61.713 to 272.99)	320 (116 to 721)	102.535 (37.252 to 230.977)	-0.459 (-0.813 to 0.471)	-1.15 (-1.22 to -1.07)
Solomon Islands	184 (72 to 355)	118.105 (46.051 to 228.15)	299 (125 to 533)	114.97 (48.052 to 205.051)	0.626 (-0.358 to 3.189)	-0.08 (-0.2 to 0.04)
Somalia	13628 (8039 to 20717)	349.817 (206.348 to 531.797)	28677 (14874 to 46346)	277.628 (143.998 to 448.68)	1.104 (0.156 to 2.464)	-0.57 (-0.79 to -0.36)
South Africa	31218 (22196 to 43204)	229.316 (163.045 to 317.359)	27673 (19245 to 40433)	181.997 (126.566 to 265.916)	-0.114 (-0.35 to 0.203)	-0.93 (-1.11 to -0.74)
South Sudan	10576 (5978 to 16918)	403.007 (227.816 to 644.72)	15822 (9990 to 23869)	368.379 (232.597 to 555.745)	0.496 (-0.177 to 1.769)	-0.2 (-0.41 to 0)
Spain	7473 (2783 to 16520)	95.372 (35.521 to 210.823)	5394 (2213 to 11987)	83.224 (34.143 to 184.956)	-0.278 (-0.732 to 1.01)	-0.27 (-0.41 to -0.12)
Sri Lanka	14319 (7128 to 25696)	258.79 (128.819 to 464.417)	8411 (3216 to 16288)	164.788 (63.016 to 319.132)	-0.413 (-0.783 to 0.431)	-1.58 (-1.77 to -1.38)
Sudan	34463 (18434 to 55012)	387.556 (207.294 to 618.635)	39253 (23321 to 60555)	236.619 (140.58 to 365.028)	0.139 (-0.388 to 1.246)	-1.44 (-1.59 to -1.3)
Suriname	382 (171 to 637)	293.041 (131.06 to 489.079)	327 (174 to 540)	228.269 (121.27 to 376.999)	-0.143 (-0.589 to 1.092)	-0.72 (-0.83 to -0.62)
Sweden	1261 (594 to 2424)	81.623 (38.443 to 156.952)	1172 (501 to 2343)	64.364 (27.542 to 128.691)	-0.07 (-0.596 to 1.073)	-0.58 (-0.7 to -0.47)
Switzerland	1333 (560 to 2692)	115.372 (48.494 to 232.954)	1233 (492 to 2506)	92.55 (36.922 to 188.104)	-0.075 (-0.674 to 1.48)	-0.7 (-0.78 to -0.63)
Syrian Arab Republic	10629 (5549 to 17780)	179.477 (93.699 to 300.236)	5516 (2910 to 10058)	150.567 (79.443 to 274.556)	-0.481 (-0.766 to 0.216)	-0.63 (-0.71 to -0.55)

Taiwan (Province of China)	6530 (3070 to 12350)	118.553 (55.729 to 224.209)	2938 (1254 to 5992)	99.718 (42.541 to 203.33)	-0.55 (-0.812 to 0.186)	-0.61 (-0.71 to -0.51)
Tajikistan	10050 (6313 to 15417)	432.827 (271.876 to 663.938)	12818 (7968 to 18489)	357.615 (222.308 to 515.815)	0.275 (-0.262 to 1.186)	-0.9 (-1.06 to -0.73)
Thailand	24869 (12035 to 42821)	147.518 (71.386 to 254.004)	13098 (5530 to 25321)	134.109 (56.626 to 259.273)	-0.473 (-0.782 to 0.317)	-0.37 (-0.41 to -0.32)
Timor-Leste	533 (211 to 971)	160.366 (63.46 to 292.043)	718 (288 to 1378)	137.943 (55.304 to 264.728)	0.346 (-0.461 to 2.713)	-0.48 (-0.56 to -0.39)
Togo	4947 (1567 to 9830)	280.676 (88.89 to 557.743)	6058 (2208 to 12050)	183.067 (66.74 to 364.156)	0.225 (-0.588 to 3.073)	-1.68 (-1.89 to -1.47)
Tokelau	1 (0 to 1)	125.78 (49.946 to 237.314)	1 (1 to 1)	250.797 (160.55 to 357.817)	0.296 (-0.391 to 2.369)	0.39 (-0.29 to 1.08)
Tonga	44 (16 to 83)	104.255 (37.566 to 198.555)	40 (16 to 74)	103.007 (40.498 to 188.815)	-0.078 (-0.646 to 1.652)	-0.22 (-0.35 to -0.09)
Trinidad and Tobago	1091 (603 to 1852)	268.482 (148.406 to 455.795)	518 (231 to 951)	190.228 (84.72 to 349.094)	-0.525 (-0.798 to 0.117)	-1.38 (-1.5 to -1.26)
Tunisia	8387 (4613 to 12799)	270.061 (148.529 to 412.152)	4104 (2012 to 7395)	148.391 (72.76 to 267.392)	-0.511 (-0.774 to 0.084)	-1.92 (-1.96 to -1.89)
Türkiye	96995 (55398 to 153098)	473.405 (270.382 to 747.229)	44830 (24344 to 76453)	242.057 (131.444 to 412.804)	-0.538 (-0.771 to -0.07)	-1.65 (-1.88 to -1.42)
Turkmenistan	4004 (2289 to 6471)	266.765 (152.515 to 431.124)	4018 (1907 to 6936)	263.633 (125.107 to 455.15)	0.003 (-0.562 to 1.132)	-0.26 (-0.49 to -0.04)
Tuvalu	5 (2 to 8)	133.915 (56.07 to 235.78)	4 (2 to 8)	110.788 (47.974 to 207.042)	-0.113 (-0.66 to 1.38)	-0.69 (-0.79 to -0.6)
Uganda	28422 (15589 to 48000)	337.57 (185.153 to 570.102)	59386 (32063 to 96934)	299.389 (161.644 to 488.686)	1.089 (0.027 to 3.156)	-0.34 (-0.47 to -0.21)
Ukraine	13355 (6849 to 23878)	117.409 (60.212 to 209.921)	5247 (2227 to 10356)	82.69 (35.094 to 163.202)	-0.607 (-0.847 to -0.064)	-1.99 (-2.26 to -1.71)
United Arab Emirates	1829 (792 to 3218)	310.297 (134.394 to 545.943)	2257 (847 to 4700)	168.591 (63.269 to 351.096)	0.234 (-0.559 to 2.146)	-2.03 (-2.17 to -1.9)
United Kingdom	15717 (10875 to 22195)	143.92 (99.581 to 203.244)	13611 (8044 to 22432)	115.514 (68.264 to 190.378)	-0.134 (-0.318 to 0.046)	-0.74 (-0.84 to -0.63)
United Republic of Tanzania	52333 (34912 to 74411)	433.379 (289.113 to 616.217)	92704 (55177 to 145177)	379.891 (226.108 to 594.916)	0.771 (0.041 to 2.161)	-0.25 (-0.36 to -0.14)
United States of America	52304 (29739 to 84222)	93.55 (53.191 to 150.638)	56121 (32960 to 96776)	94.425 (55.456 to 162.828)	0.073 (-0.168 to 0.38)	0.09 (0.04 to 0.15)
United States Virgin Islands	62 (24 to 111)	193.067 (75.692 to 348.418)	19 (6 to 38)	145.211 (45.935 to 284.855)	-0.685 (-0.907 to -0.119)	-0.95 (-1.04 to -0.86)
Uruguay	1451 (695 to 2617)	177.215 (84.843 to 319.681)	898 (359 to 1672)	136.123 (54.472 to 253.455)	-0.381 (-0.773 to 0.488)	-0.68 (-0.77 to -0.59)
Uzbekistan	22118 (13070 to 35185)	258.522 (152.768 to 411.25)	27990 (18379 to 43452)	277.372 (182.129 to 430.595)	0.265 (-0.264 to 1.401)	0.12 (-0.15 to 0.4)
Vanuatu	74 (29 to 138)	109.038 (41.971 to 203.334)	129 (50 to 237)	110.62 (42.62 to 203.642)	0.736 (-0.392 to 3.774)	0.09 (-0.07 to 0.24)
Venezuela (Bolivarian Republic of)	17525 (8532 to 30447)	247.043 (120.277 to 429.198)	13458 (6362 to 23924)	203.169 (96.041 to 361.181)	-0.232 (-0.655 to 0.854)	-0.55 (-0.71 to -0.39)
Viet Nam	21103 (4769 to 48684)	79.597 (17.989 to 183.628)	21332 (4853 to 49390)	86.147 (19.596 to 199.453)	0.011 (-0.798 to 3.463)	0.28 (0.06 to 0.49)
Yemen	22325 (10276 to 36374)	314.684 (144.843 to 512.718)	29710 (16281 to 47218)	215.47 (118.074 to 342.447)	0.331 (-0.281 to 1.78)	-1.15 (-1.31 to -0.99)
Zambia	18631 (10141 to 28292)	496.223 (270.092 to 753.529)	33361 (19458 to 52699)	403.326 (235.243 to 637.115)	0.791 (-0.051 to 2.29)	-0.84 (-0.99 to -0.68)
Zimbabwe	9711 (5148 to 16723)	201.635 (106.893 to 347.22)	14534 (8180 to 22999)	230.916 (129.976 to 365.421)	0.497 (-0.296 to 2.072)	0.59 (0.43 to 0.75)

Abbreviations: EAPC, estimated annual percentage change; SDI, Sociodemographic Index; UI, uncertainty interval.

[a] EAPC is expressed as 95% Cls.
